# Efficacy and Safety of Oral Chinese Patent Medicine Combined with Conventional Therapy for Heart Failure: An Overview of Systematic Reviews

**DOI:** 10.1155/2020/8620186

**Published:** 2020-08-27

**Authors:** Shan-Shan Lin, Chun-Xiang Liu, Jun-Hua Zhang, Xian-Liang Wang, Jing-Yuan Mao

**Affiliations:** ^1^Cardiovascular Department, First Teaching Hospital of Tianjin University of Traditional Chinese Medicine, Tianjin 300381, China; ^2^Evidence-Based Medicine Center, Tianjin University of Traditional Chinese Medicine, Tianjin 301617, China

## Abstract

**Objectives:**

By performing an overview of systematic reviews and meta-analyses of the efficacy and safety of oral Chinese patent medicine combined with conventional therapy in the treatment of heart failure, to evaluate the reliability and applicability of the conclusions of the current studies and provide evidence for clinical decision-making.

**Methods:**

Systematic reviews and meta-analyses of oral Chinese patent medicine combined with conventional therapy treating heart failure were searched based on standardized search strategy in six electronic databases including PubMed, Embase, Cochrane Library (No. 2 of 2020), China National Knowledge Infrastructure (CNKI), Wanfang Database (Wanfang), and Chinese Scientific Journal Database (VIP) from inception to February 2020. The literature was independently screened and extracted by two researchers. The methodological quality of the included literature was evaluated using the AMSTAR-2 (A Measurement Tool to Assess Systematic Review 2). If necessary, we would summarize the original research data and further perform data synthesis using RevMan software (version 5.3), and the evidence quality of the included literature was graded using the Grading of Recommendations Assessment, Development and Evaluation (GRADE) system.

**Results:**

A total of 38 systematic reviews and meta-analyses were included, involving 11 kinds of oral Chinese patent medicines, including Qili Qiangxin Capsules (11/38), Qishen Yiqi Dropping Pills (9/38), Shexiang Baoxin Pills (4/38), Wenxin Keli (2/38), Tongxinluo Capsules (2/38), Compound Danshen Dripping Pills (2/38), Zhenyuan Capsules (3/38), Buyi Qiangxin Tablets (2/38), Yangxinshi Tablets (1/38), Xuezhikang (1/38), and Yixinshu Capsules (1/38). The methodological quality of all literature was rated as critically low. The grading of the quality of evidence was 43 moderate, 101 low, and 40 very low. The main reason for the degradation of evidence quality was the risk of bias. In the evaluation of efficacy, there was no statistically significant difference between the two groups in terms of mortality, which is a piece of low-quality evidence. Qili Qiangxin Capsules or Qishen Yiqi Dripping Pills combined with conventional therapy can significantly reduce the hospitalization rate of patients with chronic heart failure, and the quality of the evidence is moderate. The overall efficacy of oral Chinese patent medicine combined with conventional therapy in improving the clinical symptoms, quality of life, exercise endurance, laboratory tests, physical examination, and other indicators of patients with heart failure is confirmed. In the evaluation of safety, there was no significant difference between the two groups.

**Conclusions:**

Oral Chinese patent medicine combined with conventional therapy has good clinical efficacy and safety in the treatment of heart failure. However, due to its low level of methodological quality and evidence quality, the current evidence-based conclusions need to be further verified.

## 1. Introduction

Heart failure (HF) is a disorder of ventricular contraction and/or diastolic function caused by abnormal changes in the structure and/or function of the heart, which causes a series of complex clinical syndromes [1]. HF is the end-stage of cardiovascular disease. Because of its complex condition and poor prognosis, it has become an important public health issue globally [2]. Traditional Chinese medicine (TCM) has a history of thousands of years and it has an irreplaceable position in China's medical care. At present, a series of systematic reviews and meta-analyses have shown that TCM combined with conventional therapy (CT) can improve the curative effect [3, 4]. Based on the foundation of modern pharmacological research, more and more Chinese medicine extracts are used in modern pharmaceutical preparations, such as oral Chinese patent medicine (OCPM) and Chinese medicine injection. Modern pharmaceutical preparations have the advantages of clear indications and convenient application. But in terms of safety, long-term observation is still needed.

Systematic review and meta-analysis are important research methods in evidence-based medicine and the best source of clinical evidence [5, 6]. However, the quality of some systematic reviews and meta-analyses is uneven [7, 8]. Therefore, more attention needs to be paid to the quality of evidence. The overview is a comprehensive research method that can be used to comprehensively collect and summarize the systematic reviews and meta-analyses on different aspects of the same field, such as the etiology, diagnosis, and treatment of a disease or health problem [9]. It can increase the breadth and depth of evidence. This study conducted an overview of systematic reviews and meta-analyses of the efficacy and safety of OCPM combined with CT for HF, to evaluate the authenticity and reliability of evidence-based evidence and provide more comprehensive and more convincing evidence for clinical decision-making. The overview was constructed following the PRIO-harms checklist (Supplementary Material 1).

## 2. Methods

### 2.1. Inclusion Criteria

#### 2.1.1. Types of Reviews

Systematic reviews and meta-analyses of clinical randomized controlled trials (RCTs) were included. The language is limited to Chinese and English.

#### 2.1.2. Types of Participants

The participants met the recognized diagnostic criteria for HF, regardless of gender, age, race, time of onset, and source of cases.

#### 2.1.3. Types of Interventions

The types of intervention include oral Chinese patent medicine (OCPM) + conventional therapy (CT) vs. CT, OCPM + CT vs. placebo + CT, or OCPM + CT vs. a designated positive control drug + CT. In the treatment group and the control group, the principle of CT is the same. These OCPMs must be included in the Pharmacopoeia of the People's Republic of China [10] or the data query system of the China Food and Drug Administration website http://www.nmpa.gov.cn/WS04/CL2042/.

#### 2.1.4. Types of Outcomes

Studies using at least one of the following outcome indicators were included.

- Primary efficacy outcome indicators: (a) major adverse cardiovascular events (MACE), such as mortality, hospitalization rate; (b) clinical comprehensive efficacy
- Secondary efficacy outcome indicators: (a) New York Heart Association (NYHA) cardiac function efficacy; (b) quality of life (QOL) scores; (c) 6-minute walk test (6-MWT); (d) brain natriuretic peptide (BNP)/N-terminal probrain natriuretic peptide (NT-proBNP); (e) ultrasonic cardiogram, such as left ventricular ejection fraction (LVEF), left ventricular end-diastolic diameter (LVEDD), and left ventricular end-systolic diameter (LVESD)
- Safety outcome indicator: adverse events including dizziness, dry cough, palpitation, gastrointestinal intolerance, itchy skin, or rash

### 2.2. Exclusion Criteria

- For studies that are repeatedly published by different centers, we only included the one with the most complete results and the highest quality
- The full text of the literature could not be obtained by asking for help
- The complete and correct data could not be obtained by contacting the author
- Unfinished protocol
- Systematic review without meta-analysis
- Network meta-analysis

### 2.3. Search Strategy

The systematic review and meta-analyses of OCPM for HF were searched in the relevant database, including PubMed, Embase, Cochrane Library (No. 2 of 2020), China National Knowledge Infrastructure (CNKI), Wanfang Database (Wanfang), and Chinese Scientific Journal Database (VIP). The retrieval time was from inception to February 2020. The search terms mainly included heart failure, systematic review, meta-analysis, Chinese patent medicine, Chinese herbal medicine, and their synonyms. The search strategy adopted a combination of Medical Subject Heading and free-text terms and adopted different search strategies according to the characteristics of each database. The synonyms in the group were connected by “or”, and the search terms between the groups were connected by “and.” At the same time, the reference lists of existing systematic reviews and meta-analyses, protocol registries, and conference abstracts were further searched to avoid omissions. The language of the literature was not limited. The search strategy was developed and implemented by Lin, a researcher with clinical work experience, and Liu, a researcher with evidence-based work experience, and further revised by library staff. Take PubMed as an example. The detailed search strategy in PubMed is shown in Supplementary Material 2.

### 2.4. Literature Screening and Data Collection

Two researchers (a clinician Lin and a methodologist Liu) independently conducted literature management and screening through NoteExpress software (version 3.2.0). Because this study focuses on all types of HF patients, even if there may be overlaps, we will still include all eligible systematic reviews to avoid missing important information. The extracted data is saved in a database created by Excel software. The data extraction items included the title, author, journal name, publication year, disease diagnosis criteria, number of included studies and total sample size, intervention measures, outcome indicators, methodological quality evaluation tools, authors' main conclusions, etc. When the data was incomplete or suspected to be incorrect, we would obtain the correct data from the relevant RCTs of included reviews. Cross-checking would be performed after the data extraction was completed. In case of disagreement, it would be decided by discussion between the two parties or judged by the third evaluator (Wang).

### 2.5. Quality Evaluation of Included Literature

#### 2.5.1. Methodological Quality Evaluation

Two researchers (Lin and Liu) independently conducted the methodological quality evaluation of the included studies using AMSTAR-2 (A Measurement Tool to Assess Systematic Review 2). The scale includes a total of 16 entries, each of which is assessed by “yes,” “partially yes,” “no,” or “no meta-analysis conducted.” The AMSTAR-2 research team selected seven key items that influenced the production and result validity of systematic review and meta-analysis, including entries 2, 4, 7, 9, 11, 13, and 15. The grading of methodological quality is based on the “confidence intensity” provided by the evaluators. “High” indicates that the results of the study provide an accurate and comprehensive summary, “moderate” indicates possible, “low” indicates that it may not, and “critically low” indicates impossible. Cross-checking would be performed after the evaluation was completed. In case of disagreement, it would be decided by discussion between the two parties or judged by the third evaluator (Wang) [11, 12].

#### 2.5.2. Grading of the Evidence Quality

According to the Grading of Recommendations Assessment, Development and Evaluation (GRADE) system, the two researchers (Lin and Liu) independently performed the quality assessment and recommendation grading of the evidence. Reasons for downgrading include five aspects: study limitations (risk of bias) [13], inconsistency [14], and indirectness [15], imprecision [16], publication bias [17]. According to the Cochrane Collaboration's tool for assessing the risk of bias in randomized trials, the evidence quality is rated in four levels including high, moderate, low, or very low. High indicates that the authors are very confident that the real effect is close to the estimate of the effect. Moderate indicates that the authors are moderately confident in the effect estimate: the true effect is likely to be close to the estimate of the effect, but there is a possibility that it is substantially different. Low indicates that the authors' confidence in the effect estimate is limited: the true effect may be substantially different from the estimate of the effect. Very low indicates that the authors have very little confidence in the effect estimate: the true effect is likely to be substantially different from the estimate of effect [18]. Cross-checking would be performed after the classification was completed. In case of disagreement, it would be decided by discussion between the two parties or judged by the third evaluator (Wang).

### 2.6. Data Synthesis

If necessary, we would summarize the original research data and further perform data synthesis using RevMan software (version 5.3). Dichotomous variables will be presented as the relative risk (RR) or odds ratio (OR) with a 95% confidence interval (CI). If zero events were included in the studies, risk difference (RD) would be used. Continuous variables will be presented as the weight mean difference (WMD) with a 95% CI. *P* < 0.05 was considered to be statistically significant. The statistical heterogeneity was estimated according to *I*^2^ statistics. If the results had no statistical heterogeneity (*P* > 0.1, *I*^2^ < 50%), a fixed-effect model would be used. When there was statistical heterogeneity (*P* < 0.1, *I*^2^ > 50%), the subgroup analysis would be needed if there was obvious clinical or methodological heterogeneity. If no obvious clinical or methodological heterogeneity was found, a random-effect model would be used. When data cannot be synthesized, only nonquantitative descriptive analysis was performed. If the number of included studies is sufficient (>10 articles), Stata software (version 15.0) can be used to draw an inverted funnel chart to qualitatively analyze for potential publication bias or perform an Egger test to further quantitatively analyze the possibility of publication bias.

## 3. Results

### 3.1. Literature Screening Results

**Figure 1:**
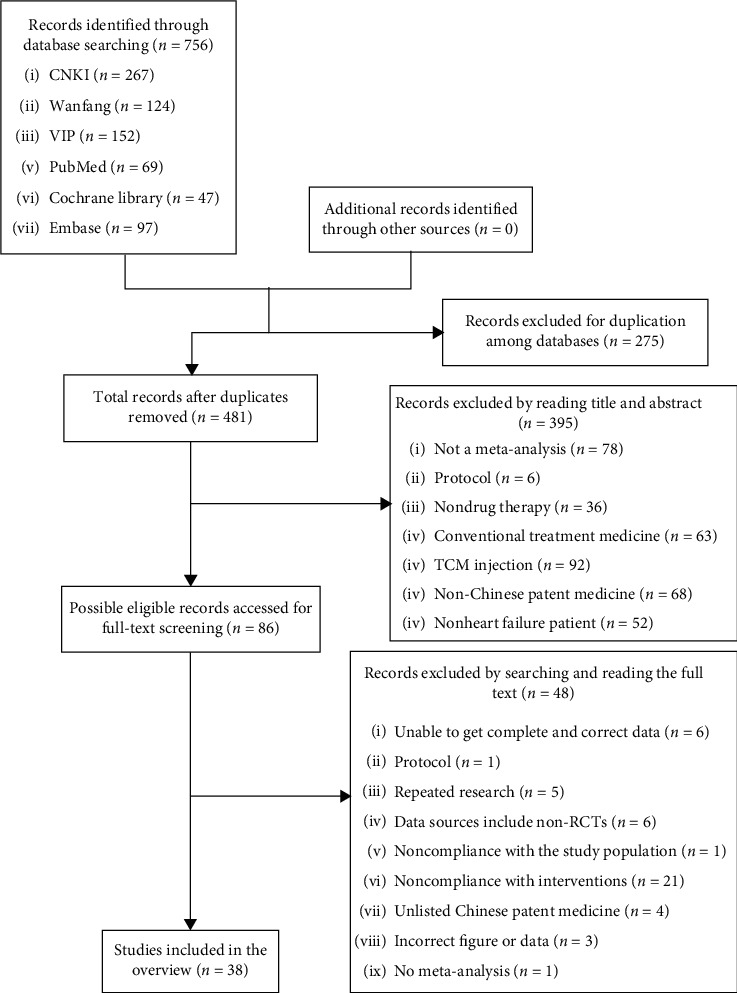
Flowchart of the literature screening process.

A total of 756 literature were retrieved, and 86 possible related literature were screened out by reading the abstract. After reading the full text, 38 systematic reviews and meta-analyses were finally included. The literature screening process and results are shown in Figure 1.

### 3.2. Basic Characteristics of the Included Literature

A total of 38 pieces of literature published in 2010–2019 were included [19–56]. The number of literature published in the past five years accounts for 78.95% (30/38). The diseases studied include HF, chronic heart failure (CHF), heart failure with preserved ejection fraction (HFpEF), diastolic heart failure (DHF), heart failure caused by ischemic cardiomyopathy (HF-IMC), heart failure caused by coronary heart disease (HF-CHD), and HF with arrhythmia. Interventions include OCPM + CT vs. CT, OCPM + CT vs. placebo + CT. This study involved 11 kinds of qualified OCPMs, including Qili Qiangxin Capsules (11/38) [19–29], Qishen Yiqi Dropping Pills (9/38) [30–38], Shexiang Baoxin Pills (4/38) [39–42], Wenxin Keli (2/38) [43, 44], Tongxinluo Capsules (2/38) [45, 46], Compound Danshen Dripping Pills (2/38) [47, 48], Zhenyuan Capsules (3/38) [49–51], Buyi Qiangxin Tablets (2/38) [52, 53], Yangxinshi Tablets (1/38) [54], Xuezhikang (1/38) [55], and Yixinshu Capsules (1/38) [56]. The number of included original studies ranged from 5 to 120. The study mentioned 24 outcome indicators. Twenty-five systematic reviews were evaluated according to the Cochrane Handbook and 13 systematic reviews were evaluated according to the Jadad scale. Details are shown in Table 1.

### 3.3. Methodological Quality Evaluation

**Figure 2:**
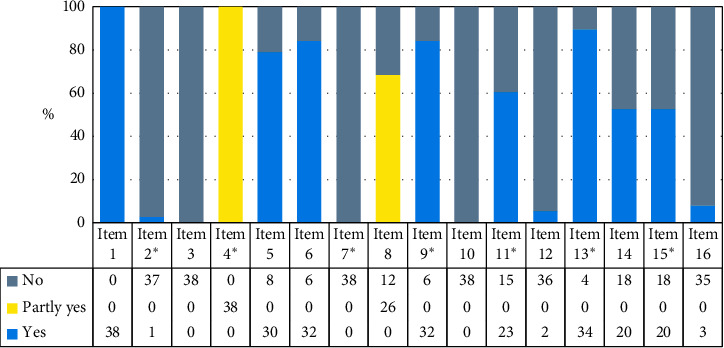
Percentage results of methodological quality evaluation of included studies. ^*∗*^Refers to a key item. Item 1: did the research questions and inclusion criteria for the review include the components of PICO? Item 2: did the report of the review contain an explicit statement that the review methods were established prior to the conduct of the review, and did the report justify any significant deviations from the protocol? Item 3: did the review authors explain their selection of the study designs for inclusion in the review? Item 4: did the review authors use a comprehensive literature search strategy? Item 5: did the review authors perform study selection in duplicate? Item 6: did the review authors perform data extraction in duplicate? Item 7: did the review authors provide a list of excluded studies and justify the exclusions? Item 8: did the review authors describe the included studies in adequate detail? Item 9: did the review authors use a satisfactory technique for assessing the risk of bias (RoB) in individual studies that were included in the review? Item 10: did the review authors report on the sources of funding for the studies included in the review? Item 11: if meta-analysis was performed, did the review authors use appropriate methods for statistical combination of results? Item 12: if meta-analysis was performed, did the review authors assess the potential impact of RoB in individual studies on the results of the meta-analysis or other evidence synthesis? Item 13: did the review authors account for RoB in individual studies when interpreting/discussing the results of the review? Item 14: did the review authors provide a satisfactory explanation for, and discussion of, any heterogeneity observed in the results of the review? Item 15: if they performed quantitative synthesis, did the review authors carry out an adequate investigation of publication bias (small study bias) and discuss its likely impact on the results of the review? Item 16: did the review authors report any potential sources of conflict of interest, including any funding they received for conducting the review?

The methodological quality of the literature was evaluated using the AMSTAR-2.  Figure 2 shows that there are serious incomplete reports in key items 2, 4, 7, and nonkey items 3, 10, 12, 16. Regarding item 2 “did the report of the review contain an explicit statement that the review methods were established prior to the conduct of the review and did the report justify any significant deviations from the protocol?,” only one English literature provided the Protocol. Regarding item 4 “did the review authors use a comprehensive literature search strategy?,” all of the results were “partially.” All the included studies expounded basic information such as the databases, keyword or search strategy, publication restrictions, but few reviewers searched the reference lists of included studies and study registries, searched for gray literature, and consulted content experts in the field. Regarding item 7 “did the review authors provide a list of excluded studies and justify the exclusions?,” none of them provided. The above shortcomings were the main reason for the degradation of methodological quality. Besides, regarding item 3, all literature did not explain the selection of the study designs. Regarding item 10, all literature did not report the sources of funding for the studies included in the review. Regarding item 12, only two review authors assessed the potential impact of RoB in individual studies on the results of the meta-analysis or other evidence synthesis. Regarding item 16, only four review authors of English-language literature reported any potential sources of conflict of interest, including any funding they received for conducting the review. In the end, we classify the methodological quality of 38 literature into “very low.” See Online Supplementary Material 3, Methodological quality evaluation of included studies, for details.

### 3.4. Grading of the Evidence Quality

All included literature mentioned 24 outcome indicators. The evidence quality was graded according to the GRADE system. The main downgrading factor of the quality of evidence is the limitation; that is, all studies have defects in at least one of the random sequence generation, allocation concealment, blinding method, incomplete outcome data, selective reporting, and other limitation. Secondary downgrading factors are inconsistency, inaccuracy, and publication bias. However, there is no evidence that it was downgraded for indirectness. The details are shown in the Online Supplementary Material 4: Summary of findings (SoFs) tables based on different systematic reviews.

#### 3.4.1. Primary Efficacy Outcome Indicators

*(1) Mortality*. One literature [29] performed a meta-analysis of mortality including 539 patients with HF in 6 RCTs. The intervention in the test group was Qili Qiangxin Capsules combined with CT, and the intervention in the control group was CT alone. The meta-analysis result showed a relative risk (RR) = 0.53 and 95% CI = [0.27, 1.07] (*P* > 0.05), indicating that the mortality rate of the test group was 0.53 (0.27 to 1.07) times that of the control group (Table 2). The CI crossed the invalid value of 1, so it has not been proven that there was a statistically significant difference between Qili Qiangxin Capsules combined with the CT group and the control group. The quality of evidence is low. And more clinical trials are needed to provide higher quality evidence.

*(2) Hospitalization Rate*. Three pieces of literature [29–31] performed a meta-analysis of hospitalization rate. Sun et al. [29] found that there was a statistically significant difference between the two groups (RR = 0.49, 95% CI [0.38, 0.64], *P* < 0.05). The result showed that compared with the control group, Qili Qiangxin Capsules combined with CT can significantly reduce the hospitalization rate of HF patients. Wang et al. [30] and Liu et al. [31] both found that there was a statistically significant difference between two groups (Wang: RR = 0.52, 95% CI [0.33, 0.81], *P*=0.004; Liu: odds ratio (OR) = 0.41, 95% CI [0.23, 0.72], *P*=0.002). The results showed that compared with the control group, Qishen Yiqi Dripping Pills combined with CT can significantly reduce the hospitalization rate of CHF patients. The quality of all three pieces of evidence is moderate.

*(3) Clinical Comprehensive Efficacy*. Twenty pieces of literature [20, 21, 27, 28, 30, 33, 38–42, 44–48, 50, 52, 54, 55] performed a meta-analysis of clinical comprehensive efficacy, including 4 pieces of evidence about Qili Qiangxin Capsules, 3 pieces of Qishen Yiqi Dripping Pills, 4 pieces of Shexiang Baoxin Pills, 1 piece of Wenxin Keli, 2 pieces of Tongxinluo Capsules, 2 pieces of Fufang Danshen Dripping Pills, 1 piece of Zhenyuan Capsules, 1 piece of Buyi Qiangxin Tablets, 1 piece of Yangxinshi Tablets, and 1 piece of Xuezhikang. However, the current research evidence has not yet fully demonstrated the role of Yixinshu Capsules in improving the clinical effectiveness of HF patients. Among them, there are 7 pieces of moderate-quality evidence and 13 pieces of low-quality evidence. In Table 2, the effects of different drugs on clinical comprehensive efficacy can be intuitively compared. According to the effect size, it can be judged that there are slight differences between different OCPMs, and the differences are not statistically significant. The results show that it has been proved that these OCPMs except Yixinshu Capsules combined with CT can significantly improve the clinical comprehensive efficacy of HF patients compared with the control group (*P* < 0.05).

#### 3.4.2. Secondary Efficacy Outcome Indicators

*(1) New York Heart Association (NYHA) Cardiac Function Efficacy*. Fourteen pieces of literature [19, 21, 25, 26, 29–33, 35, 50–53] performed a meta-analysis of the NYHA cardiac function efficacy, including 7 pieces of moderate-quality evidence [19, 26, 30, 33, 35, 52, 53] and 7 pieces of low-quality evidence [21, 25, 29, 31, 32, 50, 51]. Among them, a meta-analysis [29] of 4603 HF patients from 54 RCTs showed that there are no statistically significant differences between the test group and the control group in improving the NYHA cardiac function efficacy in HF patients (*P* > 0.05). However, a meta-analysis [21] of 4510 CHF patients from 51 RCTs showed that Qili Qiangxin Capsules combined with CT can significantly improve the NYHA cardiac function efficacy compared with the control group (*P* < 0.05). The quality of these two pieces of evidence is low due to the risk of bias and significant statistical heterogeneity. Other evidence showed statistically significant differences (*P* < 0.05), indicating that Qili Qiangxin Capsules, Qishen Yiqi Dripping Pills, Zhenyuan Capsules, Buyi Qiangxin Tablets combined with CT can significantly improve the NYHA cardiac function efficacy of HF patients compared with the control group.

*(2) Minnesota Life Heart Failure Quality of Life Questionnaire (MLHFQ) Score*. Seven pieces of literature [19–21, 23, 24, 52, 53] performed a meta-analysis on the MLHFQ score, including 6 pieces of low-quality evidence [19–21, 52, 53] and 1 piece of very low-quality evidence [24]. The results showed that the difference was statistically significant (*P* < 0.05). It shows that Qili Qiangxin Capsules or Buyi Qiangxin Tablets combined with CT may significantly reduce the MLHFQ score compared with the control group in treating CHF patients.

*(3) 6-Minute Walk Test (6-MWT)*. Twenty-one pieces of literature [19–21, 23–26, 28–30, 33, 35, 37–42, 48, 54, 56] focused on 6-MWT, including 9 pieces of moderate-quality evidence [20, 21, 23–25, 29, 33, 54, 56], 9 pieces of low-quality evidence [19, 28–30, 37, 39, 41, 42, 48], and 4 pieces of very low-quality evidence [26, 35, 38, 40]. The results showed that the difference was statistically significant (*P* < 0.05), indicating that OCPM combined with CT can significantly increase the 6-MWT of HF patients compared with the control group. Effective OCPMs include Qili Qiangxin Capsules, Qishen Yiqi Dripping Pills, Shexiang Baoxin Pills, Compound Danshen Dripping Pills, Yangxinshi Pills, and Yixinshu Capsules.

*(4) Brain Natriuretic Peptide (BNP)*. Twenty-one pieces of literature [19–26, 30, 33, 35, 36, 38–41, 43, 48, 50, 54, 55] performed a meta-analysis on BNP, including 12 pieces of low-quality evidence [19–22, 24–26, 33, 35, 40, 43, 50], and 9 pieces of very low-quality evidence [23, 30, 36, 38, 39, 41, 48, 54, 55]. The results showed that the difference was statistically significant (*P* < 0.05), suggesting that Tongxinluo Capsules, Buyi Qiangxin Tablets, and Yixinshu Capsules combined with CT may significantly reduce BNP in HF patients compared with the control group.

*(5) N-Terminal Probrain Natriuretic Peptide (NT-proBNP)*. Ten pieces of literature [20, 22, 23, 26, 27, 29, 49, 52–54] performed meta-analysis of NT-proBNP, including 7 pieces of low-quality evidence [20, 22, 23, 26, 27, 29, 49], and 4 pieces of very low-quality evidence [27, 52–54]. Among them, literature [27] conducted subgroup analysis based on different treatment courses, and the results showed that when the treatment course was 6 months, the treatment effect was better than that when the treatment course was 1 month. There are statistical differences between the two subgroups (*I*^2^ = 87.5%, *P*=0.005). The results showed that the difference was statistically significant (*P* < 0.05), indicating that Qili Qiangxin Capsules, Zhenyuan Capsules, Buyi Qiangxin Tablets, Yangxinshi Pills, and Yixinshu Capsules combined with CT may significantly reduce NT-proBNP.

*(6) Left Ventricular Ejection Fraction (LVEF)*. Thirty-two pieces of literature [19–25, 28–30, 33, 35–46, 48–56] focused on LVEF, including 6 pieces of moderate-quality evidence [30, 35, 37, 51–53], 19 pieces of low-quality evidence [19–21, 23, 28, 29, 33, 36, 39, 41–46, 48, 49, 55, 56], and 9 pieces of very low-quality evidence [22, 24, 25, 30, 38, 40, 50, 51, 54]. A meta-analysis [54] of 818 CHF patients from 8 RCTs showed that no statistically significant differences between Yangxinshi Pills are combined with CT group and CT group. The evidence quality is very low. Other evidence showed ten other OCPMs except Yixinshu Capsules combined with CT can significantly improve LVEF in HF patients (*P* < 0.05).

*(7) Left Ventricular End-Diastolic Diameter (LVEDD)*. Sixteen pieces of literature [19–22, 24, 28, 31, 33, 34, 39, 40, 42, 45, 48, 55, 56] performed a meta-analysis of LVEDD, including 4 pieces of moderate-quality evidence [28, 33, 39, 48], 8 pieces of low-quality evidence [19, 21, 24, 31, 34, 42, 45, 56], and 4 pieces of very low-quality evidence [20, 22, 40, 55]. The results showed that the difference was statistically significant (*P* < 0.05), indicating that OCPM combined with CT may significantly reduce LVEDD in HF patients compared with the control group. Effective drugs include Qili Qiangxin Capsules, Qishen Yiqi Dripping Pills, Shexiang Baoxin Pills, Tongxinluo Capsules, Compound Danshen Dripping Pills, Xuezhikang, and Yixinshu Capsules.

*(8) Left Ventricular End-Systolic Diameter (LVESD)*. Six pieces of literature [22, 31, 33, 34, 40, 48] performed meta-analysis of LVESD, including 3 pieces of moderate-quality evidence [33, 34, 40], 2 pieces of low-quality evidence [31, 48], and 1 pieces of very low-quality evidence [22]. The results showed that the difference was statistically significant (*P* < 0.05), indicating that OCPM combined with CT may significantly reduce LVESD in CHF patients compared with the control group. Effective drugs include Qili Qiangxin Capsules, Qishen Yiqi Dripping Pills, Shexiang Baoxin Pills, and Compound Danshen Dripping Pills.

*(9) Cardiac Output (CO)*. Eight pieces of literature [20, 21, 25, 31, 41, 46, 49, 50] performed meta-analysis of CO, including 1 pieces of moderate-quality evidence [46], 5 pieces of low-quality evidence [25, 31, 41, 49, 50], and 2 pieces of very low-quality evidence [20. 21]. The results showed that the difference was statistically significant (*P* < 0.05), indicating that OCPM combined with CT may significantly increase CO in HF patients compared with the control group. Effective drugs include Qili Qiangxin Capsules, Qishen Yiqi Dripping Pills, Shexiang Baoxin Pills, Tongxinluo Capsules, and Zhenyuan Capsules.

*(10) Stroke Volume (SV)*. Three pieces of literature [41, 48, 50] performed a meta-analysis on improving SV. The results showed that the difference was statistically significant (*P* < 0.05). The evidence that Shexiang Baoxin Pills is valid is low-quality [41]. The evidence that Zhenyuan Capsules is valid is low-quality [50]. The evidence that Compound Danshen Dripping Pills is valid is very low-quality [48].

*(11) Others*. Only a few pieces of literature focused on TCM symptom efficacy [27, 35, 50] (*P* < 0.05), Lee's Heart Failure Score [53] (*P* < 0.05), hypersensitive C reaction protein (hs-CRP) [54] (*P*=0.06), the ratio of peak mitral valve blood flow velocity in early left ventricular diastole to peak mitral valve blood flow velocity in atrial systole (E/A) [26, 27] (*P* < 0.05), the ratio of peak mitral valve blood flow velocity in early diastole to peak mitral valve annulus velocity in early diastole (E/E′) [27] (*P* < 0.05), left ventricular end-diastolic volume (LVEDV) [28] (*P* < 0.05), left ventricular end-systolic volume (LVESV) [28] (*P* < 0.05), E peak deceleration time (DT) [45] (*P*=0.05), interventricular septal thickness at diastole (IVSd) [48] (*P* < 0.05), and left ventricular posterior wall thickness at diastole (LVPWd) [48] (*P* < 0.05).

#### 3.4.3. Safety Outcome Indicator

*(1) Adverse Events*. Thirty pieces of literature focused on adverse events, including dizziness, dry cough, palpitation, bradycardia, itchy skin, nausea, diarrhea, damage to liver and kidney function, and so on. Five pieces of literature [24, 27, 29, 46, 48] performed a meta-analysis on the incidence of adverse events. Among them, a meta-analysis [24] of 507 CHF patients from 6 RCTs showed that Qili Qiangxin Capsules combined with CT can significantly reduce the incidence of adverse events in CHF patients compared with the control group (*P* < 0.05). The quality of this evidence is low. Besides, another meta-analysis [29] of 4846 HF patients from 56 RCTs showed that Qili Qiangxin Capsules combined with CT can significantly reduce the incidence of adverse events in HF patients compared with the control group (*P* < 0.05). The quality of this evidence is moderate. Three meta-analysis results [27, 46, 48] showed that there are no statistically significant differences between the test group and the control group, all with a very low quality due to study limitations and serious imprecision. Other works of the literature have only conducted a nonquantitative and descriptive analysis of adverse events, and no difference was found between the two groups.

## 4. Discussion

### 4.1. Research Significance and Importance

Current treatment options for HF are diverse, generally including cardiotonic, diuretic, vasodilator, angiotensin-converting enzyme inhibitor (ACEI), angiotensin receptor blocker (ARB), *β*-blocker, and so on. Modern medicine has made great progress in the field of HF, but the prognosis of HF patients is still not satisfactory, resulting in a heavy global burden [57, 58]. Therefore, the development of new therapeutic drugs is an inevitable trend of future medical development. Traditional Chinese medicine mainly comes from pure natural medicine, including botanical medicine, animal medicine, mineral medicine, and so on. Due to the complex active ingredients, Chinese patent medicine has the advantages of the multitarget effect and bidirectional regulation. Therefore, it is receiving more and more attention in the global medical field [59, 60]. Therefore, the development of TCM provides more possibilities for improving the prognosis of HF patients.

### 4.2. Summary of Main Findings

This overview has provided a summary of the efficacy and safety of OCPM combined with CT for HF in 38 eligible systematic reviews of RCTs. Eleven kinds of qualified OCPMs and 24 outcome indicators were included. Among them, LVEF is the most frequently reported outcome indicators, followed by BNP/NT-proBNP, 6-MWT, clinical comprehensive efficacy, and so on.

In the evaluation of efficacy, there was no statistically significant difference between the two groups in terms of mortality, with a low quality. However, compared with the control group, Qili Qiangxin Capsules or Qishen Yiqi Dripping Pills combined with CT can significantly reduce the hospitalization rate of CHF patients, and the quality of the evidence is moderate. In terms of the clinical comprehensive efficacy, in addition to Yixinshu Capsules, the overall efficacy of OCPM combined with CT has been confirmed, and the quality of evidence is at a low to medium level. However, the current research evidence has not yet fully demonstrated the role of Yixinshu Capsules in improving the clinical effectiveness of HF patients, so more clinical research is needed. Besides, the current research has also proved that combining specific OCPM based on CT has a better improvement effect on other secondary efficacy outcome indicators of HF patients than CT alone.

In the evaluation of safety, these adverse events can be observed both in the test group and the control group and there was no significant difference between the two groups, so it has not been proven that the adverse events are related to the use of OCPM. All adverse events are mild and can be tolerated. The results show that the overall role of OCPM is safe.

High-quality evidence does not necessarily indicate strong recommendations, and strong recommendations can also result from low-quality evidence. In addition to the quality of the evidence, several other factors may also influence the strength of the recommendation, for example, the balance between desirable and undesirable effects, value and preferences, and whether the intervention represents a wise use [61]. It needs to be clear that our study did not assess the strength of recommendations.

### 4.3. Limitations and Prospects

#### 4.3.1. Limitations and Prospects of RCTs

Well designed and properly executed RCTs provide the most reliable evidence on the efficacy of healthcare interventions [62], but trials with inadequate methods are associated with bias. Therefore, the standardization process of the clinical trial is the basis for obtaining high-quality evidence. In these included systematic reviews, most RCTs only mentioned the “random” but did not describe the random method in detail, and most RCTs did not mention allocation concealment and blinding. These limitations could cause serious risks of bias. Therefore, it is recommended that researchers strictly design clinical trials according to the CONSORT checklist and describe every item in detail [62]. Systematic reviews based on high-quality RCTs can provide more convincing evidence. Thus it is necessary for systematic reviewers to correctly identify unqualified RCTs and exclude them.

#### 4.3.2. Limitations and Prospects of Systematic Reviews

In this study, apart from one English literature, other systematic reviews failed to provide a protocol or related register information. The lack of an open protocol affected the transparency of the research. Obtaining open registration information in advance can help avoid serious ex-post decision bias in methodology during the systematic review process [63]. Therefore, the system reviewers must register a protocol on the relevant registration platform, such as the international databases PROSPERO (https://www.crd.york.ac.uk/prospero/) before conducting a study.

None of the systematic reviews included provided comprehensive retrieval strategies and the list of excluded studies, which affected the repeatability of the research. In general, a comprehensive literature search strategy mainly includes searching the electronic database, supplemented by searching the reference lists of included studies and trial registries and consulting content experts in the field in addition to relevant gray literature such as degree thesis, conference abstract, etc. Therefore, the research team must include clinical researchers and methodological experts. Strengthening multidisciplinary cooperation will promote the development of TCM. The above information is often not valued by researchers, but it is actually very important. Therefore, systematic reviewers should report all items in related checklists to ensure a complete systematic review is submitted.

Besides, this study shows that the outcome indicators used for clinical efficacy evaluation are mainly examination and laboratory indicators that replace clinical symptoms, such as BNP and LVEF. However, few studies have reported endpoint events that directly reflect the patient's clinical benefit and prognostic, such as mortality and hospitalization rate. On the one hand, because the follow-up time for endpoint events is too long, it is difficult to obtain long-term valid data from patients. On the other hand, the definition of the endpoint events and the time of follow-up are inconsistent, so it is difficult to merge data. Therefore, it is important to standardize the definition of endpoint events in related fields to contribute to the availability of public data and develop a reasonable follow-up schedule to ensure that patients are easily accepted.

#### 4.3.3. Limitations and Prospects of Overview

First, we only searched the relevant Chinese and English databases, so this study has language limitations. Since OCPM is mainly used in China, it can be considered that this bias is not serious. Second, because the definition of adverse events in each RCT is inconsistent, much information about adverse events cannot be effectively used for data synthesis and only descriptive analysis can be performed. Third, some trials have contributed to more than one systematic review. Therefore, the conclusions of this study may be affected to some extent by these overlaps. These limitations need to be improved through more research in the future.

## 5. Conclusion

In summary, the current evidence shows that OCPM has good clinical efficacy and safety in the treatment of HF. However, due to its insufficient methodological quality and evidence quality level, the evidence needs to be further verified. In the future, clinical trials and systematic reviews should strictly follow the guidelines of the Cochrane Collaboration to ensure the standardization of the research process and thus provide more convincing evidence for clinical decision-making.

## Figures and Tables

**Table 1:** Basic characteristics of the included studies.

Study ID	Type of disease	Number of RCTs (sample size)	Treatment group	Control group	Outcomes	Methodological quality evaluation tool
Liu [19]	CHF	7 (403)	QQC (1.2 g, tid) + CT	CT or CT + placebo	①②③④⑤⑥⑦	Cochrane
Shang [20]	CHF	13 (1154)	QQC (0.9–1.2 g, tid) + CT	CT	②③④⑤⑥⑦⑧⑨⑩	Cochrane
Li [21]	CHF	16 (1422)	QQC (NA) + CT	CT	②③④⑤⑥⑧⑩	Cochrane
Zhuang [22]	CHF	57 (4351)	QQC (0.9–1.2 g, tid) + CT	CT	②④⑤⑥⑦⑨⑪⑫⑬	Cochrane
Liu [23]	CHF	79 (7119)	QQC (NA) + CT	CT or CT + placebo	①②③④⑤⑦⑨	Cochrane
Li [24]	CHF	22 (1988)	QQC (1.2 g, tid) + CT	CT or CT + placebo	②③④⑤⑥⑦	Cochrane
Jiang [25]	CHF	17 (1965)	QQC (1.2 g, tid) + CT	CT	①②④⑤⑦⑩	Cochrane
Xu [26]	HFpEF	10 (829)	QQC (NA) + CT	CT or CT + placebo	①②④⑨⑮	Jadad
Feng [27]	DHF	18 (1404)	QQC (NA) + CT	CT	②③⑦⑧⑨⑭⑮⑯	Cochrane
Sun [28]	HF-ICM	22 (1942)	QQC (NA) + CT	CT or CT + placebo	②⑤⑥⑦⑧⑰⑱	Jadad
Sun [29]	HF	120 (10872)	QQC (NA) + CT	CT or CT + placebo	①②⑤⑦⑨⑫⑬	Cochrane
Wang [30]	CHF	17 (1840)	QYDP (0.5 g, tid) + CT	CT	①②④⑤⑦⑧⑫⑬	Cochrane
Liu [31]	CHF	18 (2244)	QYDP (0.5 g, tid) + CT	CT	①②④⑤⑥⑦⑩⑪⑫	Cochrane
Gao [32]	CHF	13 (1541)	QYDP (0.5 g, tid) + CT	CT	①⑦	Jadad
Wang [33]	CHF	22 (2426)	QYDP (0.5 g, tid) + CT	CT	①②④⑤⑥⑧⑪	Jadad
Shan [34]	CHF	8 (788)	QYDP (NA) + CT	CT	⑥⑪	Jadad
Zhang [35]	CHF	11 (931)	QYDP (NA) + CT	CT or CT + placebo	①②④⑤⑦⑨⑭	Cochrane
Chang [36]	CHF	12 (877)	QYDP (0.5 g, tid) + CT	CT	①②④⑤⑥⑦⑪	Jadad
Qu [37]	HF-ICM	10 (1070)	QYDP (0.5 g, tid) + CT	CT	②④⑤⑦⑫	Cochrane
Tian [38]	HF-CHD	15 (1614)	QYDP (0.5 g, tid) + CT	CT	②④⑤⑦⑧	Jadad
An [39]	CHF	22 (1750)	SBP (22.5–45 mg, tid) + CT	CT or CT + placebo	②④⑤⑥⑦⑧	Cochrane
Jin [40]	CHF	31 (2596)	SBP (NA) + CT	CT	②④⑤⑥⑧⑪	Jadad
Dong [41]	CHF	27 (2637)	SBP (22.5–67.5 mg, tid) + CT	CT or CT + placebo	②④⑤⑧⑨⑩⑲	Cochrane
Lin [42]	HF-ICM	12 (1182)	SBP (NA)+CT + trimetazidine	CT + trimetazidine	②⑤⑥⑧	Cochrane
Chen [43]	HF	11 (907)	WK (6 g, tid; 9 g, tid) + CT	CT	④⑤⑦	Cochrane
Liang [44]	HF + Arrhythmia	5 (472)	WK (5 g, tid; 9 g, tid)+CT + amiodarone	CT + amiodarone	⑤⑦⑧	Cochrane
Liu [45]	CHF	10 (1044)	TC (2–4#, tid) + CT	CT	⑤⑥⑦⑧⑫⑬⑳	Jadad
He [46]	HF-CHD	17 (1752)	TC (2–4#, tid) + CT	CT	⑤⑦⑧⑩	Cochrane
Wang [47]	CHF	9 (912)	CDDP (10#, tid) + CT	CT	⑦⑧	Cochrane
Lai [48]	CHF	21 (1691)	CDDP (NA) + CT	CT	②④⑤⑥⑦⑧⑪⑲㉑㉒	Jadad
Wu [49]	CHF	11 (1006)	ZC (NA) + CT	CT	⑤⑦⑧⑨⑩	Cochrane
Cao [50]	CHF	14 (1204)	ZC (0.25∼1 g, tid; 0.5 g, bid) + CT	CT	①④⑤⑦⑩⑭⑲	Cochrane
Chen [51]	HF	13 (1051)	ZC (0.25∼1g, tid) + CT	CT	①⑤⑦	Cochrane
Li [52]	CHF	7 (483)	BQT (4#, tid) + CT	CT	①③④⑤⑧⑨	Cochrane
Mo [53]	CHF	7 (573)	BQT (4#, tid) + CT	CT	①③⑤⑦⑨㉓	Cochrane
Chen [54]	CHF	14 (1404)	YT (0.9–1.2 g, tid) + CT	CT	②④⑤⑦⑧⑨㉔	Jadad
Zhang [55]	CHF	14 (1137)	XZK (0.3 g, bid; 0.6 g, qd; 0.6 g, bid; 0.6 g, tid) + CT	CT or CT + placebo	④⑤⑥⑦⑧	Cochrane
Cai [56]	CHF	19 (2291)	YC (9#∼12#/day) + CT	CT	②⑤⑥⑦	Jadad

① New York Heart Association (NYHA) cardiac function efficacy; ② 6-minute walk test (6-MWT); ③ Minnesota Living with Heart Failure Questionnaire (MLHFQ) score; ④ brain natriuretic peptide (BNP); ⑤ left ventricular ejection fraction (LVEF); ⑥ left ventricular end-diastolic diameter (LVEDD); ⑦ adverse events; ⑧ clinical comprehensive efficacy; ⑨ N-terminal probrain natriuretic peptide (NT-proBNP); ⑩ cardiac output (CO); ⑪ left ventricular end-systolic diameter (LVESD); ⑫ hospitalization rate; ⑬ mortality; ⑭ TCM symptom efficacy; ⑮ the ratio of peak mitral valve blood flow velocity in early left ventricular diastole to peak mitral valve blood flow velocity in atrial systole (E/A); ⑯ the ratio of peak mitral valve blood flow velocity in early diastole to peak mitral valve annulus velocity in early diastole (E/E′); ⑰ left ventricular end-diastolic volume (LVEDV); ⑱ left ventricular end-systolic volume (LVESV); ⑲ stroke volume (SV); ⑳ E peak deceleration time (DT); ㉑ interventricular septal thickness at diastole (IVSd); ㉒ left ventricular posterior wall thickness at diastole (LVPWd); ㉓ Lee's Heart Failure Score; ㉔ hypersensitive C reaction protein (hs-CRP); HF: heart failure; CHF: chronic heart failure; HFpEF: heart failure with preserved ejection fraction; DHF: diastolic heart failure; HF-ICM: heart failure caused by ischemic cardiomyopathy; HF-CHD: heart failure caused by coronary heart disease; NA : Not available; QQC : Qili Qiangxin Capsules; QYDP : Qishen Yiqi Dripping Pills; SBP : Shexiang Baoxin Pills; WK : Wenxin Keli; TC : Tongxinluo Capsules; CDDP : Compound Danshen Dripping Pills; ZC : Zhenyuan Capsules; BQT : Buyi Qiangxin Tablets; YT : Yangxinshi Tablets; XZK : Xuezhikang; YC : Yixinshu Capsules; CT: conventional therapy; Cochrane: Cochrane Reviews' Handbook; Jadad: Jadad Rating Scale.

**Table 2:** Grading of evidence quality of primary efficacy outcome indicators.

Study ID	Participants	Interventions		RCTs (sample size)	Statistics	Grading
		Treatment group	Control group			
Mortality						
Sun [29]	HF	QQC (NA) + CT	CT	6 (539)	RR = 0.53 [0.27, 1.07], *P* > 0.05	Low^a,d^

Hospitalization rate						
Sun [29]	HF	QQC (NA) + CT	CT	9 (669)	RR = 0.49 [0.38, 0.64], *P* < 0.05	Moderate^a^
Wang [30]	CHF	QYDP (0.5 g, tid) + CT	CT	2 (248)	RR = 0.52 [0.33, 0.81], *P*=0.004	Moderate^a^
Liu [31]	CHF	QYDP (0.5 g, tid) + CT	CT	3 (365)	OR = 0.41 [0.23, 0.72], *P*=0.002	Moderate^a^

Clinical comprehensive efficacy						
Shang [20]	CHF	QQC (0.9–1.2 g, tid) + CT	CT	12 (1110)	RR = 1.24 [1.17, 1.31], *P* < 0.00001	Low^a,e^
Li [21]	CHF	QQC (NA) + CT	CT	16 (1422)	RR = 1.18 [1.13, 1.24], *P* < 0.00001	Low^a,e^
Feng [27]	DHF	QQC (NA) + CT	CT	14 (1220)	RR = 1.29 [1.21, 1.36], *P* < 0.00001	Low^a,e^
Sun [28]	HF-ICM	QQC (NA) + CT	CT or CT + placebo	19 (1611)	RR = 1.21 [1.16, 1.27], *P* < 0.00001	Moderate^a^
Wang [30]	CHF	QYDP (0.5 g, tid) + CT	CT	7 (887)	RR = 1.18 [1.12, 1.25], *P* < 0.00001	Moderate^a^
Wang [33]	CHF	QYDP (0.5 g, tid) + CT	CT	16 (1791)	OR = 3.82 [2.83, 5.16], *P* < 0.00001	Low^a,e^
Tian [38]	HF-CHD	QYDP (0.5 g, tid) + CT	CT	12 (1298)	RR = 1.16 [1.11, 1.21], *P* < 0.00001	Low^a,e^
An [39]	CHF	SBP (22.5–45 mg, tid) + CT	CT or CT + placebo	15(1327)	OR = 3.75 [2.72, 5.16], *P* < 0.00001	Low^a,e^
Jin [40]	CHF	SBP (NA) + CT	CT	19 (1560)	RR = 1.18 [1.13, 1.24], *P* < 0.00001	Low^a,e^
Dong [41]	CHF	SBP (22.5–67.5 mg, tid) + CT	CT or CT + placebo	17 (1621)	OR = 3.88 [2.87, 5.26], *P* < 0.00001	Low^a,e^
Lin [42]	HF-ICM	SBP (NA)+CT + trimetazidine	CT + trimetazidine	12 (1186)	RR = 1.30 [1.23, 1.38], *P* < 0.00001	Low^a,e^
Liang [44]	HF + Arrhythmia	WK (5 g, tid; 9 g,tid)+CT + amiodarone	CT + amiodarone	4 (386)	OR = 5.48 [2.59, 11.61], *P* < 0.00001	Moderate^a^
Liu [45]	CHF	TC (2–4#, tid) + CT	CT	10 (915)	OR = 2.76 [1.93, 3.95], *P* < 0.00001	Low^a,e^
He [46]	HF-CHD	TC (2–4#, tid) + CT	CT	16 (1632)	OR = 4.28 [3.04, 6.01], *P* < 0.00001	Moderate^a^
Wang [47]	CHF	CDDP (10#, tid) + CT	CT	9 (912)	RR = 1.22 [1.15, 1.29], *P* < 0.00001	Low^a,e^
Lai [48]	CHF	CDDP (NA) + CT	CT	17 (1440)	RR = 1.21 [1.16, 1.27], *P* < 0.00001	Low^a,e^
Wu [50]	CHF	ZC (NA) + CT	CT	10 (901)	OR = 4.35 [2.97, 6.36], *P* < 0.00001	Low^a,e^
Li [52]	CHF	BQT (4#, tid) + CT	CT	5 (334)	OR = 4.54 [2.23, 9.26], *P* < 0.00001	Moderate^a^
Chen [54]	CHF	YT (0.9–1.2 g, tid) + CT	CT	12 (1232)	OR = 3.24 [2.33, 4.49], *P* < 0.00001	Moderate^a^
Zhang [55]	CHF	XZK (0.3 g, bid; 0.6 g, qd; 0.6 g, bid; 0.6 g, tid) + CT	CT or CT + placebo	5 (382)	OR = 3.04 [1.81, 5.10], *P* < 0.00001	Moderate^a^

HF: heart failure; CHF: chronic heart failure; DHF: diastolic heart failure; HF-ICM: heart failure caused by ischemic cardiomyopathy; HF-CHD: heart failure caused by coronary heart disease; QQC : Qili Qiangxin Capsules; QYDP : Qishen Yiqi Dripping Pills; SBP : Shexiang Baoxin Pills; WK : Wenxin Keli; TC : Tongxinluo Capsules; FDDP : Fufang Danshen Dripping Pills; ZC : Zhenyuan Capsules; BQT : Buyi Qiangxin Tablets; YT : Yangxinshi Tablets; XZK : Xuezhikang; CT: conventional therapy; CI: confidence interval; RR: relative risk; OR: odds ratio. ^a^The limitation (risk of bias) is a factor of downgrading; ^b^The inconsistency is a factor of downgrading; ^c^The indirectness is a factor of downgrading; ^d^The inaccuracy is a factor of downgrading; ^e^The publication bias is a factor of downgrading.

## Data Availability

The data used to support the findings of this study are available from the corresponding author upon request.

## Supplementary Materials

Supplementary Materials Supplementary Material 1: PRIO-harms checklist of items to include when reporting an Overview of Systematic Reviews (OoSRs) *λ*. Supplementary Material 2: search strategy in PubMed *λ*. Supplementary Material 3: methodological quality evaluation of included studies *λ*. Supplementary Material 4: summary of findings (SoFs) tables based on different systematic reviews *λ*. Supplementary Material 5: grading of evidence quality based on different outcome indicators *λ.* Supplementary Material 6: list of excluded literature.

## References

[1] Heart Failure Group of Chinese Society of Cardiology of Chinese Medical Association (2018). Chinese guidelines for the diagnosis and treatment of heart failure 2018. *Chinese Journal of Cardiology*.

[2] Ponikowski P., Voors A. A., Anker S. D., Bueno H. (2016). ESC Guidelines for the diagnosis and treatment of acute and chronic heart failure. *European Heart Journal*.

[3] Chen K. J., Wu Z. G., Zhu M. J., Mao J. Y., Xu H. (2016). Expert consensus on diagnosis and treatment of chronic heart failure with integrated traditional Chinese and western medicine. *Chinese Journal of Integrated Traditional and Western Medicine*.

[4] Wang Y. X., Wu L. J., Li B., Xing Z. Y., Zhu M. J. (2017). Current status analysis on systematic reviews of traditional Chinese medicine in treating chronic heart failure. *China Journal of Traditional Chinese Medicine and Pharmacy*.

[5] Gao H. Y., Zhao Y., Gao R., Li B. (2014). Research status and development methods of Cochrane Overviews: a survey. *Chinese Journal of Evidence-Based Medicine*.

[6] Joanna Briggs Institute (2017). *IOP Publishing JBI Approach to Evidence-Based Healthcare*.

[7] Ji Z. C., Hu H. Y., Li N. (2019). Research progress of overview of traditional Chinese medicine. *China Journal of Chinese Materia Medica*.

[8] Chen H., Fang S. N., Liu J. P., Chen K. J. (2017). Development and status of international evidence-based medicine evidence classification system. *Chinese Journal of Integrated Medicine*.

[9] Pollock M., Fernandes R. M., Becker L. A., Pieper D., Hartling L., Higgins J. P. T., Thomas J., Chandler J. (2020). Chapter V: overviews of reviews. *Cochrane Handbook for Systematic Reviews of Interventions Version 6.0*.

[10] Chinese Pharmacopoeia Committee (2015). *Pharmacopoeia of the People’s Republic of China*.

[11] Shea B. J., Reeves B. C., Wells G. (2017). Amstar 2: a critical appraisal tool for systematic reviews that include randomised or non-randomised studies of healthcare interventions, or both. *BMJ*.

[12] Zhang F. Y., Shen A. M., Zeng X. T., Qiang W. M., Jin Y. H. (2018). An Introduction to AMSTAR 2: a critical appraisal tool for systematic reviews. *Chinese Journal of Evidence-Based Cardiovascular Medicine*.

[13] Guyatt G. H., Oxman A. D., Vist G. (2011). GRADE guidelines: 4. Rating the quality of evidence-study limitations (risk of bias). *Journal of Clinical Epidemiology*.

[14] Guyatt G. H., Oxman A. D., Kunz R. (2011). GRADE guidelines: 7. Rating the quality of evidence-inconsistency. *Journal of Clinical Epidemiology*.

[15] Guyatt G. H., Oxman A. D., Kunz R. (2011). GRADE guidelines: 8. Rating the quality of evidence-indirectness. *Journal of Clinical Epidemiology*.

[16] Guyatt G. H., Oxman A. D., Kunz R. (2011). GRADE guidelines 6. Rating the quality of evidence-imprecision. *Journal of Clinical Epidemiology*.

[17] Guyatt G. H., Oxman A. D., Montori V. (2011). GRADE guidelines: 5. Rating the quality of evidence-publication bias. *Journal of Clinical Epidemiology*.

[18] Balshem H., Helfand M., Schünemann H. J. (2011). GRADE guidelines: 3. Rating the quality of evidence. *Journal of Clinical Epidemiology*.

[19] Liu C. X., Mao J. Y., Wang X. L., Hou Y. Z., Zhang C. (2010). Systematic review for Qili Qiangxin Capsules for chronic heart failure. *Chinese Traditional Patent Medicine*.

[20] Shang Y. D., Zhang J. M., Cui Y., Wu X. R., Fu Y. J. (2013). The meta-analysis of the treatment of chronic heart failure with Qili Qiangxin Capsules. *Anhui Medical and Pharmaceutical Journal*.

[21] Li X. W., Hu Z. R., Luo H. M., Yan X. J., Chen X. K. (2014). A meta-analysis on the curative effect of Qili Qiangxin Capsules on chronic heart failure. *Chinese Journal of Evidence-Based Cardiovascular Medicine*.

[22] Zhuang X. (2015). *Meta-analysis of Clinical Efficacy of Qizhi Qiangxin Capsules Combined with Routine Therapy in Treating Chronic Heart Failure*.

[23] Liu X. H. (2015). *Meta-analysis of the Efficacy of Qiying Qiangxin Capsules in the Treatment of Chronic Heart Failure*.

[24] Li Z. Y., Han T., Li J. H., Cao Q. (2018). Meta-analysis for the efficacy and safety of Qili Qiangxin Capsules in treating the patients with chronic heart failure. *Chinese Circulation Journal*.

[25] Jiang T., Wang W. W., Mei Y. (2015). Meta-analysis of the efficacy of Qili Qiangxin Capsules combined with western medicine to treat chronic heart failure. *Journal of Clinical Cardiology*.

[26] Xu Q., Liu H. J., Liu X. H. (2015). Meta-analysis of the effect of Qili Qiangxin Capsules for patients with heart failure and preserved ejection fraction. *Chinese Journal of Difficult and Complicated Cases*.

[27] Feng Y., Jiang Y. S., He Q. W., Wang Y. G. (2015). Efficacy and safety of Qili Qiangxin Capsules in the treatment of diastolic heart failure: a meta-analysis. *Medical Journal of Wuhan University*.

[28] Sun Y. L., Ruan X. F., Li Y. P., Wang X. L. (2019). Comparative analysis of clinical effects according to syndrome differentiation of Qili Qiangxin Capsules on ischemic heart failure: a meta-analysis. *China Journal of Chinese Materia Medica*.

[29] Sun J., Zhang K., Xiong W.-J. (2016). Clinical effects of a standardized Chinese herbal remedy, Qili Qiangxin, as an adjuvant treatment in heart failure: systematic review and meta-analysis. *BMC Complementary and Alternative Medicine*.

[30] Wang S. H., Mao J. Y., Hou Y. Z., Wang J. Y., Wang X. L., Li Z. J. (2013). Routine western medicine treatment plus Qishen Yiqi Dripping Pills for treating patients with chronic heart failure: a systematic review of randomized control trials. *Chinese Journal of Integrated Traditional and Western Medicine*.

[31] Liu J. G., Gu W. H., Liu X. Q., Li X. S., Huang Q. J. (2014). Meta-analysis of qishenyiqi dripping Pills for chronic heart failure. *Chinese Journal of New Drugs and Clinical Remedies*.

[32] Gao C. C., Xu G. L., Qin L. (2014). Meta-analysis of the efficacy and safety of Qishenyiqi Dripping Pills on chronic congestive heart failure. *Journal of Emergency in Traditional Chinese Medicine*.

[33] Wang Y., Dai X. H. (2015). Meta-analysis of qishen Yiqi dripping Pills in the treatment of chronic heart failure. *Heart Disease Branch of China Association of Chinese Medicine*.

[34] Shan Q. Y., Zhang W., Lv L., Li L. Y., Sun H., Guo Z. X. (2017). Meta-analysis of Qishen Yiqi Dripping Pills in preventing ventricular remodeling in patients with chronic heart failure. *Contemporary Medical Symposium*.

[35] Zhang Y. L., Wang J., Li Y., Zhao H. H., Liu J. J., Wang W. (2019). Meta-analysis for qishen Yiqi dropping Pills in treatment of chronic heart failure with the syndrome of qi deficiency and blood stasis. *Chinese Journal of Experimental Traditional Medical Formulae*.

[36] Chang M., Cheng L., Shen Y., Zhang Y., Zhang Z., Hao P. (2019). Qishenyiqi Dripping Pill improves ventricular remodeling and function in patients with chronic heart failure: a pooled analysis. *Medicine (Baltimore)*.

[37] Qu F., Xing D. M., Zheng W. K., Tian Y., Li Y., Kang L. Y. (2014). Qishen Yiqi Dropping Pills for ischemic heart failure: a systematic review. *Chinese Journal of Experimental Traditional Medical Formulae*.

[38] Tian Y., Gu J. X. (2016). Meta-analysis of Qishen Yiqi Dripping Pills in the treatment of coronary heart disease and heart failure. *Journal of Emergency in Traditional Chinese Medicine*.

[39] An Y. P., Zou X., Yao G. Z. (2015). The efficacy of Shexiang Baoxin Pills in the adjuvant treatment of chronic heart failure: a meta-analysis. *Journal of Traditional Chinese Medicine*.

[40] Jin B., Wu B. W., Zhuang X. Y., Luo X. P., Li Y., Shi H. M. (2015). A meta-analysis of adding Shexiang Baoxin Pills in treating patients with chronic heart failure. *Chinese Journal of Clinical Healthcare*.

[41] Dong T., Wang J., Ma X. (2018). Shexiang Baoxin Pills as an adjuvant treatment for chronic heart failure: a system review and meta-analysis. *Evidence-Based Complementary and Alternative Medicine*.

[42] Lin X. D., Wang J. N., Tang J. M. (2016). Clinical efficacy of Shexiang Baoxin Pills combining trimetazidine in treatment of ischemic cardiomyopathy and heart failure in elderly patients: a meta-analysis. *Chinese Journal of Evidence-Based Cardiovascular Medicine*.

[43] Chen Y., Xiong X., Wang C. (2014). The effects of Wenxin Keli on left ventricular ejection fraction and brain natriuretic peptide in patients with heart failure: a meta-analysis of randomized controlled trials. *Evidence-Based Complementary and Alternative Medicine*.

[44] Liang Y. Y., Yao X. B. (2017). Meta-analysis of Wenxin Keli combined with amiodarone in treating heart failure with arrhythmia. *Chinese Journal of Integrative Medicine on Cardio-/Cerebrovascuiar Disease*.

[45] Liu H. T., Liu X. C., Li M. (2015). A meta-analysis on the effect of Tongxinluo Capsules in treating chronic heart failure. *Chinese Medicine Modern Distance Education of China*.

[46] He X., Lu C. Y., Li J. Q., Jing N., Liu Y. M. (2019). Meta-analysis of Tongxinluo Capsules in treating coronary heart failure. *Chinese Traditional Patent Medicine*.

[47] Wang Q. (2016). *Compound Danshen Dripping Pills Treatment of the Chronic Heart Failure Healing the Meta-Analysis of Curative Effect*.

[48] Lai R. K., Liao L., Pan G. M. (2018). Systematic reviews on curative effect of Compound Danshen Dripping Pills combined with routine western medicine for heart failure. *Journal of New Chinese Medicine*.

[49] Wu C. J., Tang T. S. (2017). Efficacy of Zhenyuan Capsules as adjuvant therapy for chronic heart failure: a meta-analysis. *Chinese Journal of Clinical Pharmacology and Therapeutics*.

[50] Cao Y., Wang W. Q., Lu L., Guo X. M. (2017). Adjuvant effects of Zhenyuan Capsules on chronic heart failure: meta-analysis. *China Journal of Chinese Materia Medica*.

[51] Chen Y. Q., Huang Z. D., Zhang P. F. (2017). A meta-analysis of clinical efficacy and safety of Zhenyuan Capsules on heart failure combined with western medicine. *Journal of Hunan University of Chinese Medicine*.

[52] Li J. T., Lu J., Liu C. Y., Pang Y., Lu J. Q. (2018). Meta-analysis of Buyi Qiangxin Tablets for chronic heart failure. *Journal of New Chinese Medicine*.

[53] Mo X. Y., Wang X. L., Hou Y. Z., Bi Y. F., Mao J. Y. (2018). A meta-analysis on randomized controlled trials of routine western medical treatment plus Buyi Qiangxin Tablets for treating chronic heart failure. *Tianjin Journal of Traditional Chinese Medicine*.

[54] Chen Q. Y., Dai X. H. (2018). Efficacy and safety of Yangxinshi Tablets in the treatment of chronic heart failure: a meta-analysis. *Chinese Journal of Integrative Medicine on Cardio-/Cerebrovascular Disease*.

[55] Zhang H., Zhang T. Q., Gu J. X. (2018). Efficacy of Xuezhikang in the treatment of chronic heart failure: a meta-analysis. *Chinese Journal of Integrative Medicine on Cardio-/Cerebrovascular Disease*.

[56] Cai Y. H., Sun W. P., Li J. Y., Zhang L., Wen J. M., Wu W. (2018). Meta-analysis and trial sequential analysis of Yixinshu Capsules combined with western medicine for chronic heart failure. *Journal of Traditional Chinese Medicine*.

[57] Cook C., Cole G., Asaria P., Jabbour R., Francis D. P. (2014). The annual global economic burden of heart failure. *International Journal of Cardiology*.

[58] Benjamin E. J., Muntner P., Alonso A. (2019). Heart disease and stroke statistics-2019 update: a report from the American Heart Association. *Circulation*.

[59] Bian J., Li Z. (2016). Theory and clinical research progress of dual-direction regulation. *Chinese Journal of Urban and Rural Industrial Hygiene*.

[60] Yu S. Y., Lu Y. (2018). Discussion on action mechanisms of traditional Chinese medicine. *Chinese Journal of Pharmacology and Toxicology*.

[61] Guyatt G. H., Oxman A. D., Vist G. E. (2008). GRADE: an emerging consensus on rating quality of evidence and strength of recommendations. *BMJ*.

[62] Moher D., Hopewell S., Schulz K. F. (2012). CONSORT 2010 explanation and elaboration: updated guidelines for reporting parallel group randomised trials. *International Journal of Surgery*.

[63] Stewart L., Moher D., Shekelle P. (2012). Why prospective registration of systematic reviews makes sense. *Systematic Reviews*.

