# Supplementary material for: Efficacy and Safety of Oral Chinese Patent Medicine Combined with Conventional Therapy for Heart Failure: An Overview of Systematic Reviews
Source: Evid Based Complement Alternat Med. 2020 Aug 27;2020:8620186. doi: 10.1155/2020/8620186 (PMC7474350; doi:10.1155/2020/8620186)
Supplement: Supplementary Materials — Supplementary Material 1: PRIO-harms checklist of items to include when reporting an Overview of Systematic Reviews (OoSRs) λ. Supplementary Material 2: search strategy in PubMed λ. Supplementary Material 3: methodological quality evaluation of included studies λ. Supplementary Material 4: summary of findings (SoFs) tables based on different systematic reviews λ. Supplementary Material 5: grading of evidence quality based on different outcome indicators λ. Supplementary Material 6: list of excluded literature. [file 8620186.f1.pdf]

Supplementary Material 1 - PRIO-harms checklist of items to include when reporting an Overview of Systematic Reviews (OoSRs)

Section/Topic	(Sub-) item #	Checklist item	Reported on page #
**TITLE**			
1. Title	1a	Specify the study design with terms such as "overview of (systematic) reviews", "umbrella review", "(systematic) review of systematic reviews" or "(systematic) meta-review" in the title of the OoSRs.	1
	1b	Mention 'safety' or other related terms, or the adverse event(s) of interest in the title of the OoSRs.	1
**ABSTRACT**			
2. Structured-like summary	2a	Provide a structured-like abstract, as applicable: background, objective, data sources, selection criteria, data extraction, review appraisal, data synthesis methods, results, limitations, conclusions.	2
	2b	Report the main findings of analysis of harms undertaken in the OoSRs or/and in the included SRs.	2
**INTRODUCTION**			
3. Rationale	3a	Specify the rationale and the scope (wide or narrow agendas) for the overview in the context of an existing body of knowledge on the topic.	4
	3b	Provide a balanced presentation of potential benefits and harms of the intervention(s).	4
	3c*	Define which events are considered harms according to previous literature and provide a clear rational for the specific harms included in the OoSRs.	5
4. Objectives (PICOS)	4	Provide an explicit statement of research question(s) that specifies PICOS: •Participants ☑ •Interventions ☑ •Comparators ☑ •Outcomes ☑ •Study design ☑	4-5
**METHODS**			
5. Protocol and registration	5a	Indicate if a protocol exists or not.	No. In fact, we submitted a proposal on the website PROSPERO. However, because the review period is too long, the research has already done before the registration is completed.

Section/Topic	(Sub-) item #	Checklist item	Reported on page #
	5b	If registered, provide the name of the registry (such as a valid Web address, PROSPERO).	No.
6. Eligibility criteria & outcomes of interest	6a	Specify inclusion and exclusion criteria for study design, participants, interventions and comparators in detail.	4-5
	6b	List (and define whenever it is necessary) the outcomes for which data were recorded, ideally include prioritization of main and additional outcomes.	5
	6c	Include adverse events as (primary or secondary) outcome of interest. Define them and grade their severity (such as mild, moderate, severe, fatal), if appropriate.	5
	6d†	Specify report characteristics (such as language restrictions, publication status and years considered) use dascriteria for eligibility for the OoSRs (see also item 7).	5-6
7. Information sources	7a	Search at least two electronic databases.	6
	7b	Search supplementary sources (e.g. hand-searching, reference lists, related reviews and guidelines, protocol registries, conference abstracts and other gray literature).	6
	7c	Report the date of last search and/or dates of coverage for each database.	6
8. Search strategy‡	8a	Specify full electronic search strategy (algorithm) for at least one database including any limits used (e.g. language and date restrictions-see also item 6d and 7c) such that it could be repeated.	5-6; Supplementary Material 2
	8b	Present any additional search process (e.g. algorithm or filter for adverse events, searches in pertinent websites) specifically to identify adverse events that have been investigated.	No.
9. Data management & selection process	9a§	Describe the software that was used to manage records and data throughout the OoSRs.	6
	9b	Define what is a SR and provide the process for selecting SRs and its relevant details (screening the title and abstract or full text by at least two reviewers, selection by multiple independent investigators and resolving disagreements by consensus).	6
	9c	Report any attempt to handle overlapping (include one review among multiple potential candidates by choosing for example the most updated SR, the most methodologically rigorous SR or the SR with larger number of primary studies).	6
10. Additional search for primary studies	10	Report additional search to identify eligible primary studies (e.g. searching in more databases or update the search) and its relevant details.	6
11. Data collection process	11a	Describe the method of data extraction from included SRs (e.g. data collection form, extraction in duplicate and independently, resolving disagreements by consensus).	6

Section/Topic	(Sub-) item #	Checklist item	Reported on page #
	11b	Report any processes for obtaining, confirming or updating data from investigators (e.g. contact with authors of included reviews, obtain data from primary studies of included reviews).	6
12. Data items	12	List (and define whenever is necessary) the variables for which data were recorded (e.g. PICOS items, number of included studies and participants, dose, length of follow up, results, funding sources) and any data assumptions and simplifications made.	6
13. Assessment of methodological quality & quality of evidence	13a	State the evaluation of reporting or/and methodological quality (e.g. using PRISMA or PRISMA-harms, AMSTAR or R-AMSTAR) of the included reviews.	6
	13b‖	State the evaluation of quality for individual studies that are included in the SRs (inform whether tools such as Jadad or RoB of Cochrane were used by the included reviews) and for the additional primary studies.	6
	13c	State the evaluation of quality of evidence (e.g. using GRADE approach).	6-7
	13d	Describe the methods (e.g. piloted forms, independently, in duplicate) used for the quality assessment.	7
14. Meta-bias(es)	14	Specify any planned assessment of meta-bias(es) (such as publication bias or selective reporting across studies, ROBIS tool).	7
15. Data synthesis	15a	Specify clearly the method (narrative, meta-analysis or network meta-analysis) of handling or synthesizing data and their details (e.g. state the principal summary measures that were extracted or calculated, how heterogeneity was assessed, what statistical approaches were employed if a quantitative synthesis has been conducted).	7
	15b	Describe the software that was used to analyze the data if a quantitative synthesis has been conducted.	7
	15c	Report if zero events are included in the studies and how they were handled in statistical analyses, if relevant.	7
	15d	Describe methods of any pre-specified additional analyses (such as sensitivity or subgroup analyses, meta-regression). Describe methods of any pre-specified additional analyses (such as sensitivity or subgroup analyses, meta-regression).	7
**RESULTS**			
16. Review & primary study selection	16a	Provide the details of reviews election (e.g. numbers of reviews screened, retrieved, and included and excluded in the overview) and the number of the additional eligible primary studies that were included, ideally with a flow diagram of the overview process.	8
	16b	Present a flow diagram that gives separately the number of studies focused on harms outcomes.	Available in Table 1.

Section/Topic	(Sub-) item #	Checklist item	Reported on page #
	16c‡	List the studies (full citation) that were excluded after reading the full text and provide reasons.	Supplementary material 6
17. Review & primary study characteristics	17a‡	Describe characteristics of each included SR in tables (such as title or author, search date, PICOS, design and number of studies included, number and age range of participants, dose/frequency, followup period [treatment duration], review limitations, results or conclusion) and of each additional primary study.	10-11
	17b	For each included SR report language and publication status restrictions that have been used.	12
18. Overlapping	18	Present or/and discuss about overlapping of studies within SRs (at least one of the following): •Present measures of overlap (such as CCA). •Provide citation matrix.‡ •Give the number of index publications or/and discuss about overlapping.¶	19<br>Discussion section
19. Present assessment of methodological quality & quality of evidence	19	Present results in text or/and tables‡ of any quality assessment (see also item 13a-c):<br>•Reporting or/and methodological quality of the included SRs.<br>•Inform for the quality of the individual studies that were included in the SRs (report results for sequence generation, allocation concealment, blinding, withdrawals, bias etc.) and for the additional included primary studies.<br>•Quality of evidence.	Supplementary material 4 and Supplementary material 5
20. Present meta-bias(es)	20	Present results of any assessment of meta-bias(es) (such as publication bias or selective reporting across studies, ROBIS assessment).	Supplementary material 4
21. Synthesis of results	21a	Summarize and present the main findings of the overview for benefits and harms. If a quantitative synthesis has been conducted, present each summary measure with a confidence interval, prediction interval or acredible interval and measures of heterogeneity or inconsistency.	13-18; Supplementary material 4 and Supplementary material 5
	21b	Give results of any additional analyses, if done (such as sensitivity, subgroup analyses or meta regression).	Supplementary material 4 and Supplementary material 5
	21c	Report results for adverse events separately for each intervention.	18
**DISCUSSION**			

Section/Topic	(Sub-) item #	Checklist item	Reported on page #
22. Summary of evidence	22	Provide a concise summary of the main findings with the strengths and shortcomings of evidence for each main outcome.	18-19
23. Limitations	23a	Discuss limitations of either the overview or included studies (or both) (e.g. different eligibility criteria, limitations of searching reviews, language restrictions, publication and selection bias).	19-20
	23b	Report possible limitations of the included reviews related to harms (issues of missing data and information, definitions of harms, rare adverse effects).	20
24. Conclusions	24a	Provide a general interpretation of the results in coherence with the review findings and present implications for practice; consider the harms equally as carefully as the benefits and in the context of other evidence.	20
	24b	Present implications for future research.	20
**AUTHORSHIP**			
25. Contributions of authors	25	Provide contributions of authors.	20
26. Dual (co-)authorship	26	Report about dual (co-)authorship in the limitation or declarations of interest section.	21
**FUNDING**			
27. Funding or other support	27a	Indicate sources of financial and other support for the OoSRs (direct funding) or for the authors (indirect funding), or report no funding.	21
	27b	Provide name for the overview funder and/or sponsor, or for the authors' supporters.	21
	27c	Describe roles of funder(s), sponsor(s), and/or institution(s), if any, in conducted the OoSRs.	21

Notes

*Applicable mainly for OoSRs that focus on adverse events. The description could be placed in methods section or in an appendix.

†Language restrictions, publication status and years could also be reported in information sources topic-see item 7.

‡It could also be placed in an appendix as a supplementary material.

§The software used for the management of the records and data could be placed in the data collection process please see item 11.

‖The way of evaluation (e.g.instruments) can be reported in item 19.

¶Index publication is the first occurrence of a primary publication in the included reviews. Discussion for overlapping might be placed in the discussion section.

Abbreviations

OoSRs: Overview of Systematic Reviews; SRs: Systematic Reviews; PICOS: participants, interventions, comparisons, outcomes and study design; CCA: corrected covered area.

Supplementary Material 2 - Search strategy in PubMed

Search	Query
#1	Systemic Review[Publication Type]
#2	Meta Analysis[Publication Type]
#3	Meta-analysis[Publication Type]
#4	#1 or #2 or #3
#5	Heart Failure[MeSH Terms]
#6	heart failure[Title/Abstract]
#7	cardiac failure[Title/Abstract]
#8	heart decompensation[Title/Abstract]
#9	heart dysfunction[Title/Abstract]
#10	cardiac dysfunction[Title/Abstract]
#11	ventricular dysfunction[Title/Abstract]
#12	heart dificiency[Title/Abstract]
#13	cardiac dificiency[Title/Abstract]
#14	heart insufficiency[Title/Abstract]
#15	cardiac insufficiency[Title/Abstract]
#16	#5 or #6 or #7 or #8 or #9 or #10 or #11 or #12 or #13 or #14 or #15
#17	Medicine, Chinese Traditional[MeSH Terms]
#18	Traditional Chinese Medicine[All Fields]
#19	Chinese patent medicine[All Fields]
#20	Chinese patent drug[All Fields]
#21	Chinese proprietary medicine[All Fields]
#22	Chinese proprietary drug[All Fields]
#23	Chinese herbal medicine[All Fields]
#24	Chinese herbal drug[All Fields]
#25	herbal medicine[All Fields]
#26	herbal drug[All Fields]
#27	Complementary Therapies[MeSH Terms]
#28	#17 or #18 or #19 or #20 or #21 or #22 or #23 or #24 or #25 or #26 or #27
#29	#4 and #16 and #28

Supplementary Material 3 - Methodological quality evaluation of included studies

Study ID	Item 1	Item 2*	Item 3	Item 4*	Item 5	Item 6	tem 7*	Item 8	Item 9*	Item 10	Item 11*	Item 12	Item 13*	Item 14	Item 15*	Item 16	Grading
Liu CX 2010	Y	N	N	P	Y	Y	N	P	Y	N	Y	N	Y	Y	N	N	Critically low
Shang YD 2013	Y	N	N	P	Y	Y	N	P	Y	N	Y	N	Y	Y	Y	N	Critically low
Li XW 2014	Y	N	N	P	N	Y	N	N	Y	N	Y	N	Y	Y	Y	N	Critically low
Zhuang X 2015	Y	N	N	P	N	N	N	N	Y	N	N	N	Y	N	N	N	Critically low
Liu XH 2015	Y	N	N	P	Y	N	N	N	Y	N	N	N	Y	N	N	N	Critically low
Li ZY2018	Y	N	N	P	N	N	N	P	Y	N	Y	N	Y	Y	Y	N	Critically low
Jiang T 2015	Y	N	N	P	Y	Y	N	N	Y	N	Y	N	Y	Y	Y	N	Critically low
Xu Q 2015	Y	N	N	P	Y	Y	N	N	N	N	N	N	Y	N	N	N	Critically low
Feng Y 2015	Y	N	N	P	N	Y	N	N	Y	N	N	N	N	N	N	N	Critically low
Sun YL 2019	Y	N	N	P	Y	Y	N	P	Y	N	Y	N	Y	Y	Y	N	Critically low
Sun J.2016	Y	Y	N	P	Y	Y	N	P	Y	N	Y	Y	Y	Y	Y	Y	Critically low
Wang SH 2013	Y	N	N	P	Y	Y	N	P	Y	N	Y	N	Y	N	N	N	Critically low
Liu JG 2014	Y	N	N	P	Y	Y	N	P	Y	N	Y	N	Y	Y	N	N	Critically low
Gao CC 2014	Y	N	N	P	Y	Y	N	P	N	N	N	N	N	N	Y	N	Critically low
Wang Y 2015	Y	N	N	P	Y	Y	N	P	N	N	N	N	N	N	Y	N	Critically low
Shan QY 2017	Y	N	N	P	Y	Y	N	N	Y	N	N	N	Y	N	N	N	Critically low
Zhang YL 2019	Y	N	N	P	Y	Y	N	P	Y	N	Y	N	Y	Y	N	N	Critically low
Chang MZ 2019	Y	N	N	P	Y	Y	N	N	N	N	N	N	Y	N	N	N	Critically low
Qu F 2014	Y	N	N	P	Y	Y	N	P	Y	N	Y	N	Y	Y	N	N	Critically low
Tian Y 2016	Y	N	N	P	Y	Y	N	P	Y	N	Y	Y	Y	Y	N	N	Critically low
An YP 2015	Y	N	N	P	N	Y	N	P	Y	N	N	N	Y	N	Y	N	Critically low
Jin B 2015	Y	N	N	P	N	Y	N	N	N	N	Y	N	Y	Y	Y	N	Critically low
Dong T 2018	Y	N	N	P	Y	Y	N	P	Y	N	Y	N	Y	Y	Y	Y	Critically low
Lin XD 2016	Y	N	N	P	Y	Y	N	P	Y	N	Y	N	Y	Y	Y	N	Critically low
Chen Y 2014	Y	N	N	P	Y	Y	N	P	Y	N	N	N	Y	N	N	Y	Critically low
Liang YY 2017	Y	N	N	P	Y	Y	N	P	Y	N	N	N	Y	N	N	N	Critically low
Liu HT 2015	Y	N	N	P	Y	N	N	P	N	N	Y	N	Y	N	Y	N	Critically low
He X 2019	Y	N	N	P	Y	Y	N	P	Y	N	Y	N	Y	N	Y	N	Critically low
Wang Q 2016	Y	N	N	P	Y	Y	N	P	Y	N	N	N	Y	N	N	N	Critically low

Study ID	Item 1	Item 2*	Item 3	Item 4*	Item 5	Item 6	tem 7*	Item 8	Item 9*	Item 10	Item 11*	Item 12	Item 13*	Item 14	Item 15*	Item 16	Grading
Lai RK 2018	Y	N	N	P	Y	Y	N	N	Y	N	N	N	Y	N	Y	N	Critically low
Wu CJ 2017	Y	N	N	P	Y	N	N	P	Y	N	N	N	Y	N	Y	N	Critically low
Cao Y 2017	Y	N	N	P	Y	Y	N	P	Y	N	Y	N	Y	Y	Y	N	Critically low
Chen YQ 2017	Y	N	N	P	Y	Y	N	P	Y	N	Y	N	Y	Y	Y	N	Critically low
Li JT 2018	Y	N	N	P	Y	Y	N	N	Y	N	Y	N	Y	Y	N	N	Critically low
Mo XY 2018	Y	N	N	P	Y	Y	N	P	Y	N	Y	N	Y	Y	N	N	Critically low
Chen QY 2018	Y	N	N	P	N	Y	N	P	Y	N	N	N	Y	N	Y	N	Critically low
Zhang H 2018	Y	N	N	P	N	N	N	N	Y	N	Y	N	N	Y	N	N	Critically low
Cai YH 2018	Y	N	N	P	Y	Y	N	P	Y	N	Y	N	Y	Y	Y	N	Critically low

* refers to a key item.

Y:Yes; P:Part Yes; N:No.

Item 1: Did the research questions and inclusion criteria for the review include the components of PICO?

Item 2: Did the report of the review contain an explicit statement that the review methods were established prior to the conduct of the review and did the report justify any significant deviations from the protocol?

Item 3: Did the review authors explain their selection of the study designs for inclusion in the review?

Item 4: Did the review authors use a comprehensive literature search strategy?

Item 5: Did the review authors perform study selection in duplicate?

Item 6: Did the review authors perform data extraction in duplicate?

Item 7: Did the review authors provide a list of excluded studies and justify the exclusions?

Item 8: Did the review authors describe the included studies in adequate detail?

Item 9: Did the review authors use a satisfactory technique for assessing the risk of bias (RoB) in individual studies that were included in the review?

Item 10: Did the review authors report on the sources of funding for the studies included in the review?

Item 11: If meta-analysis was performed did the review authors use appropriate methods for statistical combination of results?

Item 12: If meta-analysis was performed, did the review authors assess the potential impact of RoB in individual studies on the results of the meta-analysis or other evidence synthesis?

Item 13: Did the review authors account for RoB in individual studies when interpreting/discussing the results of the review?

Item 14: Did the review authors provide a satisfactory explanation for, and discussion of, any heterogeneity observed in the results of the review?

Item 15: If they performed quantitative synthesis did the review authors carry out an adequate investigation of publication bias (small study bias) and discuss its likely impact on the results of the review?

Item 16: Did the review authors report any potential sources of conflict of interest, including any funding they received for conducting the review?

Supplementary Material 4 - Summary of findings (SoFs) tables

Contents list

[19] Systematic Review for Qili Qiangxin Capsules for Chronic Heart Failure (Author: C.X. Liu, J.Y. Mao, X.L. Wang, Y.Z. Hou, and C. Zhang).. 3
[20] The meta-analysis of the treatment of chronic heart failure with Qili Qiangxin Capsules (Author: Y.D. Shang, J.M. Zhang, Y. Cui, X.R. Wu, and Y.J. Fu)..................... 5
[21] meta-analysis on the curative effect of Qili Qiangxin Capsules on chronic heart failure (Author: X.W. Li, Z.R. Hu, H.M. Luo, X.J. Yan, and X.K. Chen)....................... 8
[22] Meta-analysis of clinical efficacy of Qizhi Qiangxin Capsules combined with routine therapy in treating chronic heart failure (Author: X. Zhuang)...............................11
[23] Meta-analysis of the efficacy of Qiying Qiangxin Capsules in the treatment of chronic heart failure (Author: X.H. Liu)...13
[24] Meta-analysis for the efficacy and safety of Qili Qiangxin Capsules in treating the patients with chronic heart failure (Author: Z.Y. Li, T. Han, J.H. Li, and Q. Cao).... 16
[25] Meta-analysis of the efficacy of Qili Qiangxin Capsules combined with western medicine to treat chronic heart failure (Author: T. Jiang, W.W. Wang, Y. Mei et al.n) 19
[26] Meta-analysis of the effect of Qili Qiangxin Capsules for patients with heart failure and preserved ejection fraction (Author: Q. Xu, H.J. Liu, and X.H. Liu)................21
[27] Efficacy and safety of Qili Qiangxin Capsules in the treatment of diastolic heart failure: a meta-analysis (Author: Y. Feng, Y.S. Jiang, Q.W. He, and Y.G. Wang)....... 23
[28] Comparative analysis of clinical effects according to syndrome differentiation of Qili Qiangxin Capsules on ischemic heart failure: a meta-analysis (Author: Y.L. Sun, X.F. Ruan, Y.P. Li, and X.L. Wang)..26
[29] Clinical effects of a standardized Chinese herbal remedy, Qili Qiangxin, as an adjuvant treatment in heart failure: systematic review and meta-analysis (Author: J. Sun, K. Zhang, W.J. Xiong et al.)..28
[30] Routine western medicine treatment plus Qishen Yiqi Dripping Pills for treating patients with chronic heart failure: a systematic review of randomized control trials (Author: S.H. Wang, J.Y. Mao, Y.Z. Hou, J.Y. Wang, X.L. Wang, and Z.J. Li).. 31
[31] Meta-analysis of Qishenyiqi Dripping Pills for chronic heart failure (Author: J.G. Liu, W.H. Gu, X.Q. Liu, X.S. Li, and Q.J. Huang).. 34
[32] Meta-analysis of the efficacy and safety of Qishenyiqi Dripping Pills on chronic congestive heart failure (Author: C.C. Gao, G.L. Xu, and L. Qin).............................. 36
[33] Meta-analysis of Qishen Yiqi Dripping Pills in the treatment of chronic heart failure (Y. Wang and X.H. Dai).. 37
[34] Meta-analysis of Qishen Yiqi Dripping Pills in preventing ventricular remodeling in patients with chronic heart failure (MQ.Y. Shan, W. Zhang, L. Lv, L.Y. Li, H. Sun,and Z.X. Guo)..40
[35] Meta-analysis for Qishen Yiqi Dropping Pills in treatment of chronic heart failure with the syndrome of Qi Deficiency and Blood Stasis (Author: Y.L. Zhang, J. Wang, Y. Li, H.H. Zhao, J.J. Liu, and W. Wang).. 41
[36] Qishenyiqi Dripping Pill improves ventricular remodeling and function in patients with chronic heart failure: a pooled analysis (M. Chang, L. Cheng, Y. Shen, Y. Zhang, Z. Zhang, and P. Hao).. 43
[37] Qishen Yiqi Dropping Pills for ischemic heart failure: a systematic review (F. Qu, D.M. Xing, W.K. Zheng, Y. Tian, Y. Li, and L.Y. Kang).. 44
[38] Meta-analysis of Qishen Yiqi Dripping Pills in the treatment of coronary heart disease and heart failure (Y. Tian and J.X. Gu)...46
[39] The efficacy of Shexiang Baoxin Pills in the adjuvant treatment of chronic heart failure: a meta-analysis (Author: Y.P. An, X. Zou, G.Z. Yao et al.)............................48
[40] A meta-analysis of adding Shexiang Baoxin Pills in treating patients with chronic heart failure (B. Jin, B.W. Wu, X.Y. Zhuang, X.P. Luo, Y. Li, and H.M. Shi)...........50
[41] Shexiang Baoxin Pills as an adjuvant treatment for chronic heart failure: a system review and meta-analysis (T. Dong, J. Wang, X. Ma et al.)...................................... 53

[42] Clinical efficacy of Shexiang Baoxin Pills combining trimetazidine in treatment of ischemic cardiomyopathy and heart failure in elderly patients: a meta-analysis (X.D. Lin, J.N. Wang, J.M. Tang et al.) 56
[43] The effects of Wenxin Keli on left ventricular ejection fraction and brain natriuretic peptide in patients with heart failure: a meta-analysis of randomized controlled trials (Y. Chen, X. Xiong, C. Wang et al.) 58
[44] Meta-analysis of Wenxin Keli combined with amiodarone in treating heart failure with arrhythmia (Y.Y. Liang and X.B. Yao) 59
[45] A meta-analysis on the effect of Tongxinluo Capsules in treating chronic heart failure (Author: H.T. Liu, X.C. Liu, and M. Li) 60
[46] Meta-analysis of Tongxinluo Capsules in treating coronary heart failure (Author: X. He, C.Y. Lu J.Q., Li, N. Jing, and Y.M. Liu) 63
[47] Compound Danshen Dripping Pills treatment of the chronic heart failure healing the meta-analysis of curative effect (Author: Q. Wang) 65
[48] Systematic reviews on curative effect of Compound Danshen Dripping Pills combined with routine western medicine for heart failure (Author: R.K. Lai, L. Liao, and G.M. Pan) 66
[49] Efficacy of Zhenyuan Capsules as adjuvant therapy for chronic heart failure: a Meta-analysis (Author: C.J. Wu and T.S. Tang) 70
[50] Adjuvant effects of Zhenyuan Capsules on chronic heart failure: Meta-analysis (Author: Y. Cao, W.Q. Wang, L. Lu, and X.M. Guo) 72
[51] A meta-analysis of clinical efficacy and safety of Zhenyuan Capsules on heart failure combined with western medicine (Author: Y.Q. Chen, Z.D. Huang, and P.F. Zhang) 74
[52] Meta-analysis of Buyi Qiangxin Tablets for Chronic Heart Failure (Author: J.T. Li, J. Lu, C.Y. Liu, Y. Pang, and J.Q. Lu) 76
[53] A meta-analysis on randomized controlled trials of routine western medical treatment plus Buyi Qiangxin Tablets for treating chronic heart failure (Author: X.Y. Mo, X.L. Wang, Y.Z. Hou, Y.F. Bi, and J.Y. Mao) 78
[54] Efficacy and safety of Yangxinshi Tablets in the treatment of chronic heart failure: a meta-analysis (Author: Q.Y. Chen and X.H. Dai) 80
[55] Efficacy of Xuezhikang in the treatment of chronic heart failure: a meta-analysis (Author: H. Zhang, T.Q. Zhang, and J.X. Gu) 82
[56] Meta-analysis and trial sequential analysis of Yixinshu Capsules combined with western medicine for chronic heart failure (Author: Y.H. Cai, W.P. Sun, J.Y. Li, L. Zhang, J.M. Wen, and W. Wu) 84

[19] Systematic Review for Qili Qiangxin Capsules for Chronic Heart Failure (Author: C.X. Liu, J.Y. Mao, X.L. Wang, Y.Z. Hou, and C. Zhang)

Patient or population: chronic heart failure (CHF)
Settings: inpatients and outpatient
Intervention: Qili Qiangxin Capsule (1.2g, tid) + CT
Comparison: CT / CT + placebo

**Outcomes**, no of participants (studies)	**Relative effect** (95% CI)	**Anticipated absolute effect* (95% CI)** **Control group** (CT / CT + placebo)	**Test group** (Qili Qiangxin Capsule + CT)	**Difference**	**Quality of the evidence (GRADE)**	**What happens**
**New York Heart Association (NYHA) cardiac function efficacy, 253 (4 studies)**	**RR = 1.21** (1.08 to 1.36)	**95 per 126 (75.4%)**	**116 per 127 (91.3%)** (81.4% to 102.5%)	**15.9% higher** (6.0% to 27.1% higher)	⊕⊕⊕⊖ **moderate due to risk of bias[1]**	Probably to improve NYHA cardiac function efficacy.

**Outcomes**, no of participants (studies)	**Relative effect** (95% CI)	**Anticipated absolute effect (95% CI)** **Control group** ((CT / CT + placebo)	**Test group** (Qili Qiangxin Capsule + CT)	**Difference**	**Quality of the evidence (GRADE)**	**What happens**
**Minnesota Living with Heart Failure Questionnaire (MLHFQ) score, 60 (1 study)**	—	—	—	**8 points lower** (3.48 to 12.52 lower)	⊕⊕⊖⊖ **low due to risk of bias[1] and imprecision[2]**	May decrease MLHFQ score.

**Outcomes**, no of participants (studies)	**Relative effect** (95% CI)	**Anticipated absolute effect (95% CI)** **Control group** (CT / CT + placebo)	**Test group** (Qili Qiangxin Capsule + CT)	**Difference**	**Quality of the evidence (GRADE)**	**What happens**

**6-minute walk test (6-MWT), 233 (4 studies)**	—	—	—	**37.39 meters more** (22.58 to 52.20 more)	⊕⊕⊖⊖ **low due to risk of bias[1] and imprecision[2]**	May increase 6-minute walk test distance.
**Outcomes,** no of participants (studies)	**Relative effect** (95% CI)	**Anticipated absolute effect (95% CI)**			**Quality of the evidence (GRADE)**	**What happens**
		**Control group** (CT / CT + placebo)	**Test group** (Qili Qiangxin Capsule + CT)	**Difference**		
**Brain natriuretic peptide (BNP), 60 (1 study)**	—	—	—	**118.00 pg/mL less** (4.44 to 231.56 less)	⊕⊕⊖⊖ **low due to risk of bias[1] and imprecision[2]**	May decrease the level of BNP.
**Outcomes,** no of participants (studies)	**Relative effect** (95% CI)	**Anticipated absolute effect (95% CI)**			**Quality of the evidence (GRADE)**	**What happens**
		**Control group** (CT / CT + placebo)	**Test group** (Qili Qiangxin Capsule + CT)	**Difference**		
**Left ventricular ejection fraction (LVEF), 260 (4 studies)**	—	—	—	**3.97% more** (2.09% to 5.85% more)	⊕⊕⊖⊖ **low due to risk of bias[1] and imprecision[2]**	May increase LVEF.
**Outcomes,** no of participants (studies)	**Relative effect** (95% CI)	**Anticipated absolute effect (95% CI)**			**Quality of the evidence (GRADE)**	**What happens**
		**Control group** (CT / CT + placebo)	**Test group** (Qili Qiangxin Capsule + CT)	**Difference**		
**Left ventricular end-diastolic diameter (LVEDD), 221 (4 studies)**	—	—	—	**2.81 mm less** (1.41 to 4.22 less)	⊕⊕⊖⊖ **low due to risk of bias[1] and imprecision[2]**	May decrease LVEDD.

Abbreviations: CT, conventional Treatment; CI, confidence interval; RR, risk ratio; GRADE, Grading of Recommendations Assessment, Development and Evaluation.

* The basis for the **risk in the control group** (e.g., the median control group risk across studies) is provided in footnotes. The **risk in the intervention group** (and its 95% confidence interval) is based on the assumed risk in the comparison group and the **relative effect** of the intervention (and its 95% CI).

[1] High risk of bias due to unmentioned allocation concealment and insufficient blind method implementation.

[2] Imprecision due to few events.

[20] The meta-analysis of the treatment of chronic heart failure with Qili Qiangxin Capsules (Author: Y.D. Shang, J.M. Zhang, Y. Cui, X.R. Wu, and Y.J. Fu)

Patient or population: chronic heart failure (CHF)
Settings: inpatients and outpatient
Intervention: Qili Qiangxin Capsule (0.9~1.2g, tid) + CT
Comparison: CT

**Outcomes,** no of participants (studies)	**Relative effect** (95% CI)	**Anticipated absolute effect (95% CI)** **Control group** (CT)	**Test group** (Qili Qiangxin Capsule + CT)	**Difference**	**Quality of the evidence (GRADE)**	**What happens**
**Clinical comprehensive efficacy, 1110 (12 studies)**	**RR = 1.24** (1.17 to1.31)	**401 per 553 (72.5%)**	**501 per 557 (89.9%)** (84.8% to 117.8%)	**17.4% higher** (12.3% to 45.3% higher)	⊕⊕⊖⊖ **low due to risk of bias[1] and publication bias[2]**	May improve clinical comprehensive efficacy.
**Outcomes,** no of participants (studies)	**Relative effect** (95% CI)	**Anticipated absolute effect (95% CI)** **Control group** (CT)	**Test group** (Qili Qiangxin Capsule + CT)	**Difference**	**Quality of the evidence (GRADE)**	**What happens**
**Minnesota Living with Heart Failure Questionnaire (MLHFQ) score, 111 (2 studies)**	—	—	—	**4.20 points lower** (0.36 to 8.05 lower)	⊕⊕⊖⊖ **low due to risk of bias[1] and imprecision[3]**	May decrease MLHFQ score.
**Outcomes,** no of participants (studies)	**Relative effect** (95% CI)	**Anticipated absolute effect (95% CI)** **Control group** (CT)	**Test group** (Qili Qiangxin Capsule + CT)	**Difference**	**Quality of the evidence (GRADE)**	**What happens**

**6-minute walk test (6-MWT), 584 (6 studies)**	—	—	—	**39.39 meters more** (30.61 to 48.16 more)	⊕⊕⊕⊝ **moderate due to risk of bias[1]**	Probably to increase 6-minute walk test distance.
**Outcomes,** no of participants (studies)	**Relative effect** (95% CI)	**Anticipated absolute effect (95% CI)**			**Quality of the evidence (GRADE)**	**What happens**
		**Control group** (CT)	**Test group** (Qili Qiangxin Capsule + CT)	**Difference**		
**Brain natriuretic peptide (BNP), 401 (4 studies)**	—	—	—	**113.78 pg/mL less** (32.82 to 194.75 less)	⊕⊕⊝⊝ **low due to risk of bias[1] and inconsistency[4]**	May decrease the level of BNP.
**Outcomes,** no of participants (studies)	**Relative effect** (95% CI)	**Anticipated absolute effect (95% CI)**			**Quality of the evidence (GRADE)**	**What happens**
		**Control group** (CT)	**Test group** (Qili Qiangxin Capsule + CT)	**Difference**		
**N-terminal pro-brain natriuretic peptide (NT-proBNP), 242 (3 studies)**	—	—	—	**90.21 pg/mL less** (60.07 to 120.35 less)	⊕⊕⊝⊝ **low due to risk of bias[1] and imprecision[3]**	May decrease the level of NT-proBNP.
**Outcomes,** no of participants (studies)	**Relative effect** (95% CI)	**Anticipated absolute effect (95% CI)**			**Quality of the evidence (GRADE)**	**What happens**
		**Control group** (CT)	**Test group** (Qili Qiangxin Capsule + CT)	**Difference**		
**Left ventricular ejection fraction (LVEF), 981 (10 studies)**	—	—	—	**6.04% more** (4.57% to 7.52% more)	⊕⊕⊝⊝ **low due to risk of bias[1] and inconsistency[5]**	May increase LVEF.
**Outcomes,** no of participants (studies)	**Relative effect** (95% CI)	**Anticipated absolute effect (95% CI)**			**Quality of the evidence (GRADE)**	**What happens**
		**Control group** (CT)	**Test group** (Qili Qiangxin Capsule +	**Difference**		

			CT)			
**Left ventricular end-diastolic diameter (LVEDD), 340 (3 studies)**	—	—	—	**4.28 mm less** (2.14 to 6.41 less)	⊕⊖⊖⊖ **very low due to risk of bias[1], imprecision[3] and inconsistency[6]**	Uncertain to decrease LVEDD.
**Outcomes,** no of participants (studies)	**Relative effect** (95% CI)	**Anticipated absolute effect (95% CI)** **Control group** (CT)	**Test group** (Qili Qiangxin Capsule + CT)	**Difference**	**Quality of the evidence (GRADE)**	**What happens**
**Cardiac output (CO), 240 (3 studies)**	—	—	—	**0.54 L/min more** (0.05 to 1.02 more)	⊕⊖⊖⊖ **very low due to risk of bias[1], imprecision[3] and inconsistency[7]**	Uncertain to increase CO.

Abbreviations: CT, conventional treatment; CI, confidence interval; RR, risk ratio; GRADE, Grading of Recommendations Assessment, Development and Evaluation.

***** The basis for the **risk in the control group** (e.g., the median control group risk across studies) is provided in footnotes. The **risk in the intervention group** (and its 95% confidence interval) is based on the assumed risk in the comparison group and the **relative effect** of the intervention (and its 95% CI).

[1] High risk of bias due to unmentioned allocation concealment and insufficient blind method implementation.

[2] The funnel chart is asymmetric, suggesting publication bias.

[3] Imprecision due to few events.

[4] Serious unexplained inconsistency (large heterogeneity I^2 = 78%, *P*-value (P = 0.003), point estimates, and confidence intervals vary considerably).

[5] Serious unexplained inconsistency (large heterogeneity I^2 = 80%, *P*-value (P < 0.00001), point estimates, and confidence intervals vary considerably).

[6] Serious unexplained inconsistency (large heterogeneity I^2 = 62%, *P*-value (P = 0.07), point estimates, and confidence intervals vary considerably).

[7] Serious unexplained inconsistency (large heterogeneity I^2 = 92%, *P*-value (P < 0.00001), point estimates, and confidence intervals vary considerably).

[21] meta-analysis on the curative effect of Qili Qiangxin Capsules on chronic heart failure (Author: X.W. Li, Z.R. Hu, H.M. Luo, X.J. Yan, and X.K. Chen)

Patient or population: chronic heart failure (CHF)
Settings: inpatients and outpatient
Intervention: Qili Qiangxin Capsule + CT
Comparison: CT

**Outcomes**, no of participants (studies)	**Relative effect** (95% CI)	**Anticipated absolute effect (95% CI)** Control group (CT)	Test group (Qili Qiangxin Capsule + CT)	Difference	**Quality of the evidence (GRADE)**	**What happens**
**Clinical comprehensive efficacy, 1422 (16 studies)**	**RR = 1.18** (1.13 to 1.24)	**545 per 699 (78.0%)**	**668 per 723 (92.4%)** (88.1% to 96.7%)	**14.4% higher** (10.1% to 18.7% higher)	⊕⊕⊖⊖ **low due to risk of bias[1] and publication bias[2]**	May improve clinical comprehensive efficacy.
**Outcomes**, no of participants (studies)	**Relative effect** (95% CI)	**Anticipated absolute effect (95% CI)** Control group (CT)	**Test group** (Qili Qiangxin Capsule + CT)	**Difference**	**Quality of the evidence (GRADE)**	**What happens**
**Minnesota Living with Heart Failure Questionnaire (MLHFQ) score, 134 (2 studies)**	—	—	—	**7.69 points lower** (4.39 to 10.99 lower)	⊕⊕⊖⊖ **low due to risk of bias[1] and imprecision[3]**	May decrease MLHFQ score.
**Outcomes**, no of participants (studies)	**Relative effect** (95% CI)	**Anticipated absolute effect (95% CI)** Control group (CT)	**Test group** (Qili Qiangxin Capsule + CT)	**Difference**	**Quality of the evidence (GRADE)**	**What happens**

**6-minute walk test (6-MWT), 542 (6 studies)**	—	—	—	**41.44 meters more** (33.30 to 49.59 more)	⊕⊕⊕⊖ **moderate due to risk of bias[1]**	Probably to increase 6-minute walk test distance.
**Outcomes,** no of participants (studies)	**Relative effect** (95% CI)	**Anticipated absolute effect (95% CI)** **Control group** (CT)	**Test group** (Qili Qiangxin Capsule + CT)	**Difference**	**Quality of the evidence (GRADE)**	**What happens**
**Brain natriuretic peptide (BNP), 444 (5 studies)**	—	—	—	**95.86 pg/mL less** (43.96 to 147.77 less)	⊕⊕⊖⊖ **low due to risk of bias[1] and inconsistency[4]**	May decrease the level of BNP.
**Outcomes,** no of participants (studies)	**Relative effect** (95% CI)	**Anticipated absolute effect (95% CI)** **Control group** (CT)	**Test group** (Qili Qiangxin Capsule + CT)	**Difference**	**Quality of the evidence (GRADE)**	**What happens**
**N-terminal pro-brain natriuretic peptide (NT-proBNP), 242 (3 studies)**	—	—	—	**90.21 pg/mL less** (60.07 to 120.35 less)	⊕⊕⊖⊖ **low due to risk of bias[1] and imprecision[3]**	May decrease the level of NT-proBNP.
**Outcomes,** no of participants (studies)	**Relative effect** (95% CI)	**Anticipated absolute effect (95% CI)** **Control group** (CT)	**Test group** (Qili Qiangxin Capsule + CT)	**Difference**	**Quality of the evidence (GRADE)**	**What happens**
**Left ventricular ejection fraction (LVEF), 1204 (13 studies)**	—	—	—	**4.89% more** (3.03% to 6.75% more)	⊕⊕⊖⊖ **low due to risk of bias[1] and inconsistency[5]**	May increase LVEF.
**Outcomes,** no of participants (studies)	**Relative effect** (95% CI)	**Anticipated absolute effect (95% CI)** **Control group** (CT)	**Test group** (Qili Qiangxin Capsule +	**Difference**	**Quality of the evidence (GRADE)**	**What happens**

			CT)			
**Left ventricular end-diastolic diameter (LVEDD), 625 (6 studies)**	—	—	—	**3.22 mm less** (1.64 to 4.80 less)	⊕⊕⊖⊖ **low due to risk of bias[1] and inconsistency[6]**	May decrease LVEDD.
**Outcomes,** no of participants (studies)	**Relative effect** (95% CI)	**Anticipated absolute effect (95% CI)**			**Quality of the evidence (GRADE)**	**What happens**
		**Control group** (CT)	**Test group** (Qili Qiangxin Capsule + CT)	**Difference**		
**Cardiac output (CO), 460 (5 studies)**	—	—	—	**0.22 L/min more** (0.00 to 0.44 more)	⊕⊖⊖⊖ **very low due to risk of bias[1], inconsistency[7] and imprecision[8]**	Uncertain to increase CO.

Abbreviations: CT, conventional treatment; CI, confidence interval; RR, risk ratio; GRADE, Grading of Recommendations Assessment, Development and Evaluation.

***** The basis for the **risk in the control group** (e.g., the median control group risk across studies) is provided in footnotes. The **risk in the intervention group** (and its 95% confidence interval) is based on the assumed risk in the comparison group and the **relative effect** of the intervention (and its 95% CI).

[1] High risk of bias due to unmentioned allocation concealment and insufficient blind method implementation.

[2] The funnel chart is asymmetric, suggesting publication bias.

[3] Imprecision due to few events.

[4] Serious unexplained inconsistency (large heterogeneity I^2 = 70%, P-value (P = 0.01), point estimates, and confidence intervals vary considerably).

[5] Serious unexplained inconsistency (large heterogeneity I^2 = 92%, P-value (P < 0.00001), point estimates, and confidence intervals vary considerably).

[6] Serious unexplained inconsistency (large heterogeneity I^2 = 78%, P-value (P = 0.0003), point estimates, and confidence intervals vary considerably).

[7] Serious unexplained inconsistency (large heterogeneity I^2 = 83%, P-value (P = 0.0001), point estimates, and confidence intervals vary considerably).

[8] Imprecision due to the confidence interval crossing the invalid line zero.

[22] Meta-analysis of clinical efficacy of Qizhi Qiangxin Capsules combined with routine therapy in treating chronic heart failure (Author: X. Zhuang)

Patient or population: chronic heart failure (CHF)

Settings: inpatients and outpatient

Intervention: Qili Qiangxin Capsule (0.9~1.2g, tid) + CT

Comparison: CT

**Outcomes,** no of participants (studies)	**Relative effect** (95% CI)	**Anticipated absolute effect (95% CI)**			**Quality of the evidence (GRADE)**	**What happens**
		**Control group** (CT)	**Test group** (Qili Qiangxin Capsule + CT)	**Difference**		
**Brain natriuretic peptide (BNP), 842 (9 studies)**	**SMD = -0.77 (-0.91 to -0.63)**	—	—	—	⊕⊕⊖⊖ **low due to risk of bias[1] and inconsistency[2]**	May decrease the level of BNP.

**Outcomes,** no of participants (studies)	**Relative effect** (95% CI)	**Anticipated absolute effect (95% CI)**			**Quality of the evidence (GRADE)**	**What happens**
		**Control group** (CT)	**Test group** (Qili Qiangxin Capsule + CT)	**Difference**		
**N-terminal pro-brain natriuretic peptide (NT-proBNP), 430 (5 studies)**	**SMD = -0.38 (-0.57 to -0.19)**	—	—	—	⊕⊕⊖⊖ **low due to risk of bias[1] and inconsistency[2]**	May decrease the level of NT-proBNP.

**Outcomes,** no of participants (studies)	**Relative effect** (95% CI)	**Anticipated absolute effect (95% CI)**			**Quality of the evidence (GRADE)**	**What happens**
		**Control group** (CT)	**Test group** (Qili Qiangxin Capsule + CT)	**Difference**		
**Left ventricular ejection fraction (LVEF), 2570 (29**	**SMD = 0.48 (0.40 to 0.56)**	—	—	—	⊕⊖⊖⊖ **very low due to risk of bias[1], inconsistency[2] and**	Uncrtain to increase LVEF.

**studies)**					**publication bias[3]**	

**Outcomes,** no of participants (studies)	**Relative effect** (95% CI)	**Anticipated absolute effect (95% CI)**			**Quality of the evidence (GRADE)**	**What happens**
		**Control group** (CT)	**Test group** (Qili Qiangxin Capsule + CT)	**Difference**		
**Left ventricular end-diastolic diameter (LVEDD), 1192 (13 studies)**	**SMD = -0.40 (-0.51 to -0.28)**	—	—	—	⊕⊖⊖⊖ **very low due to risk of bias[1], inconsistency[2] and publication bias[3]**	Uncertain to decrease LVEDD.

**Outcomes,** no of participants (studies)	**Relative effect** (95% CI)	**Anticipated absolute effect (95% CI)**			**Quality of the evidence (GRADE)**	**What happens**
		**Control group** (CT)	**Test group** (Qili Qiangxin Capsule + CT)	**Difference**		
**Left ventricular end-systolic diameter (LVESD), 276 (3 studies)**	**SMD = -0.90 (-1.15 to -0.65)**	—	—	—	⊕⊖⊖⊖ **very low due to risk of bias[1], inconsistency[2] and imprecision[4]**	Uncertain to decrease LVESD.

Abbreviations: CT, conventional treatment; CI, confidence interval; SMD, standardized mean difference; GRADE, Grading of Recommendations Assessment, Development and Evaluation.

***** The basis for the **risk in the control group** (e.g., the median control group risk across studies) is provided in footnotes. The **risk in the intervention group** (and its 95% confidence interval) is based on the assumed risk in the comparison group and the **relative effect** of the intervention (and its 95% CI).

[1] High risk of bias due to unmentioned allocation concealment and insufficient blind method implementation.

[2] Unexplained inconsistency.

[3] The funnel chart is asymmetric, suggesting publication bias.

[4] Imprecision due to few events.

[23] Meta-analysis of the efficacy of Qiying Qiangxin Capsules in the treatment of chronic heart failure (Author: X.H. Liu)

Patient or population: chronic heart failure (CHF)

Settings: inpatients and outpatient

Intervention: Qili Qiangxin Capsule + CT

Comparison: CT / CT + placebo

**Outcomes,** no of participants (studies)	**Relative effect** (95% CI)	**Anticipated absolute effect* (95% CI)** **Control group** (CT / CT + placebo)	**Test group** (Qili Qiangxin Capsule + CT)	**Difference**	**Quality of the evidence (GRADE)**	**What happens**
**New York Heart Association (NYHA) cardiac function efficacy, 4510 (51 studies)**	**RR = 1.23** (1.19 to 1.26)	—	—	—	⊕⊕⊖⊖ **low due to risk of bias[1] and publication bias[2]**	May improve NYHA cardiac function efficacy.

**Outcomes,** no of participants (studies)	**Relative effect** (95% CI)	**Anticipated absolute effect (95% CI)** **Control group** (CT / CT + placebo)	**Test group** (Qili Qiangxin Capsule + CT)	**Difference**	**Quality of the evidence (GRADE)**	**What happens**
**Minnesota Living with Heart Failure Questionnaire (MLHFQ) score, 562 (8 studies)**	—	—	—	**6.42 points lower** (5.26 to 7.58 lower)	⊕⊕⊖⊖ **low due to risk of bias[1] and publication bias[2]**	May decrease MLHFQ score.

**Outcomes,** no of participants (studies)	**Relative effect** (95% CI)	**Anticipated absolute effect (95% CI)** **Control group** (CT / CT + placebo)	**Test group** (Qili Qiangxin Capsule + CT)	**Difference**	**Quality of the evidence (GRADE)**	**What happens**

**6-minute walk test (6-MWT), 3654 (38 studies)**	—	—	—	**48.52 meters more** (40.15 to 56.88 more)	⊕⊕⊕⊝ **moderate due to risk of bias[1]**	Probably to increase 6-minute walk test distance.
**Outcomes,** no of participants (studies)	**Relative effect** (95% CI)	**Anticipated absolute effect (95% CI)**			**Quality of the evidence (GRADE)**	**What happens**
		**Control group** (CT / CT + placebo)	**Test group** (Qili Qiangxin Capsule + CT)	**Difference**		
**Brain natriuretic peptide (BNP), 959 (13 studies)**	—	—	—	**86.64 pg/mL less** (53.15 to 120.13 less)	⊕⊝⊝⊝ **very low due to risk of bias[1], publication bias[2] and inconsistency[3]**	Uncertain to decrease the level of BNP.
**Outcomes,** no of participants (studies)	**Relative effect** (95% CI)	**Anticipated absolute effect (95% CI)**			**Quality of the evidence (GRADE)**	**What happens**
		**Control group** (CT / CT + placebo)	**Test group** (Qili Qiangxin Capsule + CT)	**Difference**		
**N-terminal pro-brain natriuretic peptide (NT-proBNP), 1666 (8 studies)**	—	—	—	**236.46 pg/mL less** (64.81 to 308.12 less)	⊕⊕⊝⊝ **low due to risk of bias[1] and imprecision[4]**	May decrease the level of NT-proBNP.
**Outcomes,** no of participants (studies)	**Relative effect** (95% CI)	**Anticipated absolute effect (95% CI)**			**Quality of the evidence (GRADE)**	**What happens**
		**Control group** (CT / CT + placebo)	**Test group** (Qili Qiangxin Capsule + CT)	**Difference**		
**Left ventricular ejection fraction (LVEF), 5361 (57 studies)**	—	—	—	**5.16% more** (4.24% to 6.08% more)	⊕⊕⊝⊝ **low due to risk of bias[1] and inconsistency[5]**	May increase LVEF.

Abbreviations: CT, conventional treatment; CI, confidence interval; RR, risk ratio; GRADE, Grading of Recommendations Assessment, Development and Evaluation.

***** The basis for the **risk in the control group** (e.g., the median control group risk across studies) is provided in footnotes. The **risk in the intervention group** (and its 95% confidence interval) is based on the assumed risk in the comparison group and the **relative effect** of the intervention (and its 95% CI).

[1] High risk of bias due to unmentioned allocation concealment and insufficient blind method implementation.

[2] The funnel chart is asymmetric, suggesting publication bias.

[3] Serious unexplained inconsistency (large heterogeneity I^2 = 90%, P-value ($P < 0.001$), point estimates, and confidence intervals vary considerably).

[4] Serious unexplained inconsistency (large heterogeneity I^2 = 97%, P-value ($P < 0.001$), point estimates, and confidence intervals vary considerably).

[5] Serious unexplained inconsistency (large heterogeneity I^2 = 93%, P-value ($P < 0.001$), point estimates, and confidence intervals vary considerably).

[24] Meta-analysis for the efficacy and safety of Qili Qiangxin Capsules in treating the patients with chronic heart failure

(Author: Z.Y. Li, T. Han, J.H. Li, and Q. Cao)

Patient or population: chronic heart failure (CHF)

Settings: inpatients and outpatient

Intervention: Qili Qiangxin Capsule (1.2g, tid) + CT

Comparison: CT / CT + placebo

**Outcomes,** no of participants (studies)	**Relative effect** (95% CI)	**Anticipated absolute effect (95% CI)**			**Quality of the evidence (GRADE)**	**What happens**
		**Control group** (CT / CT + placebo)	**Test group** (Qili Qiangxin Capsule + CT)	**Difference**		
**Minnesota Living with Heart Failure Questionnaire (MLHFQ) score, 200 (2 studies)**	—	—	—	**8.11 points lower** (6.0 to 10.23 lower)	⊕⊖⊖⊖ **very low due to risk of bias[1], imprecision[2] and inconsistency[3]**	Uncertain to decrease MLHFQ score.
**Outcomes,** no of participants (studies)	**Relative effect** (95% CI)	**Anticipated absolute effect (95% CI)**			**Quality of the evidence (GRADE)**	**What happens**
		**Control group** (CT / CT + placebo)	**Test group** (Qili Qiangxin Capsule + CT)	**Difference**		
**6-minute walk test (6-MWT), 690 (8 studies)**	—	—	—	**53.81 meters more** (46.9 to 60.73 more)	⊕⊕⊕⊖ **moderate due to risk of bias[1]**	Probably to increase 6-minute walk test distance.
**Outcomes,** no of participants (studies)	**Relative effect** (95% CI)	**Anticipated absolute effect (95% CI)**			**Quality of the evidence (GRADE)**	**What happens**
		**Control group** (CT / CT + placebo)	**Test group** (Qili Qiangxin Capsule + CT)	**Difference**		

**Brain natriuretic peptide (BNP), 1020 (10 studies)**	—	—	—	**194.97 pg/mL less** (101.99 to 287.95 less)	⊕⊕⊖⊖ **low due to risk of bias[1] and inconsistency[4]**	May decrease the level of BNP.
**Outcomes,** no of participants (studies)	**Relative effect** (95% CI)	**Anticipated absolute effect (95% CI)** **Control group** (CT / CT + placebo)	**Test group** (Qili Qiangxin Capsule + CT)	**Difference**	**Quality of the evidence (GRADE)**	**What happens**
**Left ventricular ejection fraction (LVEF), 1580 (17 studies)**	—	—	—	**5.24% more** (3.38% to 7.11% more)	⊕⊖⊖⊖ **very low due to risk of bias[1], inconsistency[5] and publication bias[6]**	Uncertain to increase LVEF.
**Outcomes,** no of participants (studies)	**Relative effect** (95% CI)	**Anticipated absolute effect (95% CI)** **Control group** (CT / CT + placebo)	**Test group** (Qili Qiangxin Capsule + CT)	**Difference**	**Quality of the evidence (GRADE)**	**What happens**
**Left ventricular end-diastolic diameter (LVEDD), 921 (10 studies)**	—	—	—	**0.94 mm less** (0.43 to 1.46 less)	⊕⊕⊖⊖ **low due to risk of bias[1] and inconsistency[7]**	May decrease LVEDD.
**Outcomes,** no of participants (studies)	**Relative effect** (95% CI)	**Anticipated absolute effect (95% CI)** **Control group** (CT / CT + placebo)	**Test group** (Qili Qiangxin Capsule + CT)	**Difference**	**Quality of the evidence (GRADE)**	**What happens**
**Adverse events, 507 (6 studies)**	**OR = 0.44** (0.25 to 0.79)	**15.3%**	**7.51%**	7.79% lower	⊕⊕⊖⊖ **low due to risk of bias[1] and imprecision[2]**	Qili Qiangxin combined with conventional treatment may significantly reduce risk of adverse events.

Abbreviations: CT, conventional treatment; CI, confidence interval; GRADE, Grading of Recommendations Assessment, Development and Evaluation.

* The basis for the **risk in the control group** (e.g., the median control group risk across studies) is provided in footnotes. The **risk in the intervention group** (and its 95% confidence interval) is based on the assumed risk in the comparison group and the **relative effect** of the intervention (and its 95% CI).

[1] High risk of bias due to unmentioned allocation concealment and insufficient blind method implementation.

[2] Imprecision due to few events.

[3] Serious unexplained inconsistency (large heterogeneity I^2 = 69%, *P*-value (P = 0.07), point estimates, and confidence intervals vary considerably).

[4] Serious unexplained inconsistency (large heterogeneity I^2 = 99%, *P*-value (P < 0.00001), point estimates, and confidence intervals vary considerably).

[5] Serious unexplained inconsistency (large heterogeneity I^2 = 94%, *P*-value (P < 0.00001), point estimates, and confidence intervals vary considerably).

[6] The funnel chart is asymmetric, suggesting publication bias.

[7] Serious unexplained inconsistency (large heterogeneity I^2 = 92%, *P*-value (P < 0.00001), point estimates, and confidence intervals vary considerably).

[25] Meta-analysis of the efficacy of Qili Qiangxin Capsules combined with western medicine to treat chronic heart failure (Author: T. Jiang, W.W. Wang, Y. Mei et al.n)

Patient or population: chronic heart failure (CHF)
Settings: inpatients and outpatient
Intervention: Qili Qiangxin Capsule (1.2g, tid) + CT
Comparison: CT

**Outcomes,** no of participants (studies)	**Relative effect** (95% CI)	**Anticipated absolute effect* (95% CI)** Control group (CT)	Test group (Qili Qiangxin Capsule + CT)	Difference	**Quality of the evidence (GRADE)**	**What happens**
**New York Heart Association (NYHA) cardiac function efficacy, 1237 (16 studies)**	**OR = 3.83** (2.78 to 5.28)	**554 per 731 (75.4%)**	**683 per 742 (91.3%)**	**15.9% higher**	⊕⊕⊖⊖ **low due to risk of bias[1] and publication bias[2]**	May improve NYHA cardiac function efficacy.

**Outcomes,** no of participants (studies)	**Relative effect** (95% CI)	**Anticipated absolute effect (95% CI)** Control group (CT)	Test group (Qili Qiangxin Capsule + CT)	Difference	**Quality of the evidence (GRADE)**	**What happens**
**6-minute walk test (6-MWT), 994 (6 studies)**	—	—	—	**34.10 meters more** (25.88 to 42.32 more)	⊕⊕⊕⊖ **moderate due to risk of bias[1]**	Probably to increase 6-minute walk test distance.

**Outcomes,** no of participants (studies)	**Relative effect** (95% CI)	**Anticipated absolute effect (95% CI)** Control group (CT)	Test group (Qili Qiangxin Capsule + CT)	Difference	**Quality of the evidence (GRADE)**	**What happens**

**Brain natriuretic peptide (BNP), 791 (8 studies)**	—	—	—	**71.69 pg/mL less** (45.16 to 98.21 less)	⊕⊕⊖⊖ **low due to risk of bias[1] and inconsistency[3]**	May decrease the level of BNP.
**Outcomes,** no of participants (studies)	**Relative effect** (95% CI)	**Anticipated absolute effect (95% CI)** **Control group** (CT)	**Test group** (Qili Qiangxin Capsule + CT)	**Difference**	**Quality of the evidence (GRADE)**	**What happens**
**Left ventricular ejection fraction (LVEF), 1884 (16 studies)**	—	—	—	**6.32% more** (3.92% to 8.73% more)	⊕⊖⊖⊖ **very low due to risk of bias[1], publication bias[2] and inconsistency[4]**	Uncertain to increase LVEF.
**Outcomes,** no of participants (studies)	**Relative effect** (95% CI)	**Anticipated absolute effect (95% CI)** **Control group** (CT)	**Test group** (Qili Qiangxin Capsule + CT)	**Difference**	**Quality of the evidence (GRADE)**	**What happens**
**Cardiac output (CO), 499 (5 studies)**	—	—	—	**0.48 L/min more** (0.03 to 0.93 more)	⊕⊕⊖⊖ **low due to risk of bias[1] and inconsistency[5]**	May increase CO.

Abbreviations: CT, conventional treatment; CI, confidence interval; OR, odds ratio; GRADE, Grading of Recommendations Assessment, Development and Evaluation.

* The basis for the **risk in the control group** (e.g., the median control group risk across studies) is provided in footnotes. The **risk in the intervention group** (and its 95% confidence interval) is based on the assumed risk in the comparison group and the **relative effect** of the intervention (and its 95% CI).

[1] High risk of bias due to unmentioned allocation concealment and insufficient blind method implementation.

[2] The funnel chart is asymmetric, suggesting publication bias.

[3] Serious unexplained inconsistency (large heterogeneity I^2 = 65%, P-value (P = 0.003), point estimates, and confidence intervals vary considerably).

[4] Serious unexplained inconsistency (large heterogeneity I^2 = 94%, P-value (P < 0.00001), point estimates, and confidence intervals vary considerably).

[5] Serious unexplained inconsistency (large heterogeneity I^2 = 79%, P-value (P = 0.0008), point estimates, and confidence intervals vary considerably).

[26] Meta-analysis of the effect of Qili Qiangxin Capsules for patients with heart failure and preserved ejection fraction (Author: Q. Xu, H.J. Liu, and X.H. Liu)

Patient or population: heart failure with preserved ejection fraction (HFpEF)
Settings: inpatients and outpatient
Intervention: Qili Qiangxin Capsule + CT
Comparison: CT / CT + placebo

**Outcomes,** no of participants (studies)	**Relative effect** (95% CI)	**Anticipated absolute effect* (95% CI)** **Control group** (CT / CT + placebo)	**Test group** (Qili Qiangxin Capsule + CT)	**Difference**	**Quality of the evidence (GRADE)**	**What happens**
**New York Heart Association (NYHA) cardiac function efficacy, 586 (7 studies)**	**RR = 1.41** (1.15 to 1.71)	**96 per 285 (33.7%)**	**145 per 301 48.2%)** (38.8% to 56.4%)	**15.9% higher** (5.1% to 22.7% higher)	⊕⊕⊕⊖ **moderate due to risk of bias[1]**	Probably to improve NYHA cardiac function efficacy.

**Outcomes,** no of participants (studies)	**Relative effect** (95% CI)	**Anticipated absolute effect (95% CI)** **Control group** (CT / CT + placebo)	**Test group** (Qili Qiangxin Capsule + CT)	**Difference**	**Quality of the evidence (GRADE)**	**What happens**
**6-minute walk test (6-MWT), 232 (3 studies)**	—	—	—	**67.4 meters more** (2.17 to 132.63 more)	⊕⊖⊖⊖ **very low due to risk of bias[1], imprecision[2] and inconsistency[3]**	Uncertain to increase 6-minute walk test distance.

**Outcomes,** no of participants (studies)	**Relative effect** (95% CI)	**Anticipated absolute effect (95% CI)** **Control group** (CT / CT + placebo)	**Test group** (Qili Qiangxin Capsule + CT)	**Difference**	**Quality of the evidence (GRADE)**	**What happens**
**Brain natriuretic peptide (BNP), 414 (5 studies)**	—	—	—	**93.46 pg/mL less** (64.93 to 121.98 less)	⊕⊕⊖⊖ **low due to risk of bias[1] and**	May decrease the level of BNP.

					**inconsistency[4]**	

**Outcomes,** no of participants (studies)	**Relative effect** (95% CI)	**Anticipated absolute effect (95% CI)**			**Quality of the evidence (GRADE)**	**What happens**
		**Control group** (CT / CT + placebo)	**Test group** (Qili Qiangxin Capsule + CT)	**Difference**		
**N-terminal pro-brain natriuretic peptide (NT-proBNP), 222 (3 studies)**	—	—	—	**76.62 pg/mL less** (34.92 to 118.32 less)	⊕⊕⊖⊖ **low due to risk of bias[1] and imprecision[2]**	May decrease the level of NT-proBNP.

**Outcomes,** no of participants (studies)	**Relative effect** (95% CI)	**Anticipated absolute effect (95% CI)**			**Quality of the evidence (GRADE)**	**What happens**
		**Control group** (CT / CT + placebo)	**Test group** (Qili Qiangxin Capsule + CT)	**Difference**		
**the ratio of peak mitral valve blood flow velocity in early left ventricular diastole to peak mitral valve blood flow velocity in atrial systole (E/A), 406 (5 studies)**	—	—	—	**0.14 higher** (0.07 to 0.21 higher)	⊕⊕⊖⊖ **low due to risk of bias[1] and inconsistency[5]**	May increase E/A.

Abbreviations: CT, conventional treatment; CI, confidence interval; RR, risk ratio; GRADE, Grading of Recommendations Assessment, Development and Evaluation.

***** The basis for the **risk in the control group** (e.g., the median control group risk across studies) is provided in footnotes. The **risk in the intervention group** (and its 95% confidence interval) is based on the assumed risk in the comparison group and the **relative effect** of the intervention (and its 95% CI).

[1] High risk of bias due to unmentioned allocation concealment and insufficient blind method implementation.

[2] Imprecision due to few events.

[3] Serious unexplained inconsistency (large heterogeneity I^2 = 95%, P-value (P < 0.00001), point estimates, and confidence intervals vary considerably).

[4] Serious unexplained inconsistency (large heterogeneity I^2 = 51%, *P*-value (*P* = 0.09), point estimates, and confidence intervals vary considerably).

[5] Serious unexplained inconsistency (large heterogeneity I^2 = 78%, *P*-value (*P* = 0.001), point estimates, and confidence intervals vary considerably).

[27] Efficacy and safety of Qili Qiangxin Capsules in the treatment of diastolic heart failure: a meta-analysis (Author: Y. Feng, Y.S. Jiang, Q.W. He, and Y.G. Wang)

Patient or population: diastolic heart failure (DHF)
Settings: inpatients and outpatient
Intervention: Qili Qiangxin Capsule + CT
Comparison: CT

**Outcomes,** no of participants (studies)	**Relative effect** (95% CI)	**Anticipated absolute effect (95% CI)** **Control group** (CT)	**Test group** (Qili Qiangxin Capsule + CT)	**Difference**	**Quality of the evidence (GRADE)**	**What happens**
**Clinical comprehensive efficacy, 1220 (14 studies)**	**RR = 1.29** (1.21 to1.36)	**420 per 602 (69.8%)**	**556 per 618 (90.0%)** (84.5% to 94.9%)	**20.2% higher** (14.7% to 25.1% higher)	⊕⊕⊖⊖ **low due to risk of bias[1] and publication bias[2]**	May improve the clinical comprehensive efficacy.
**Outcomes,** no of participants (studies)	**Relative effect** (95% CI)	**Anticipated absolute effect (95% CI)** **Control group** (CT)	**Test group** (Qili Qiangxin Capsule + CT)	**Difference**	**Quality of the evidence (GRADE)**	**What happens**
**TCM symptom efficacy, 442 (6 studies)**	**RR = 1.24** (1.14 to1.35)	**163 per 221 (73.8%)**	**202 per 221 (91.4%)** (84.1% to 99.6%)	**17.6% higher** (10.3% to 25.8% higher)	⊕⊕⊕⊖ **moderate due to risk of bias[1]**	Probably to improve the TCM symptom efficacy.
**Outcomes,** no of participants (studies)	**Relative effect** (95% CI)	**Anticipated absolute effect (95% CI)** **Control group** (CT)	**Test group** (Qili Qiangxin Capsule + CT)	**Difference**	**Quality of the evidence (GRADE)**	**What happens**
**terminal pro-brain natriuretic peptide (NT-proBNP) [subgroup analysis according to course of treatment]**						
**NT-proBNP** *Course of treatment = 1 month*, 889 (11 studies)	—	—	—	**247.18 pg/mL less** (336.12 to 158.25 less)	⊕⊖⊖⊖ **low due to risk of bias[1], publication bias[2] and**	Uncertain to decrease the level of NT-proBNP when the caourse of treatment is 1

					**inconsistency[3]**	month.
**NT-proBNP** *Course of treatment = 6 months*, 179 (2 studies)	—	—	—	**384.00 pg/mL less** (416.97 to 351.03 less)	⊕⊕⊖⊖ **low due to risk of bias[1] and imprecision[4]**	May decrease the level of NT-proBNP when the caourse of treatment is 6 months.
**Outcomes,** no of participants (studies)	**Relative effect** (95% CI)	**Anticipated absolute effect (95% CI)** **Control group** (CT / CT + placebo)	**Test group** (Qili Qiangxin Capsule + CT)	**Difference**	**Quality of the evidence (GRADE)**	**What happens**
**the ratio of peak mitral valve blood flow velocity in early left ventricular diastole to peak mitral valve blood flow velocity in atrial systole (E/A), 614 (8 studies)**	—	—	—	**0.16 higher** (0.10 to 0.23 higher)	⊕⊕⊖⊖ **low due to risk of bias[1] and inconsistency[5]**	May increase the E/A.
**Outcomes,** no of participants (studies)	**Relative effect** (95% CI)	**Anticipated absolute effect (95% CI)** **Control group** (CT / CT + placebo)	**Test group** (Qili Qiangxin Capsule + CT)	**Difference**	**Quality of the evidence (GRADE)**	**What happens**
**the ratio of peak mitral valve blood flow velocity in early diastole to peak mitral valve annulus velocity in early diastole (E/E'), 508 (7 studies)**	—	—	—	**1.97 lower** (2.65 to 1.30 lower)	⊕⊕⊕⊖ **moderate due to risk of bias[1]**	Probably to decrease the E/E'.
**Outcomes,** no of participants (studies)	**Relative effect** (95% CI)	**Anticipated absolute effect (95% CI)** **Control group** (CT)	**Test group** (Qili Qiangxin Capsule + CT)	**Difference**	**Quality of the evidence (GRADE)**	**What happens**
**Adverse events, 503 (6**	**RD = -0.02**	**2.33%**	**0.39%**	**1.94% lower**	⊕⊖⊖⊖	It is uncertain that there is little

**studies)**	(-0.05 to 0.01)	(3 cases of hypokalemia and 3 cases of hypotension)	(1 cases of hypokalemia)		**very low due to risk of bias[1] and very serious imprecision[4, 6]**	or no difference in adverse events.

Abbreviations: CT, conventional treatment; CI, confidence interval; RR, risk ratio; RD: risk difference; GRADE, Grading of Recommendations Assessment, Development and Evaluation; TCM, traditional Chinese medicine.

***** The basis for the **risk in the control group** (e.g., the median control group risk across studies) is provided in footnotes. The **risk in the intervention group** (and its 95% confidence interval) is based on the assumed risk in the comparison group and the **relative effect** of the intervention (and its 95% CI).

[1] High risk of bias due to unmentioned allocation concealment and insufficient blind method implementation.

[2] The funnel chart is asymmetric, suggesting publication bias.

[3] Serious unexplained inconsistency (large heterogeneity I^2 = 87.5%, *P*-value (P = 0.005), point estimates, and confidence intervals vary considerably).

[4] Imprecision due to few events.

[5] Serious unexplained inconsistency (large heterogeneity I^2 = 77%, *P*-value ($P < 0.0001$), point estimates, and confidence intervals vary considerably).

[6] Imprecision due to the confidence interval crossing the invalid line zero.

[28] Comparative analysis of clinical effects according to syndrome differentiation of Qili Qiangxin Capsules on ischemic heart failure: a meta-analysis (Author: Y.L. Sun, X.F. Ruan, Y.P. Li, and X.L. Wang)

Patient or population: heart failure caused by ischemic cardiomyopathy (HF-ICM)

Settings: inpatients and outpatient

Intervention: Qili Qiangxin Capsule + CT

Comparison: CT / CT + placebo

**Outcomes,** no of participants (studies)	**Relative effect** (95% CI)	**Anticipated absolute effect (95% CI)** **Control group** (CT / CT + placebo)	**Test group** (Qili Qiangxin Capsule + CT)	**Difference**	**Quality of the evidence (GRADE)**	**What happens**
**Clinical comprehensive efficacy, 1611 (19 studies)**	**RR = 1.21** (1.16 to1.27)	**608 per 800 (76.0%)**	**748 per 811 (92.2%)** (88.2% to 96.5%)	**16.2% higher** (12.2% to 20.5% higher)	⊕⊕⊕⊖ **moderate due to risk of bias[1]**	Probably to improve clinical comprehensive efficacy.

**Outcomes,** no of participants (studies)	**Relative effect** (95% CI)	**Anticipated absolute effect (95% CI)** **Control group** (CT / CT + placebo)	**Test group** (Qili Qiangxin Capsule + CT)	**Difference**	**Quality of the evidence (GRADE)**	**What happens**
**6-minute walk test (6-MWT), 776 (8 studies)**	—	—	—	**33.20 meters more** (24.70 to 41.70 more)	⊕⊕⊖⊖ **low due to risk of bias[1] and inconsistency[2]**	May increase 6-minute walk test distance.

**Outcomes,** no of participants (studies)	**Relative effect** (95% CI)	**Anticipated absolute effect (95% CI)** **Control group** (CT)	**Test group** (Qili Qiangxin Capsule + CT)	**Difference**	**Quality of the evidence (GRADE)**	**What happens**
**Left ventricular ejection fraction (LVEF), 1563 (16 studies)**	—	—	—	**7.28% more** (5.18% to 9.38% more)	⊕⊕⊖⊖ **low due to risk of bias[1] and inconsistency[3]**	May increase LVEF.

**Outcomes,** no of participants (studies)	**Relative effect** (95% CI)	**Anticipated absolute effect (95% CI)** **Control group** (CT / CT + placebo)	**Test group** (Qili Qiangxin Capsule + CT)	**Difference**	**Quality of the evidence (GRADE)**	**What happens**
**Left ventricular end-diastolic diameter (LVEDD), 947 (10 studies)**	—	—	—	**4.61 mm less** (3.84 to 5.38 less)	⊕⊕⊕⊖ **moderate due to risk of bias[1]**	Probably to decrease LVEDD.
**Outcomes,** no of participants (studies)	**Relative effect** (95% CI)	**Anticipated absolute effect (95% CI)** **Control group** (CT / CT + placebo)	**Test group** (Qili Qiangxin Capsule + CT)	**Difference**	**Quality of the evidence (GRADE)**	**What happens**
**Left ventricular end-diastolic volume (LVEDV), 387 (4 studies)**	—	—	—	**34.43 mL less** (30.05 to 38.81 less)	⊕⊕⊖⊖ **low due to risk of bias[1] and imprecision[4]**	May decrease LVEDV.
**Outcomes,** no of participants (studies)	**Relative effect** (95% CI)	**Anticipated absolute effect (95% CI)** **Control group** (CT / CT + placebo)	**Test group** (Qili Qiangxin Capsule + CT)	**Difference**	**Quality of the evidence (GRADE)**	**What happens**
**Left ventricular end-systolic volume (LVEDV), 387 (4 studies)**	—	—	—	**9.06 mL less** (6.05 to 13.16 less)	⊕⊕⊖⊖ **low due to risk of bias[1] and imprecision[4]**	May decrease LVESV.

Abbreviations: CT, conventional treatment; CI, confidence interval; RR, risk ratio; GRADE, Grading of Recommendations Assessment, Development and Evaluation.

* The basis for the **risk in the control group** (e.g., the median cImprecisionontrol group risk across studies) is provided in footnotes. The **risk in the intervention group** (and its 95% confidence interval) is based on the assumed risk in the comparison group and the **relative effect** of the intervention (and its 95% CI).

[1] High risk of bias due to unmentioned allocation concealment and insufficient blind method implementation.

[2] Serious unexplained inconsistency (large heterogeneity I^2 = 51%, P-value (P = 0.05), point estimates, and confidence intervals vary considerably).

[3] Serious unexplained inconsistency (large heterogeneity I^2 = 92%, *P*-value (*P* < 0.00001), point estimates, and confidence intervals vary considerably).

[4] Imprecision due to few events.

[29] Clinical effects of a standardized Chinese herbal remedy, Qili Qiangxin, as an adjuvant treatment in heart failure: systematic review and meta-analysis (Author: J. Sun, K. Zhang, W.J. Xiong et al.)

Patient or population: heart failure
Settings: inpatients and outpatient
Intervention: Qili Qiangxin Capsule + CT
Comparison: CT / CT + placebo

**Outcomes,** no of participants (studies)	**Relative effect** (95% CI)	**Anticipated absolute effect* (95% CI)** **Control group** (CT)	**Test group** (Qili Qiangxin Capsule + CT)	**Difference**	**Quality of the evidence (GRADE)**	**What happens**
**Mortality, 539 (6 studies)**	**RR = 0.53** (0.27 to 1.07)	**3.8%**	**7.2%**	**3.4% lower**	⊕⊕⊖⊖ **low due to risk of bias[1] and imprecision[2]**	There may be little or no difference in mortality.

**Outcomes,** no of participants (studies)	**Relative effect** (95% CI)	**Anticipated absolute effect* (95% CI)** **Control group** (CT)	**Test group** (Qili Qiangxin Capsule + CT)	**Difference**	**Quality of the evidence (GRADE)**	**What happens**
**Hospitalization rate, 669 (9 studies)**	**RR = 0.49** (0.38 to 0.64)	**16.7%**	**34.2%**	**17.5% lower**	⊕⊕⊕⊖ **moderate due to risk of bias[1]**	Probably to decrease hospitalization rate.

**Outcomes,** no of participants (studies)	**Relative effect** (95% CI)	**Anticipated absolute effect* (95% CI)** **Control group** (CT)	**Test group** (Qili Qiangxin Capsule + CT)	**Difference**	**Quality of the evidence (GRADE)**	**What happens**

**New York Heart Association (NYHA) cardiac function efficacy, 4603 (54 studies)**	**RR = 1.38** (0.29 to 1.48)	**33.6%**	**46.4%**	**12.8% higher**	⊕⊕⊖⊖ **low due to risk of bias[1] and imprecision[3]**	There may be little or no difference in the NYHA cardiac function efficacy.
**Outcomes,** no of participants (studies)	**Relative effect** (95% CI)	**Anticipated absolute effect (95% CI)**			**Quality of the evidence (GRADE)**	**What happens**
		**Control group** (CT / CT + placebo)	**Test group** (Qili Qiangxin Capsule + CT[QQC])	**Difference**		
**6-minute walk test (6-MWT) [subgroup analysis according to interventions]**						
**6-MWT** *QQC+CT vs CT*, - (42 studies)	—	—	—	**47.21 meters more** (44.53 to 49.90 more)	⊕⊕⊖⊖ **due to risk of bias[1] and inconsistency[4]**	May increase 6-minute walk test distance.
**6-MWT** *QQC+CT vs CT+placebo*, 681 (3 studies)	—	—	—	**49.55 meters more** (38.79 to 60.32 more)	⊕⊕⊕⊖ **moderate due to risk of bias[1]**	Probably to increas 6-minute walk test distance.
**Outcomes,** no of participants (studies)	**Relative effect** (95% CI)	**Anticipated absolute effect (95% CI)**			**Quality of the evidence (GRADE)**	**What happens**
		**Control group** (CT)	**Test group** (Qili Qiangxin Capsule + CT)	**Difference**		
**N-terminal pro-brain natriuretic peptide (NT-proBNP), - (24 studies)**	—	—	—	**214.43 pg/mL less** (269.42 to 159.45 less)	⊕⊕⊖⊖ **low due to risk of bias[1] and inconsistency[4]**	May decrease the level of NT-proBNP.
**Outcomes,** no of participants (studies)	**Relative effect** (95% CI)	**Anticipated absolute effect (95% CI)**			**Quality of the evidence (GRADE)**	**What happens**
		**Control group** (CT / CT + placebo)	**Test group** (Qili Qiangxin Capsule + CT)	**Difference**		

**Left ventricular ejection fraction (LVEF), - (84 studies)**	—	—	—	**5.87% more** (5.28% to 6.47% more)	⊕⊕⊖⊖ **low due to risk of bias[1] and inconsistency[5]**	May increase the LVEF.
**Outcomes,** no of participants (studies)	**Relative effect** (95% CI)	**Anticipated absolute effect (95% CI)**			**Quality of the evidence (GRADE)**	**What happens**
		**Control group** (CT)	**Test group** (Qili Qiangxin Capsule + CT)	**Difference**		
**Adverse events, 4846 (56 studies)**	**RR = 0.56** (0.40 to 0.78)	**3.5%**	**2.0%**	**1.5% lower**	⊕⊕⊕⊖ **moderate due to risk of bias[1]**	It is probably that Qili Qiangxin combined with conventional treatment can significantly reduce risk of adverse events.

Abbreviations: CT, conventional Treatment; CI, confidence interval; RR, risk ratio; GRADE, Grading of Recommendations Assessment, Development and Evaluation.

* The basis for the **risk in the control group** (e.g., the median control group risk across studies) is provided in footnotes. The **risk in the intervention group** (and its 95% confidence interval) is based on the assumed risk in the comparison group and the **relative effect** of the intervention (and its 95% CI).

[1] High risk of bias due to unmentioned allocation concealment and insufficient blind method implementation.

[2] Imprecision due to few events.

[3] Most of the trials have wide range of 95% CI for effect estimate.

[4] Serious unexplained inconsistency (large heterogeneity I^2 = 96%, point estimates, and confidence intervals vary considerably).

[5] Serious unexplained inconsistency (large heterogeneity I^2 = 91%, point estimates, and confidence intervals vary considerably).

[30] Routine western medicine treatment plus Qishen Yiqi Dripping Pills for treating patients with chronic heart failure: a systematic review of randomized control trials (Author: S.H. Wang, J.Y. Mao, Y.Z. Hou, J.Y. Wang, X.L. Wang, and Z.J. Li)

Patient or population: chronic heart failure
Settings: inpatients and outpatient
Intervention: Qishen Yiqi Dripping Pill (0.5g, tid) + CT
Comparison: CT

**Outcomes,** no of participants (studies)	**Relative effect** (95% CI)	**Anticipated absolute effect* (95% CI)**			**Quality of the evidence (GRADE)**	**What happens**
		**Control group** (CT)	**Test group** (Qishen Yiqi Dripping Pill + CT)	**Difference**		
**Mortality (follow-up: 1 year), 83 (1 study)**	—	**13 per 37 (35.14%)**	**7 per 46 (15.22%)**	**19.92% lower**	⊕⊕⊖⊖ **low due to risk of bias[1] and imprecision[2]**	May decrease mortality when the follow-up period is 1 year.

**Outcomes,** no of participants (studies)	**Relative effect** (95% CI)	**Anticipated absolute effect* (95% CI)**			**Quality of the evidence (GRADE)**	**What happens**
		**Control group** (CT)	**Test group** (Qishen Yiqi Dripping Pill + CT)	**Difference**		
**Hospitalization rate, 248 (2 studies)**	**RR = 0.52** (0.33 to 0.81)	**37 per 113 (32.7%)**	**23 per 135 (17.0%)** (10.8% to 26.5%)	**15.7% lower** (6.2% to 21.9% lower)	⊕⊕⊕⊖ **moderate due to risk of bias[1]**	Probably to decrease the hospitalization rate.

**Outcomes,** no of participants (studies)	**Relative effect** (95% CI)	**Anticipated absolute effect (95% CI)**			**Quality of the evidence (GRADE)**	**What happens**
		**Control group** (CT)	**Test group** (Qili Qiangxin Capsule + CT)	**Difference**		

**Clinical comprehensive efficacy, 887(7 studies)**	**RR = 1.18** (1.12 to1.25)	**352 per 444 (79.3%)**	**415 per 443 (93.7%)** (88.8% to 99.1%)	**14.4% higher** (9.5% to 19.8% higher)	⊕⊕⊕⊝ **moderate due to risk of bias[1]**	Probably to improve the clinical comprehensive efficacy.
**Outcomes,** no of participants (studies)	**Relative effect** (95% CI)	**Anticipated absolute effect* (95% CI)**			**Quality of the evidence (GRADE)**	**What happens**
		**Control group** (CT)	**Test group** (Qishen Yiqi Dripping Pill + CT)	**Difference**		
**New York Heart Association (NYHA) cardiac function efficacy, 628 (7 studies)**	**RR = 1.18** (1.10 to 1.27)	**235 per 303 (77.6%)**	**298 per 325 (91.7%)** (85.4% to 98.6%)	**14.1% higher** (7.8% to 21.0% higher)	⊕⊕⊕⊝ **moderate due to risk of bias[1]**	Probably to improve the NYHA cardiac function efficacy.
**Outcomes,** no of participants (studies)	**Relative effect** (95% CI)	**Anticipated absolute effect (95% CI)**			**Quality of the evidence (GRADE)**	**What happens**
		**Control group** (CT)	**Test group** (Qili Qiangxin Capsule + CT)	**Difference**		
**6-minute walk test (6-MWT), 882 (7 studies)**	—	—	—	**94.39 meters more** (71.89 to 116.89 more)	⊕⊕⊝⊝ **low due to risk of bias[1] and inconsistency[3]**	May increase the 6-minute walk test distance.
**Outcomes,** no of participants (studies)	**Relative effect** (95% CI)	**Anticipated absolute effect (95% CI)**			**Quality of the evidence (GRADE)**	**What happens**
		**Control group** (CT)	**Test group** (Qishen Yiqi Dripping Pill + CT)	**Difference**		
**Brain natriuretic peptide (BNP), 478 (5 studies)**	—	—	—	**194.85 pg/mL less** (442.61 less to 52.91 more)	⊕⊝⊝⊝ **low due to risk of bias[1], inconsistency[4] and imprecision[5]**	It is uncertain that there is little or no difference in the level of BNP.
**Outcomes,** no of participants (studies)	**Relative effect** (95% CI)	**Anticipated absolute effect (95% CI)**			**Quality of the evidence (GRADE)**	**What happens**
		**Control group**	**Test group**	**Difference**		

		(CT)	(Qishen Yiqi Dripping Pill + CT)			
**Left ventricular ejection fraction (LVEF) [subgroup analysis according to patient]**						
**LVEF**<br>*chronic heart failure caused by coronary heart disease*, 403 (3 studies)	—	—	—	**8.34% more**<br>(6.23% to10.45% more)	⊕⊕⊕⊖<br>**low due to risk of bias[1]**	Probably to increase the LVEF.
**LVEF**<br>*chronic heart failure caused by other diseases*, 1272 (12 studies)	—	—	—	**5.57% more**<br>(4.16% to 6.97% more)	⊕⊖⊖⊖<br>**very low due to risk of bias[1] , inconsistency[6] and publication bias[7]**	Uncertain to increase the LVEF.

Abbreviations: CT, conventional Treatment; CI, confidence interval; RR, risk ratio; GRADE, Grading of Recommendations Assessment, Development and Evaluation.

* The basis for the **risk in the control group** (e.g., the median control group risk across studies) is provided in footnotes. The **risk in the intervention group** (and its 95% confidence interval) is based on the assumed risk in the comparison group and the **relative effect** of the intervention (and its 95% CI).

[1] High risk of bias due to unmentioned allocation concealment and insufficient blind method implementation.

[2] Imprecision due to few events.

[3] Serious unexplained inconsistency (large heterogeneity I^2 = 95%, p < 0.00001, point estimates, and confidence intervals vary considerably).

[4] Serious unexplained inconsistency (large heterogeneity I^2 = 99%, p < 0.00001, point estimates, and confidence intervals vary considerably).

[5] Imprecision due to the confidence interval crossing the invalid line zero.

[6] Serious unexplained inconsistency (large heterogeneity I^2 = 86%, p < 0.00001, point estimates, and confidence intervals vary considerably).

[7] The funnel chart is asymmetric, suggesting publication bias.

[31] Meta-analysis of Qishenyiqi Dripping Pills for chronic heart failure (Author: J.G. Liu, W.H. Gu, X.Q. Liu, X.S. Li, and Q.J. Huang)

Patient or population: chronic heart failure (CHF)
Settings: inpatients and outpatient
Intervention: Qishen Yiqi Dripping Pill (0.5g, tid) + CT
Comparison: CT

**Outcomes**, no of participants (studies)	**Relative effect** (95% CI)	**Anticipated absolute effect* (95% CI)** Control group (CT)	Test group (Qishen Yiqi Dripping Pill + CT)	Difference	**Quality of the evidence (GRADE)**	**What happens**
**Hospitalization rate, 365 (3 studies)**	**OR = 0.41** (0.23 to 0.72)	**45 per 176 (25.6%)**	**24 per 189 (12.7%)**	**12.9% lower**	⊕⊕⊕⊝ **moderate due to risk of bias[1]**	Probably to decrease hospitalization rate.

**Outcomes**, no of participants (studies)	**Relative effect** (95% CI)	**Anticipated absolute effect* (95% CI)** Control group (CT)	Test group (Qishen Yiqi Dripping Pill + CT)	Difference	**Quality of the evidence (GRADE)**	**What happens**
**New York Heart Association (NYHA) cardiac function efficacy, 2007 (16 studies)**	**RD = 0.14** (0.11 to 0.17)	**758 per 979 (77.4%)**	**945 per 1028 (91.9%)**	**14.5% higher**	⊕⊕⊝⊝ **low due to risk of bias[1] and publication bias[2]**	May improve NYHA cardiac function efficacy.

**Outcomes**, no of participants (studies)	**Relative effect** (95% CI)	**Anticipated absolute effect (95% CI)** Control group (CT)	Test group (Qishen Yiqi Dripping Pill + CT)	Difference	**Quality of the evidence (GRADE)**	**What happens**

Outcomes, no of participants (studies)	Relative effect (95% CI)	Anticipated absolute effect (95% CI)			Quality of the evidence (GRADE)	What happens
**Left ventricular end-diastolic diameter (LVEDD), 510 (5 studies)**	**SMD = -0.54** (-0.76 to -0.31)	—	—	—	⊕⊕⊖⊖ **low due to risk of bias[1] and inconsistency[3]**	May decrease LVEDD.
**Outcomes,** no of participants (studies)	**Relative effect** (95% CI)	**Anticipated absolute effect (95% CI)**			**Quality of the evidence (GRADE)**	**What happens**
		**Control group** (CT)	**Test group** (Qishen Yiqi Dripping Pill + CT)	**Difference**		
**Left ventricular end-systolic diameter (LVESD), 450 (5 studies)**	**SMD = -0.53** (-0.72 to -0.34)	—	—	—	⊕⊕⊖⊖ **moderate due to risk of bias[1] and inconsistency[3]**	May decrease LVESD.
**Outcomes,** no of participants (studies)	**Relative effect** (95% CI)	**Anticipated absolute effect (95% CI)**			**Quality of the evidence (GRADE)**	**What happens**
		**Control group** (CT)	**Test group** (Qishen Yiqi Dripping Pill + CT)	**Difference**		
**Cardiac output (CO), 430 (3 studies)**	**SMD = 1.43** (1.22 to 1.64)	—	—	—	⊕⊕⊖⊖ **moderate due to risk of bias[1] and inconsistency[3]**	May increase CO.

Abbreviations: CT, conventional Treatment; CI, confidence interval; OR: odds ratio; RD: risk difference; SMD: standardized mean difference; GRADE, Grading of Recommendations Assessment, Development and Evaluation.

***** The basis for the **risk in the control group** (e.g., the median control group risk across studies) is provided in footnotes. The **risk in the intervention group** (and its 95% confidence interval) is based on the assumed risk in the comparison group and the **relative effect** of the intervention (and its 95% CI).

[1] High risk of bias due to unmentioned allocation concealment and insufficient blind method implementation.

[2] The funnel chart is asymmetric, suggesting publication bias.

[3] Unexplained inconsistency.

[32] Meta-analysis of the efficacy and safety of Qishenyiqi Dripping Pills on chronic congestive heart failure (Author: C.C. Gao, G.L. Xu, and L. Qin)

Patient or population: chronic heart failure (CHF)

Settings: inpatients and outpatient

Intervention: Qishen Yiqi Dripping Pill (0.5g, tid) + CT

Comparison: CT

**Outcomes,** no of participants (studies)	**Relative effect** (95% CI)	**Anticipated absolute effect* (95% CI)**			**Quality of the evidence (GRADE)**	**What happens**
		**Control group** (CT)	**Test group** (Qishen Yiqi Dripping Pill + CT)	**Difference**		
**New York Heart Association (NYHA) cardiac function efficacy, 1590 (13 studies)**	**OR = 2.75** (2.07 to 3.66)	**585 per 766 (76.4%)**	**740 per 824 (89.8%)**	**13.4% higher**	⊕⊕⊖⊖ **low due to risk of bias[1] and publication bias[2]**	May improve NYHA cardiac function efficacy.

Abbreviations: CT, conventional Treatment; CI, confidence interval; OR: odds ratio; GRADE, Grading of Recommendations Assessment, Development and Evaluation.

* The basis for the **risk in the control group** (e.g., the median control group risk across studies) is provided in footnotes. The **risk in the intervention group** (and its 95% confidence interval) is based on the assumed risk in the comparison group and the **relative effect** of the intervention (and its 95% CI).

[1] High risk of bias due to unmentioned allocation concealment and insufficient blind method implementation.

[2] The funnel chart is asymmetric, suggesting publication bias.

[33] Meta-analysis of Qishen Yiqi Dripping Pills in the treatment of chronic heart failure (Y. Wang and X.H. Dai)

Patient or population: chronic heart failure (CHF)

Settings: inpatients and outpatient

Intervention: Qishen Yiqi Dripping Pill (0.5g, tid) + CT

Comparison: CT

**Outcomes,** no of participants (studies)	**Relative effect** (95% CI)	**Anticipated absolute effect* (95% CI)** Control group (CT)	Test group (Qishen Yiqi Dripping Pill + CT)	Difference	**Quality of the evidence (GRADE)**	**What happens**
**Clinical comprehensive efficacy, 1791 (16 studies)**	**OR = 3.82** (2.83 to 5.16)	**690 per 888 (77.7%)**	**839 per 903 (92.9%)**	**15.2% lower**	⊕⊕⊖⊖ **low due to risk of bias[1] and publication bias[2]**	May improve clinical comprehensive efficacy.
**Outcomes,** no of participants (studies)	**Relative effect** (95% CI)	**Anticipated absolute effect* (95% CI)** Control group (CT)	Test group (Qishen Yiqi Dripping Pill + CT)	Difference	**Quality of the evidence (GRADE)**	**What happens**
**New York Heart Association (NYHA) cardiac function efficacy, 576 (6 studies)**	**OR = 4.68** (2.65 to 8.26)	**211 per 268 (78.7%)**	**289 per 308 (93.8%)**	**15.1% higher**	⊕⊕⊕⊖ **moderate due to risk of bias[1]**	Probably to improve NYHA cardiac function efficacy.
**Outcomes,** no of participants (studies)	**Relative effect** (95% CI)	**Anticipated absolute effect (95% CI)** Control group (CT)	Test group (Qishen Yiqi Dripping Pill + CT)	Difference	**Quality of the evidence (GRADE)**	**What happens**

**6-minute walk test (6-MWT), 584 (6 studies)**	—	—	—	**73.59 meters more** (67.09 to 80.09 more)	⊕⊕⊕⊝ **moderate due to risk of bias**[1]	Probably to increase 6-minute walk test distance.
**Outcomes,** no of participants (studies)	**Relative effect** (95% CI)	**Anticipated absolute effect (95% CI)** **Control group** (CT)	**Test group** (Qishen Yiqi Dripping Pill + CT)	**Difference**	**Quality of the evidence (GRADE)**	**What happens**
**Brain natriuretic peptide (BNP), 338 (4 studies)**	—	—	—	**48.92 pg/mL less** (40.35 to 57.49 less)	⊕⊕⊝⊝ **low due to risk of bias**[1] **and imprecision**[3]	May decrease the level of BNP.
**Outcomes,** no of participants (studies)	**Relative effect** (95% CI)	**Anticipated absolute effect (95% CI)** **Control group** (CT)	**Test group** (Qishen Yiqi Dripping Pill + CT)	**Difference**	**Quality of the evidence (GRADE)**	**What happens**
**Left ventricular ejection fraction (LVEF), 2038 (18 studies)**	—	—	—	**7.61% more** (6.24% to 8.98% more)	⊕⊕⊝⊝ **moderate due to risk of bias**[1] **and publication bias**[2]	May increase LVEF.
**Outcomes,** no of participants (studies)	**Relative effect** (95% CI)	**Anticipated absolute effect (95% CI)** **Control group** (CT)	**Test group** (Qishen Yiqi Dripping Pill + CT)	**Difference**	**Quality of the evidence (GRADE)**	**What happens**
**Left ventricular end-diastolic diameter (LVEDD), 411 (4 studies)**	—	—	—	**1.19 mm less** (0.37 to 2.01 less)	⊕⊕⊕⊝ **moderate due to risk of bias**[1]	Probably to decrease LVEDD.
**Outcomes,** no of participants (studies)	**Relative effect** (95% CI)	**Anticipated absolute effect (95% CI)** **Control group** (CT)	**Test group** (Qishen Yiqi Dripping Pill + CT)	**Difference**	**Quality of the evidence (GRADE)**	**What happens**

**Left ventricular end-systolic diameter (LVESD), 551 (5 studies)**	—	—	—	**1.48 mm less** (0.83 to 2.12 less)	⊕⊕⊕⊖ **moderate due to risk of bias**[1]	Probably to decrease LVESD.

Abbreviations: CT, conventional Treatment; CI, confidence interval; OR: odds ratio; GRADE, Grading of Recommendations Assessment, Development and Evaluation.

***** The basis for the **risk in the control group** (e.g., the median control group risk across studies) is provided in footnotes. The **risk in the intervention group** (and its 95% confidence interval) is based on the assumed risk in the comparison group and the **relative effect** of the intervention (and its 95% CI).

[1] High risk of bias due to unmentioned allocation concealment and insufficient blind method implementation.

[2] The funnel chart is asymmetric, suggesting publication bias.

[3] Imprecision due to few events.

[34] Meta-analysis of Qishen Yiqi Dripping Pills in preventing ventricular remodeling in patients with chronic heart failure

(MQ.Y. Shan, W. Zhang, L. Lv, L.Y. Li, H. Sun,and Z.X. Guo)

Patient or population: chronic heart failure (CHF)

Settings: inpatients and outpatient

Intervention: Qishen Yiqi Dripping Pill + CT

Comparison: CT

**Outcomes,** no of participants (studies)	**Relative effect** (95% CI)	**Anticipated absolute effect (95% CI)**			**Quality of the evidence (GRADE)**	**What happens**
		**Control group** (CT)	**Test group** (Qishen Yiqi Dripping Pill + CT)	**Difference**		
**Left ventricular end-diastolic diameter (LVEDD), 786 (8 studies)**	—	—	—	**2.55 mm less** (1.47 to 3.63 less)	⊕⊕⊖⊖ **low due to risk of bias[1] and inconsistency[2]**	May decrease LVEDD.
**Outcomes,** no of participants (studies)	**Relative effect** (95% CI)	**Anticipated absolute effect (95% CI)**			**Quality of the evidence (GRADE)**	**What happens**
		**Control group** (CT)	**Test group** (Qishen Yiqi Dripping Pill + CT)	**Difference**		
**Left ventricular end-systolic diameter (LVESD), 786 (8 studies)**	—	—	—	**1.82 mm less** (1.30 to 2.34 less)	⊕⊕⊕⊖ **moderate due to risk of bias[1]**	Probably to decrease LVESD.

Abbreviations: CT, conventional Treatment; CI, confidence interval; OR: odds ratio; RD: risk difference; SMD: standardized mean difference; GRADE, Grading of Recommendations Assessment, Development and Evaluation.

***** The basis for the **risk in the control group** (e.g., the median control group risk across studies) is provided in footnotes. The **risk in the intervention group** (and its 95% confidence interval) is based on the assumed risk in the comparison group and the **relative effect** of the intervention (and its 95% CI).

[1] High risk of bias due to unmentioned allocation concealment and insufficient blind method implementation.

[2] Serious unexplained inconsistency (large heterogeneity I^2 = 71%, P-value (P = 0.001), point estimates, and confidence intervals vary considerably).

[35] Meta-analysis for Qishen Yiqi Dropping Pills in treatment of chronic heart failure with the syndrome of Qi Deficiency and Blood Stasis (Author: Y.L. Zhang, J. Wang, Y. Li, H.H. Zhao, J.J. Liu, and W. Wang)

Patient or population: chronic heart failure (CHF)
Settings: inpatients and outpatient
Intervention: Qishen Yiqi Dripping Pill + CT
Comparison: CT / CT + placebo

**Outcomes**, no of participants (studies)	**Relative effect** (95% CI)	**Anticipated absolute effect* (95% CI)** **Control group** (CT / CT + placebo)	**Test group** (Qishen Yiqi Dripping Pill + CT)	**Difference**	**Quality of the evidence (GRADE)**	**What happens**
**New York Heart Association (NYHA) cardiac function efficacy, 634 (8 studies)**	**RR =1.25** (1.16 to 1.35)	**224 per 316 (70.9%)**	**282 per 318 (88.7%)** (82.2% to 95.7%)	**17.8% higher** (11.3% to 24.8% higher)	⊕⊕⊕⊖ **moderate due to risk of bias[1]**	Probably to improve NYHA cardiac function efficacy.

**Outcomes**, no of participants (studies)	**Relative effect** (95% CI)	**Anticipated absolute effect* (95% CI)** **Control group** (CT / CT + placebo)	**Test group** (Qishen Yiqi Dripping Pill + CT)	**Difference**	**Quality of the evidence (GRADE)**	**What happens**
**TCM symptom efficacy, 338 (5 studies)**	**RR = 1.23** (1.07 to1.43)	**123 per 168 (73.2%)**	**157 per 170 (92.4%)** (78.3% to104.7%)	**19.2% higher** (5.1% to 31.5% higher)	⊕⊕⊖⊖ **low due to risk of bias[1] and inconsistency[2]**	Probably to improve the TCM symptom efficacy.

**Outcomes**, no of participants (studies)	**Relative effect** (95% CI)	**Anticipated absolute effect (95% CI)** **Control group** (CT / CT + placebo)	**Test group** (Qishen Yiqi Dripping Pill + CT)	**Difference**	**Quality of the evidence (GRADE)**	**What happens**
**6-minute walk test (6-MWT), 336 (4 studies)**	—	—	—	**50.13 meters more** (22.32 to 77.93 more)	⊕⊖⊖⊖ **very low due to risk of bias[1], inconsistency[3] and**	Certain to increase the 6-minute walk test distance.

					**imprecision**[4]	

**Outcomes,** no of participants (studies)	**Relative effect** (95% CI)	**Anticipated absolute effect (95% CI)**			**Quality of the evidence (GRADE)**	**What happens**
		**Control group** (CT / CT + placebo)	**Test group** (Qishen Yiqi Dripping Pill + CT)	**Difference**		
**Brain natriuretic peptide (BNP), 370 (4 studies)**	—	—	—	**29.47 pg/mL less** (21.64 to 37.29 less)	⊕⊕⊖⊖ **low due to risk of bias**[1] **and imprecision**[4]	May decrease the level of BNP.

**Outcomes,** no of participants (studies)	**Relative effect** (95% CI)	**Anticipated absolute effect (95% CI)**			**Quality of the evidence (GRADE)**	**What happens**
		**Control group** (CT / CT + placebo)	**Test group** (Qishen Yiqi Dripping Pill + CT)	**Difference**		
**Left ventricular ejection fraction (LVEF), 607 (7 studies)**	—	—	—	**4.64% more** (3,82% to 5.47% more)	⊕⊕⊕⊖ **moderate due to risk of bias**[1]	Probably to increase LVEF.

Abbreviations: CT, conventional Treatment; CI, confidence interval; OR: odds ratio; RD: risk difference; SMD: standardized mean difference; GRADE, Grading of Recommendations Assessment, Development and Evaluation.

***** The basis for the **risk in the control group** (e.g., the median control group risk across studies) is provided in footnotes. The **risk in the intervention group** (and its 95% confidence interval) is based on the assumed risk in the comparison group and the **relative effect** of the intervention (and its 95% CI).

1 High risk of bias due to unmentioned allocation concealment and insufficient blind method implementation.

2 Serious unexplained inconsistency (large heterogeneity I^2 = 56%, *P*-value (*P* = 0.06), point estimates, and confidence intervals vary considerably).

3 Serious unexplained inconsistency (large heterogeneity I^2 = 75%, P-value (P = 0.007), point estimates, and confidence intervals vary considerably).

4 Imprecision due to few events.

[36] Qishenyiqi Dripping Pill improves ventricular remodeling and function in patients with chronic heart failure: a pooled analysis (M. Chang, L. Cheng, Y. Shen, Y. Zhang, Z. Zhang, and P. Hao)

Patient or population: chronic heart failure (CHF)

Settings: inpatients and outpatient

Intervention: Qishen Yiqi Dripping Pill (0.5g, tid) + CT

Comparison: CT

**Outcomes,** no of participants (studies)	**Relative effect** (95% CI)	**Anticipated absolute effect (95% CI)**			**Quality of the evidence (GRADE)**	**What happens**
		**Control group** (CT)	**Test group** (Qishen Yiqi Dripping Pill + CT)	**Difference**		
**LVEF**, 877 (12 studies)	—	—	—	**6.05% more** (0.94% to 11.16% more)	⊕⊕⊝⊝ **low due to risk of bias[1], inconsistency[2] and publication bias[3]**	May increase LVEF.

Abbreviations: CT, conventional Treatment; CI, confidence interval; OR: odds ratio; RD: risk difference; SMD: standardized mean difference; GRADE, Grading of Recommendations Assessment, Development and Evaluation.

* The basis for the **risk in the control group** (e.g., the median control group risk across studies) is provided in footnotes. The **risk in the intervention group** (and its 95% confidence interval) is based on the assumed risk in the comparison group and the **relative effect** of the intervention (and its 95% CI).

[1] High risk of bias due to unmentioned allocation concealment and insufficient blind method implementation.

[2] Serious unexplained inconsistency (large heterogeneity $I^2 = 99\%$, *P*-value ($P < 0.001$), point estimates, and confidence intervals vary considerably).

[3] The funnel chart is asymmetric, suggesting publication bias.

[37] Qishen Yiqi Dropping Pills for ischemic heart failure: a systematic review (F. Qu, D.M. Xing, W.K. Zheng, Y. Tian, Y. Li, and L.Y. Kang)

Patient or population: heart failure caused by ischemic cardiomyopathy (HF-ICM)

Settings: inpatients and outpatient

Intervention: Qishen Yiqi Dripping Pill (0.5g, tid) + CT

Comparison: CT

**Outcomes**, no of participants (studies)	**Relative effect** (95% CI)	**Anticipated absolute effect (95% CI)** Control group (CT / CT + placebo)	Test group (Qishen Yiqi Dripping Pill + CT)	Difference	**Quality of the evidence (GRADE)**	**What happens**
**6-minute walk test (6-MWT), 128 (2 studies)**	—	—	—	**48.31 meters more** (39.51 to 57.10 more)	⊕⊕⊖⊖ **low due to risk of bias[1] and imprecision[2]**	May increase 6-minute walk test distance.
**Outcomes**, no of participants (studies)	**Relative effect** (95% CI)	**Anticipated absolute effect (95% CI)** Control group (CT)	Test group (Qishen Yiqi Dripping Pill + CT)	Difference	**Quality of the evidence (GRADE)**	**What happens**
**Brain natriuretic peptide (BNP), 296 (3 studies)**	—	—	—	**55.76 pg/mL less** (44.99 to 66.53 less)	⊕⊖⊖⊖ **very low due to risk of bias[1], imprecision[2] and inconsistency[3]**	Uncertain to decrease the level of BNP.
**Outcomes**, no of participants (studies)	**Relative effect** (95% CI)	**Anticipated absolute effect (95% CI)** Control group (CT)	Test group (Qishen Yiqi Dripping Pill + CT)	Difference	**Quality of the evidence (GRADE)**	**What happens**
**Left ventricular ejection fraction (LVEF), 673 (6**	—	—	—	**7.00% more** (5.00% to 8.00% more)	⊕⊕⊕⊖ **moderate due to risk of bias[1]**	Probably to increase LVEF.

studies)

Abbreviations: CT, conventional Treatment; CI, confidence interval; GRADE, Grading of Recommendations Assessment, Development and Evaluation.

***** The basis for the **risk in the control group** (e.g., the median control group risk across studies) is provided in footnotes. The **risk in the intervention group** (and its 95% confidence interval) is based on the assumed risk in the comparison group and the **relative effect** of the intervention (and its 95% CI).

[1] High risk of bias due to unmentioned allocation concealment and insufficient blind method implementation.

[2] Imprecision due to few events.

[3] Serious unexplained inconsistency (large heterogeneity $I^2 = 99\%$, *P*-value ($P < 0.00001$), point estimates, and confidence intervals vary considerably).

[38] Meta-analysis of Qishen Yiqi Dripping Pills in the treatment of coronary heart disease and heart failure (Y. Tian and J.X. Gu)

Patient or population: heart failure caused by coronary heart disease (HF-CHD)
Settings: inpatients and outpatient
Intervention: Qishen Yiqi Dripping Pill (0.5g, tid) + CT
Comparison: CT

**Outcomes,** no of participants (studies)	**Relative effect** (95% CI)	**Anticipated absolute effect* (95% CI)**			**Quality of the evidence (GRADE)**	**What happens**
		**Control group** (CT)	**Test group** (Qishen Yiqi Dripping Pill + CT)	**Difference**		
**Clinical comprehensive efficacy, 1298 (12 studies)**	**RR = 1.16** (1.11 to 1.21)	**511 per 644 (79.3%)**	**601 per 654 (92.0%)** (88.0% to 96.0%)	**12.7% higher (8.7% to 16.7% higher)**	⊕⊕⊝⊝ **low due to risk of bias[1] and publication bias[2]**	May improve clinical comprehensive efficacy.

**Outcomes,** no of participants (studies)	**Relative effect** (95% CI)	**Anticipated absolute effect (95% CI)**			**Quality of the evidence (GRADE)**	**What happens**
		**Control group** (CT)	**Test group** (Qishen Yiqi Dripping Pill + CT)	**Difference**		
**6-minute walk test (6-MWT), 1162(11 studies)**	—	—	—	**71.37 meters more** (53.28 to 89.47 more)	⊕⊝⊝⊝ **very low due to risk of bias[1], publication bias[2] and inconsistency[3]**	Uncertain to increase 6-minute walk test distance.

**Outcomes,** no of participants (studies)	**Relative effect** (95% CI)	**Anticipated absolute effect (95% CI)**			**Quality of the evidence (GRADE)**	**What happens**
		**Control group** (CT)	**Test group** (Qishen Yiqi Dripping Pill + CT)	**Difference**		
**Brain natriuretic peptide (BNP), 390 (4**	—	—	—	**63.55 pg/mL less** (41.63 to 85.48 less)	⊕⊝⊝⊝ **very low due to risk of**	Uncertain to decrease the level of BNP.

Outcomes, no of participants (studies)	Relative effect (95% CI)	Anticipated absolute effect (95% CI)			Quality of the evidence (GRADE)	What happens
**studies)**					**bias[1],inconsistency[4] and imprecision[5]**	
**Outcomes,** no of participants (studies)	**Relative effect** (95% CI)	**Control group** (CT)	**Test group** (Qishen Yiqi Dripping Pill + CT)	**Difference**	**Quality of the evidence (GRADE)**	**What happens**
**Left ventricular ejection fraction (LVEF), 1275(12 studies)**	—	—	—	**6.55% more** (5.35% to 7.74% more)	⊕⊝⊝⊝ **very low due to risk of bias[1], publication bias[2] and inconsistency[6]**	Uncertain to increase LVEF.

Abbreviations: CT, conventional Treatment; CI, confidence interval; OR: odds ratio; RD: risk difference; SMD: standardized mean difference; GRADE, Grading of Recommendations Assessment, Development and Evaluation.

***** The basis for the **risk in the control group** (e.g., the median control group risk across studies) is provided in footnotes. The **risk in the intervention group** (and its 95% confidence interval) is based on the assumed risk in the comparison group and the **relative effect** of the intervention (and its 95% CI).

[1] High risk of bias due to unmentioned allocation concealment and insufficient blind method implementation.

[2] The funnel chart is asymmetric, suggesting publication bias.

[3] Serious unexplained inconsistency (large heterogeneity I^2 = 95%, P-value (P < 0.00001), point estimates, and confidence intervals vary considerably).

[4] Serious unexplained inconsistency (large heterogeneity I^2 = 85%, P-value (P = 0.0002), point estimates, and confidence intervals vary considerably).

[5] Imprecision due to few events.

[6] Serious unexplained inconsistency (large heterogeneity I^2 = 62%, P-value (P = 0.002), point estimates, and confidence intervals vary considerably).

[39] The efficacy of Shexiang Baoxin Pills in the adjuvant treatment of chronic heart failure: a meta-analysis (Author: Y.P. An, X. Zou, G.Z. Yao et al.)

Patient or population: chronic heart failure (CHF)
Settings: inpatients and outpatient
Intervention: Shexiang Baoxin Pill (22.5~45mg, tid) + CT
Comparison: CT / CT + placebo

**Outcomes,** no of participants (studies)	**Relative effect** (95% CI)	**Anticipated absolute effect* (95% CI)** **Control group** (CT / CT + placebo)	**Test group** (Shexiang Baoxin Pill + CT)	**Difference**	**Quality of the evidence (GRADE)**	**What happens**
**Clinical comprehensive efficacy, 1327 (15 studies)**	**OR = 3.75** (2.72 to 5.16)	**487 per 662 (73.6%)**	**606 per 665 (91.1%)**	**17.5% higher**	⊕⊕⊖⊖ **moderate due to risk of bias[1] and publication bias[2]**	May improve clinical comprehensive efficacy.
**Outcomes,** no of participants (studies)	**Relative effect** (95% CI)	**Anticipated absolute effect (95% CI)** **Control group** (CT / CT + placebo)	**Test group** (Shexiang Baoxin Pill + CT)	**Difference**	**Quality of the evidence (GRADE)**	**What happens**
**6-minute walk test (6-MWT), 1089(12 studies)**	—	—	—	**38.05 meters more** (26.94 to 49.15 more)	⊕⊕⊖⊖ **low due to risk of bias[1] and inconsistency[3]**	May increase 6-minute walk test distance.
**Outcomes,** no of participants (studies)	**Relative effect** (95% CI)	**Anticipated absolute effect (95% CI)** **Control group** (CT / CT + placebo)	**Test group** (Shexiang Baoxin Pill + CT)	**Difference**	**Quality of the evidence (GRADE)**	**What happens**
**Brain natriuretic peptide (BNP), 437 (5 studies)**	—	—	—	**78.64 pg/mL less** (239.02 less to 81.73 more)	⊕⊖⊖⊖ **very low due to risk of bias[1],inconsistency[4] and**	It is uncertain that there is little or no difference in the level of BNP.

					**imprecision[5]**	

**Outcomes,** no of participants (studies)	**Relative effect** (95% CI)	**Anticipated absolute effect (95% CI)**			**Quality of the evidence (GRADE)**	**What happens**
		**Control group** (CT / CT + placebo)	**Test group** (Shexiang Baoxin Pill + CT)	**Difference**		
**Left ventricular ejection fraction (LVEF), 1320 (15 studies)**	—	—	—	**5.18% more** (3.80% to 6.56% more)	⊕⊕⊝⊝ **low due to risk of bias[1] and inconsistency[6]**	May increase LVEF.

**Outcomes,** no of participants (studies)	**Relative effect** (95% CI)	**Anticipated absolute effect (95% CI)**			**Quality of the evidence (GRADE)**	**What happens**
		**Control group** (CT / CT + placebo)	**Test group** (Shexiang Baoxin Pill + CT)	**Difference**		
**Left ventricular end-diastolic diameter (LVEDD), 680 (8 studies)**	—	—	—	**2.88 mm less** (2.30 to 3.45 less)	⊕⊕⊕⊝ **moderate due to risk of bias[1]**	Probably to decrease LVEDD.

Abbreviations: CT, conventional Treatment; CI, confidence interval; OR: odds ratio; GRADE, Grading of Recommendations Assessment, Development and Evaluation.

***** The basis for the **risk in the control group** (e.g., the median control group risk across studies) is provided in footnotes. The **risk in the intervention group** (and its 95% confidence interval) is based on the assumed risk in the comparison group and the **relative effect** of the intervention (and its 95% CI).

[1] High risk of bias due to unmentioned allocation concealment and insufficient blind method implementation.

[2] The funnel chart is asymmetric, suggesting publication bias.

[3] Serious unexplained inconsistency (large heterogeneity I^2 = 85%, *P*-value ($P < 0.001$), point estimates, and confidence intervals vary considerably).

[4] Serious unexplained inconsistency (large heterogeneity I^2 = 99%, *P*-value ($P < 0.001$), point estimates, and confidence intervals vary considerably).

[5] Imprecision due to the confidence interval crossing the invalid line zero.

[6] Serious unexplained inconsistency (large heterogeneity I^2 = 83%, *P*-value ($P < 0.001$), point estimates, and confidence intervals vary considerably).

[40] A meta-analysis of adding Shexiang Baoxin Pills in treating patients with chronic heart failure (B. Jin, B.W. Wu, X.Y. Zhuang, X.P. Luo, Y. Li, and H.M. Shi)

Patient or population: chronic heart failure (CHF)
Settings: inpatients and outpatient
Intervention: Shexiang Baoxin Pill + CT
Comparison: CT

**Outcomes,** no of participants (studies)	**Relative effect** (95% CI)	**Anticipated absolute effect* (95% CI)** Control group (CT)	Test group (Shexiang Baoxin Pill + CT)	Difference	**Quality of the evidence (GRADE)**	**What happens**
**Clinical comprehensive efficacy, 1560 (19 studies)**	**RR = 1.18** (1.13 to 1.24)	**598 per 776 (77.1%)**	**715 per 784 (91.2%)** (87.1% to 95.6%)	**14.1% higher** (10.0% to 18.5% higher)	⊕⊕⊖⊖ **moderate due to risk of bias[1] and publication bias[2]**	May improve clinical comprehensive efficacy.
**Outcomes,** no of participants (studies)	**Relative effect** (95% CI)	**Anticipated absolute effect (95% CI)** Control group (CT)	**Test group** (Shexiang Baoxin Pill + CT)	**Difference**	**Quality of the evidence (GRADE)**	**What happens**
**6-minute walk test (6-MWT), 1413(16 studies)**	—	—	—	**41.88 meters more** (33.39 to 50.37 more)	⊕⊖⊖⊖ **very low due to risk of bias[1], publication bias[2] and inconsistency[3]**	Uncertain to increase 6-minute walk test distance.
**Outcomes,** no of participants (studies)	**Relative effect** (95% CI)	**Anticipated absolute effect (95% CI)** Control group (CT)	**Test group** (Shexiang Baoxin Pill + CT)	**Difference**	**Quality of the evidence (GRADE)**	**What happens**
**Brain natriuretic peptide (BNP), 748 (8**	—	—	—	**142.64 pg/mL less** (56.40 to 228.88 less)	⊕⊕⊖⊖ **low due to risk of bias[1],**	May decrease the level of BNP.

Outcomes, no of participants (studies)	Relative effect (95% CI)	Anticipated absolute effect (95% CI)			Quality of the evidence (GRADE)	What happens
studies)					and inconsistency[4]	
**Outcomes,** no of participants (studies)	**Relative effect** (95% CI)	**Anticipated absolute effect (95% CI)**			**Quality of the evidence (GRADE)**	**What happens**
		**Control group** (CT)	**Test group** (Shexiang Baoxin Pill + CT)	**Difference**		
**Left ventricular ejection fraction (LVEF), 2596 (31 studies)**	—	—	—	**4.76% more** (3.64% to 5.87% more)	⊕⊖⊖⊖ **very low due to risk of bias[1], publication bias[2] and inconsistency[5]**	Uncertain to decrease LVEF.
**Outcomes,** no of participants (studies)	**Relative effect** (95% CI)	**Anticipated absolute effect (95% CI)**			**Quality of the evidence (GRADE)**	**What happens**
		**Control group** (CT)	**Test group** (Shexiang Baoxin Pill + CT)	**Difference**		
**Left ventricular end-diastolic diameter (LVEDD), 1186 (15 studies)**	—	—	—	**2.90 mm less** (1.39 to 4.42 less)	⊕⊖⊖⊖ **very low due to risk of bias[1], publication bias[2] and inconsistency[6]**	Uncertain to decrease LVEDD.
**Outcomes,** no of participants (studies)	**Relative effect** (95% CI)	**Anticipated absolute effect (95% CI)**			**Quality of the evidence (GRADE)**	**What happens**
		**Control group** (CT)	**Test group** (Shexiang Baoxin Pill + CT)	**Difference**		
**Left ventricular end-systolic diameter (LVESD), 491 (7 studies)**	—	—	—	**2.22 mm less** (1.25 to 3.19 less)	⊕⊕⊕⊖ **low due to risk of bias[1]**	Probably to decrease LVESD.

Abbreviations: CT, conventional Treatment; CI, confidence interval; OR: odds ratio; GRADE, Grading of Recommendations Assessment, Development and Evaluation.

* The basis for the **risk in the control group** (e.g., the median control group risk across studies) is provided in footnotes. The **risk in the intervention group** (and its 95% confidence interval) is based on the assumed risk in the comparison group and the **relative effect** of the intervention (and its 95% CI).

[1] High risk of bias due to unmentioned allocation concealment and insufficient blind method implementation.

[2] The funnel chart is asymmetric, suggesting publication bias.

[3] Serious unexplained inconsistency (large heterogeneity I^2 = 46.8%, *P*-value (*P* = 0.02), point estimates, and confidence intervals vary considerably).

[4] Serious unexplained inconsistency (large heterogeneity, *P*-value (*P* < 0.01), point estimates, and confidence intervals vary considerably).

[5] Serious unexplained inconsistency (large heterogeneity I^2 = 90.5%, *P*-value (*P* < 0.00001), point estimates, and confidence intervals vary considerably).

[6] Serious unexplained inconsistency (large heterogeneity I^2 = 84.2%, *P*-value (*P* < 0.00001), point estimates, and confidence intervals vary considerably).

[41] Shexiang Baoxin Pills as an adjuvant treatment for chronic heart failure: a system review and meta-analysis (T. Dong, J. Wang, X. Ma et al.)

Patient or population: chronic heart failure (CHF)
Settings: inpatients and outpatient
Intervention: Shexiang Baoxin Pill (22.5~67.5mg, tid) + CT
Comparison: CT / CT + placebo

**Outcomes**, no of participants (studies)	**Relative effect** (95% CI)	**Anticipated absolute effect* (95% CI)**			**Quality of the evidence (GRADE)**	**What happens**
		**Control group** (CT / CT + placebo)	**Test group** (Shexiang Baoxin Pill + CT)	**Difference**		
**Clinical comprehensive efficacy, 1621 (17 studies)**	**OR = 3.88** (2.87 to 5.26)	**609 per 808 (75.4%)**	**749 per 813 (92.1%)**	**16.7% higher**	⊕⊕⊖⊖ **low due to risk of bias[1] and publication bias[2]**	May improve clinical comprehensive efficacy.

**Outcomes**, no of participants (studies)	**Relative effect** (95% CI)	**Anticipated absolute effect (95% CI)**			**Quality of the evidence (GRADE)**	**What happens**
		**Control group** (CT / CT + placebo)	**Test group** (Shexiang Baoxin Pill + CT)	**Difference**		
**6-minute walk test (6-MWT), 1439(15 studies)**	—	—	—	**40.15 meters more** (30.40 to 49.91 more)	⊕⊕⊖⊖ **low due to risk of bias[1] and inconsistency[3]**	May increase 6-minute walk test distance.

**Outcomes**, no of participants (studies)	**Relative effect** (95% CI)	**Anticipated absolute effect (95% CI)**			**Quality of the evidence (GRADE)**	**What happens**
		**Control group** (CT / CT + placebo)	**Test group** (Shexiang Baoxin Pill + CT)	**Difference**		
**Brain natriuretic peptide (BNP), 390 (4 studies)**	—	—	—	**66.95 pg/mL less** (25.34 less to 108.57 more)	⊕⊖⊖⊖ **very low due to risk of bias[1],inconsistency[4] and**	It is uncertain that there is little or no difference in the level of BNP.

					**imprecision**[5]	

**Outcomes,** no of participants (studies)	**Relative effect** (95% CI)	**Anticipated absolute effect (95% CI)**			**Quality of the evidence (GRADE)**	**What happens**
		**Control group** (CT / CT + placebo)	**Test group** (Shexiang Baoxin Pill + CT)	**Difference**		
**Left ventricular ejection fraction (LVEF), 1913 (21 studies)**	—	—	—	**3.89% more** (2.70% to 5.07% more)	⊕⊕⊖⊖ **low due to risk of bias**[1] **and inconsistency**[6]	May increase LVEF.
**Outcomes,** no of participants (studies)	**Relative effect** (95% CI)	**Anticipated absolute effect (95% CI)**			**Quality of the evidence (GRADE)**	**What happens**
		**Control group** (CT / CT + placebo)	**Test group** (Shexiang Baoxin Pill + CT)	**Difference**		
**Cardiac output (CO), 704 (7 studies)**	—	—	—	**0.84 L/min more** (0.68 to 0.99 more)	⊕⊕⊖⊖ **low due to risk of bias**[1] **and inconsistency**[7]	May increase CO.
**Outcomes,** no of participants (studies)	**Relative effect** (95% CI)	**Anticipated absolute effect (95% CI)**			**Quality of the evidence (GRADE)**	**What happens**
		**Control group** (CT / CT + placebo)	**Test group** (Shexiang Baoxin Pill + CT)	**Difference**		
**Stroke volume (SV), 430 (3 studies)**	—	—	—	**7.43 ml more** (4.42 to 10.44 more)	⊕⊕⊖⊖ **low due to risk of bias**[1] **and inconsistency**[8]	May increase SV.

Abbreviations: CT, conventional Treatment; CI, confidence interval; OR: odds ratio; GRADE, Grading of Recommendations Assessment, Development and Evaluation.

***** The basis for the **risk in the control group** (e.g., the median control group risk across studies) is provided in footnotes. The **risk in the intervention group** (and its 95% confidence interval) is based on the assumed risk in the comparison group and the **relative effect** of the intervention (and its 95% CI).

[1] High risk of bias due to unmentioned allocation concealment and insufficient blind method implementation.

[2] The funnel chart is asymmetric, suggesting publication bias.

[3] Serious unexplained inconsistency (large heterogeneity I^2 = 79%, P-value ($P < 0.00001$), point estimates, and confidence intervals vary considerably).

[4] Serious unexplained inconsistency (large heterogeneity I^2 = 71%, *P*-value ($P = 0.02$), point estimates, and confidence intervals vary considerably).

[5] Imprecision due to few events.

[6] Serious unexplained inconsistency (large heterogeneity I^2 = 89%, *P*-value ($P < 0.00001$), point estimates, and confidence intervals vary considerably).

[7] Serious unexplained inconsistency (large heterogeneity I^2 = 56%, *P*-value ($P = 0.03$), point estimates, and confidence intervals vary considerably).

[8] Serious unexplained inconsistency (large heterogeneity I^2 = 85%, *P*-value ($P < 0.00001$), point estimates, and confidence intervals vary considerably).

[42] Clinical efficacy of Shexiang Baoxin Pills combining trimetazidine in treatment of ischemic cardiomyopathy and heart failure in elderly patients: a meta-analysis (X.D. Lin, J.N. Wang, J.M. Tang et al.)

Patient or population: heart failure caused by ischemic cardiomyopathy (HF-ICM)

Settings: inpatients and outpatient

Intervention: Shexiang Baoxin Pill + CT + Trimetazidine

Comparison: CT + Trimetazidine

**Outcomes**, no of participants (studies)	**Relative effect** (95% CI)	**Anticipated absolute effect* (95% CI)** **Control group** (CT + Trimetazidine)	**Test group** (Shexiang Baoxin Pill + CT + Trimetazidine)	**Difference**	**Quality of the evidence (GRADE)**	**What happens**
**Clinical comprehensive efficacy, 1186 (12 studies)**	**RR = 1.30** (1.23 to 1.38)	**419 per 592 (70.8%)**	**547 per 594 (92.1%)** (87.1% to 97.7%)	**21.3% higher** (16.3% to 26.9% higher)	⊕⊕⊖⊖ **low due to risk of bias[1] and publication bias[2]**	May improve clinical comprehensive efficacy.

**Outcomes**, no of participants (studies)	**Relative effect** (95% CI)	**Anticipated absolute effect (95% CI)** **Control group** (CT + Trimetazidine)	**Test group** (Shexiang Baoxin Pill + CT + Trimetazidine)	**Difference**	**Quality of the evidence (GRADE)**	**What happens**
**6-minute walk test (6-MWT), 242(2 studies)**	—	—	—	**56.98 meters more** (42.76 to 71.19 more)	⊕⊕⊖⊖ **low due to risk of bias[1] and imprecision[3]**	May increase 6-minute walk test distance.

**Outcomes**, no of participants (studies)	**Relative effect** (95% CI)	**Anticipated absolute effect (95% CI)** **Control group** (CT + Trimetazidine)	**Test group** (Shexiang Baoxin Pill + CT + Trimetazidine)	**Difference**	**Quality of the evidence (GRADE)**	**What happens**
**Left ventricular ejection fraction (LVEF), 716 (7**	**SMD = 1.55** (0.85 to 2.25)	—	—	—	⊕⊕⊖⊖ **low due to risk of bias[1] and inconsistency[4]**	May decrease LVEF.

studies)

**Outcomes,** no of participants (studies)	**Relative effect** (95% CI)	**Anticipated absolute effect (95% CI)**			**Quality of the evidence (GRADE)**	**What happens**
		**Control group** (CT + Trimetazidine)	**Test group** (Shexiang Baoxin Pill + CT + Trimetazidine)	**Difference**		
**Left ventricular end-diastolic diameter (LVEDD), 524 (5 studies)**	**SMD = -1.57** (-1.91 to -1.22)	—	—	—	⊕⊕⊖⊖ **low due to risk of bias[1], and inconsistency[5]**	May decrease LVEDD.

Abbreviations: CT, conventional Treatment; CI, confidence interval; RR, risk ratio; SMD, standardized mean difference; GRADE, Grading of Recommendations Assessment, Development and Evaluation.

* The basis for the **risk in the control group** (e.g., the median control group risk across studies) is provided in footnotes. The **risk in the intervention group** (and its 95% confidence interval) is based on the assumed risk in the comparison group and the **relative effect** of the intervention (and its 95% CI).

[1] High risk of bias due to unmentioned allocation concealment and insufficient blind method implementation.

[2] The funnel chart is asymmetric, suggesting publication bias.

[3] Imprecision due to few events.

[4] Serious unexplained inconsistency (large heterogeneity I^2 = 94%, *P*-value ($P < 0.00001$), point estimates, and confidence intervals vary considerably).

[5] Serious unexplained inconsistency (large heterogeneity I^2 = 66%, *P*-value ($P = 0.02$), point estimates, and confidence intervals vary considerably).

[43] The effects of Wenxin Keli on left ventricular ejection fraction and brain natriuretic peptide in patients with heart failure: a meta-analysis of randomized controlled trials (Y. Chen, X. Xiong, C. Wang et al.)

Patient or population: heart failure (HF)

Settings: inpatients and outpatient

Intervention: Wenxin Keli (6g, tid; 9g, tid) + CT

Comparison: CT

**Outcomes,** no of participants (studies)	**Relative effect** (95% CI)	**Anticipated absolute effect (95% CI)** Control group (CT)	Test group (Shexiang Baoxin Pill + CT)	Difference	**Quality of the evidence (GRADE)**	**What happens**
**Brain natriuretic peptide (BNP), 748 (8 studies)**	**SMD = -4.18(-5.89 to -2.47)**	—	—	—	⊕⊕⊖⊖ **low due to risk of bias[1], and inconsistency[2]**	May decrease the level of BNP.
**Outcomes,** no of participants (studies)	**Relative effect** (95% CI)	**Anticipated absolute effect (95% CI)** **Control group** (CT)	**Test group** (Shexiang Baoxin Pill + CT)	**Difference**	**Quality of the evidence (GRADE)**	**What happens**
**Left ventricular ejection fraction (LVEF), 903 (11 studies)**	—	—	—	**3.52% more** (2.40% to 4.64% more)	⊕⊕⊖⊖ **low due to risk of bias[1], and inconsistency[3]**	May decrease LVEF.

Abbreviations: CT, conventional Treatment; CI, confidence interval; SMD: standardized mean difference; GRADE, Grading of Recommendations Assessment, Development and Evaluation.

* The basis for the **risk in the control group** (e.g., the median control group risk across studies) is provided in footnotes. The **risk in the intervention group** (and its 95% confidence interval) is based on the assumed risk in the comparison group and the **relative effect** of the intervention (and its 95% CI).

[1] High risk of bias due to unmentioned allocation concealment and insufficient blind method implementation.

[2] Serious unexplained inconsistency (large heterogeneity I^2 = 98%, *P*-value ($P < 0.00001$), point estimates, and confidence intervals vary considerably).

[3] Serious unexplained inconsistency (large heterogeneity I^2 = 64%, *P*-value ($P = 0.002$), point estimates, and confidence intervals vary considerably).

[44] Meta-analysis of Wenxin Keli combined with amiodarone in treating heart failure with arrhythmia (Y.Y. Liang and X.B. Yao)

Patient or population: heart failure with arrhythmia
Settings: inpatients and outpatient
Intervention: Wenxin Keli (5g, tid; 9g, tid) + CT + Amiodarone
Comparison: CT + Amiodarone

**Outcomes,** no of participants (studies)	**Relative effect** (95% CI)	**Anticipated absolute effect* (95% CI)**			**Quality of the evidence (GRADE)**	**What happens**
		**Control group** (CT + Amiodarone)	**Test group** (Wenxin Keli + CT + Amiodarone)	**Difference**		
**Clinical comprehensive efficacy, 386 (4 studies)**	**OR = 5.48** (2.59 to 11.61)	**152 per 193 (78.8%)**	**184 per 193 (95.3%)**	**16.5% higher**	⊕⊕⊕⊖ **moderate due to risk of bias[1]**	Probably to improve clinical comprehensive efficacy.
**Outcomes,** no of participants (studies)	**Relative effect** (95% CI)	**Anticipated absolute effect (95% CI)**			**Quality of the evidence (GRADE)**	**What happens**
		**Control group** (CT + Amiodarone)	**Test group** (Wenxin Keli + CT + Amiodarone)	**Difference**		
**Left ventricular ejection fraction (LVEF), 386 (4 studies)**	—	—	—	**7.56% more** (6.65% to 6.47% more)	⊕⊕⊖⊖ **low due to risk of bias[1] and imprecision[2]**	May decrease LVEF.

Abbreviations: CT, conventional Treatment; CI, confidence interval; OR: odds ratio; GRADE, Grading of Recommendations Assessment, Development and Evaluation.

* The basis for the **risk in the control group** (e.g., the median control group risk across studies) is provided in footnotes. The **risk in the intervention group** (and its 95% confidence interval) is based on the assumed risk in the comparison group and the **relative effect** of the intervention (and its 95% CI).

[1] High risk of bias due to unmentioned allocation concealment and insufficient blind method implementation.

[2] Imprecision due to few events.

[45] A meta-analysis on the effect of Tongxinluo Capsules in treating chronic heart failure (Author: H.T. Liu, X.C. Liu, and M. Li)

Patient or population: chronic heart failure (CHF)
Settings: inpatients and outpatient
Intervention: Tongxinluo Capsule (2~4#, tid) + CT
Comparison: CT

**Outcomes**, no of participants (studies)	**Relative effect** (95% CI)	**Anticipated absolute effect* (95% CI)**			**Quality of the evidence (GRADE)**	**What happens**
		**Control group** (CT)	**Test group** (Tongxinluo Capsule + CT)	**Difference**		
**Mortality (follow-up: 1 year), 77 (1 study)**	—	**7 per 38 (18.42%)**	**1 per 39 (2.56%)**	**15.86% lower**	⊕⊕⊖⊖ **low due to risk of bias[1] and imprecision[2]**	May decrease mortality when the follow-up period is 1 year.
**Outcomes**, no of participants (studies)	**Relative effect** (95% CI)	**Anticipated absolute effect* (95% CI)**			**Quality of the evidence (GRADE)**	**What happens**
		**Control group** (CT)	**Test group** (Tongxinluo Capsule + CT)	**Difference**		
**Hospitalization rate (follow-up: 1 year), 77 (1 study)**	—	**12 per 38 (31.58%)**	**5 per 39 (12.82%)**	**18.76% lower**	⊕⊕⊖⊖ **low due to risk of bias[1] and imprecision[2]**	May decrease hospitalization rate when the follow-up period is 1 year.
**Outcomes**, no of participants (studies)	**Relative effect** (95% CI)	**Anticipated absolute effect* (95% CI)**			**Quality of the evidence (GRADE)**	**What happens**
		**Control group** (CT)	**Test group** (Tongxinluo Capsule + CT)	**Difference**		

**Clinical comprehensive efficacy, 915 (10 studies)**	**OR = 2.76** (1.93 to 3.95)	**324 per 448 (72.3%)**	**406 per 467 (86.9%)**	**14.6% higher**	⊕⊕⊖⊖ **low due to risk of bias[1] and publication bias[2]**	May improve clinical comprehensive efficacy.
**Outcomes,** no of participants (studies)	**Relative effect** (95% CI)	**Anticipated absolute effect* (95% CI)**			**Quality of the evidence (GRADE)**	**What happens**
		**Control group** (CT)	**Test group** (Tongxinluo Capsule + CT)	**Difference**		
**Left ventricular ejection fraction (LVEF), 403(5 studies)**	—	—	—	**5.28% more** (2.57% to 7.99% more)	⊕⊕⊖⊖ **low due to risk of bias[1] and inconsistency[3]**	May increase LVEF.
**Outcomes,** no of participants (studies)	**Relative effect** (95% CI)	**Anticipated absolute effect (95% CI)**			**Quality of the evidence (GRADE)**	**What happens**
		**Control group** (CT)	**Test group** (Tongxinluo Capsule + CT)	**Difference**		
**Left ventricular end-diastolic diameter (LVEDD), 228 (3 studies)**	—	—	—	**2.19 mm less** (0.86 to 3.52 less)	⊕⊕⊖⊖ **low due to risk of bias[1] and imprecision[4]**	May increase LVEDD.
**Outcomes,** no of participants (studies)	**Relative effect** (95% CI)	**Anticipated absolute effect (95% CI)**			**Quality of the evidence (GRADE)**	**What happens**
		**Control group** (CT)	**Test group** (Tongxinluo Capsule + CT)	**Difference**		
**E peak deceleration time (DT), 160 (2 studies)**	—	—	—	**22.65 ms less** (0.12 to 45.17 less)	⊕⊖⊖⊖ **very low due to risk of bias[1], imprecision[4] and inconsistency[5]**	Uncertain to decrease the E peak deceleration time (DT).

Abbreviations: CT, conventional Treatment; CI, confidence interval; OR: odds ratio; RD: risk difference; SMD: standardized mean difference; GRADE, Grading of Recommendations Assessment, Development and Evaluation.

***** The basis for the **risk in the control group** (e.g., the median control group risk across studies) is provided in footnotes. The **risk in the intervention group** (and its 95% confidence interval) is based on the assumed risk in the comparison group and the **relative effect** of the intervention (and its 95% CI).

[1] High risk of bias due to unmentioned allocation concealment and insufficient blind method implementation.

[2] The funnel chart is asymmetric, suggesting publication bias.

[3] Serious unexplained inconsistency (large heterogeneity I^2 = 75%, *P*-value (*P* = 0.003), point estimates, and confidence intervals vary considerably).

[4] Imprecision due to few events.

[5] Serious unexplained inconsistency (large heterogeneity I^2 = 89%, *P*-value (*P* = 0.002), point estimates, and confidence intervals vary considerably).

[46] Meta-analysis of Tongxinluo Capsules in treating coronary heart failure (Author: X. He, C.Y. Lu J.Q., Li, N. Jing, and Y.M. Liu)

Patient or population: heart failure caused by coronary heart disease (HF-CHD)

Settings: inpatients and outpatient

Intervention: Tongxinluo Capsule (2~4#, tid) + CT

Comparison: CT

**Outcomes,** no of participants (studies)	**Relative effect** (95% CI)	**Anticipated absolute effect* (95% CI)**			**Quality of the evidence (GRADE)**	**What happens**
		**Control group** (CT)	**Test group** (Tongxinluo Capsule + CT)	**Difference**		
**Clinical comprehensive efficacy, 1632 (16 studies)**	**OR = 4.28** (3.04 to 6.01)	**657 per 830 (79.2%)**	**754 per 802 (94.0%)**	**14.8% higher**	⊕⊕⊕⊖ **moderate due to risk of bias[1]**	Probably to improve clinical comprehensive efficacy.
**Outcomes,** no of participants (studies)	**Relative effect** (95% CI)	**Anticipated absolute effect* (95% CI)**			**Quality of the evidence (GRADE)**	**What happens**
		**Control group** (CT)	**Test group** (Tongxinluo Capsule + CT)	**Difference**		
**Left ventricular ejection fraction (LVEF), 1480(13 studies)**	—	—	—	**6.64% more** (5.97% to 7.31% more)	⊕⊕⊖⊖ **low due to risk of bias[1] and inconsistency[2]**	May increase LVEF.
**Outcomes,** no of participants (studies)	**Relative effect** (95% CI)	**Anticipated absolute effect (95% CI)**			**Quality of the evidence (GRADE)**	**What happens**
		**Control group** (CT)	**Test group** (Tongxinluo Capsule + CT)	**Difference**		
**Cardiac output (CO), 800 (7 studies)**	—	—	—	**0.40 L/min less** (0.32 to 0.47 less)	⊕⊕⊕⊖ **moderate due to risk of bias[1]**	Probably to increase CO.

**Outcomes,** no of participants (studies)	**Relative effect** (95% CI)	**Anticipated absolute effect (95% CI)**			**Quality of the evidence (GRADE)**	**What happens**
		**Control group** (CT)	**Test group** (Tongxinluo Capsule + CT)	**Difference**		
**Adverse events, 718 (6 studies)**	**RD = 0.01** (-0.02 to 0.04)	**14 per 359 (3.90%)**	**16 per 359 (4.46%)**	**0.56% higher**	⊕⊖⊖⊖ **very low due to risk of bias[1] and serious imprecision[3, 4]**	It is uncertain that there is little or no difference in adverse events.

Abbreviations: CT, conventional Treatment; CI, confidence interval; OR: odds ratio; RD: risk difference; SMD: standardized mean difference; GRADE, Grading of Recommendations Assessment, Development and Evaluation.

***** The basis for the **risk in the control group** (e.g., the median control group risk across studies) is provided in footnotes. The **risk in the intervention group** (and its 95% confidence interval) is based on the assumed risk in the comparison group and the **relative effect** of the intervention (and its 95% CI).

[1] High risk of bias due to unmentioned allocation concealment and insufficient blind method implementation.

[2] Serious unexplained inconsistency (large heterogeneity I^2 = 98%, *P*-value ($P < 0.00001$), point estimates, and confidence intervals vary considerably).

[3] Imprecision due to few events.

[4] Imprecision due to the confidence interval crossing the invalid line zero.

[47] Compound Danshen Dripping Pills treatment of the chronic heart failure healing the meta-analysis of curative effect (Author: Q. Wang)

Patient or population: chronic heart failure (CHF)
Settings: inpatients and outpatient
Intervention: Compound Danshen Dripping Pill (10#, tid) + CT
Comparison: CT

**Outcomes,** no of participants (studies)	**Relative effect** (95% CI)	**Anticipated absolute effect* (95% CI)**			**Quality of the evidence (GRADE)**	**What happens**
		**Control group** (CT)	**Test group** (Tongxinluo Capsule + CT)	**Difference**		
**Clinical comprehensive efficacy, 912 (9 studies)**	**RR = 1.22** (1.15 to 1.29)	**344 per 455 (75.6%)**	**421 per 457 (92.1%)** (86.9% to 97.5%)	**16.5% higher** (11.3% to 21.9%)	⊕⊕⊖⊖ **moderate due to risk of bias[1] and publication bias[2]**	May improve clinical comprehensive efficacy.

Abbreviations: CT, conventional Treatment; CI, confidence interval; RR, relative risk; GRADE, Grading of Recommendations Assessment, Development and Evaluation.

* The basis for the **risk in the control group** (e.g., the median control group risk across studies) is provided in footnotes. The **risk in the intervention group** (and its 95% confidence interval) is based on the assumed risk in the comparison group and the **relative effect** of the intervention (and its 95% CI).

[1] High risk of bias due to unmentioned allocation concealment and insufficient blind method implementation.

[2] The funnel chart is asymmetric, suggesting publication bias.

[48] Systematic reviews on curative effect of Compound Danshen Dripping Pills combined with routine western medicine for heart failure (Author: R.K. Lai, L. Liao, and G.M. Pan)

Patient or population: chronic heart failure (CHF)
Settings: inpatients and outpatient
Intervention: Compound Danshen Dripping Pill + CT
Comparison: CT

**Outcomes,** no of participants (studies)	**Relative effect** (95% CI)	**Anticipated absolute effect (95% CI)** **Control group** (CT)	**Test group** (Compound Danshen Dripping Pill + CT)	**Difference**	**Quality of the evidence (GRADE)**	**What happens**
**Clinical comprehensive efficacy, 1440 (17 studies)**	**RR = 1.21** (1.16 to 1.27)	**544 per 712 (76.4%)**	**675 per 728 (92.7%)** (88.6% to 97.0%)	**16.3% higher** (12.2% to 20.6% higher)	⊕⊕⊖⊖ **low due to risk of bias[1] and publication bias[2]**	May improve the clinical comprehensive efficacy.

**Outcomes,** no of participants (studies)	**Relative effect** (95% CI)	**Anticipated absolute effect (95% CI)** **Control group** (CT)	**Test group** (Compound Danshen Dripping Pill + CT)	**Difference**	**Quality of the evidence (GRADE)**	**What happens**
**6-minute walk test (6-MWT), 347 (4 studies)**	—	—	—	**44.89 meters more** (32.59 to 57.18 more)	⊕⊕⊖⊖ **low due to risk of bias[1] and imprecision[3]**	May increase the 6-minute walk test distance.

**Outcomes,** no of participants (studies)	**Relative effect** (95% CI)	**Anticipated absolute effect (95% CI)** **Control group** (CT)	**Test group** (Compound Danshen Dripping Pill + CT)	**Difference**	**Quality of the evidence (GRADE)**	**What happens**
**Brain natriuretic peptide (BNP), 369 (4 studies)**	—	—	—	**197.84 pg/mL less** (154.33 to 241.35 less)	⊕⊖⊖⊖ **very low due to risk of bias[1], imprecision[3] and**	May decrease the level of BNP.

					**inconsistency[4]**	
**Outcomes,** no of participants (studies)	**Relative effect** (95% CI)	**Anticipated absolute effect (95% CI)** **Control group** (CT)	**Test group** (Compound Danshen Dripping Pill + CT)	**Difference**	**Quality of the evidence (GRADE)**	**What happens**
**Left ventricular ejection fraction (LVEF), 876 (10 studies)**	—	—	—	**4.67% more** (3.31% to 6.02% more)	⊕⊕⊖⊖ **low due to risk of bias[1] and inconsistency[5]**	May increase the LVEF.
**Outcomes,** no of participants (studies)	**Relative effect** (95% CI)	**Anticipated absolute effect (95% CI)** **Control group** (CT)	**Test group** (Compound Danshen Dripping Pill + CT)	**Difference**	**Quality of the evidence (GRADE)**	**What happens**
**Left ventricular end-diastolic diameter (LVEDD), 440 (5 studies)**	—	—	—	**2.99 mm less** (4.22 to 1.75 less)	⊕⊕⊕⊖ **moderate due to risk of bias[1]**	Probably to decrease the LVEDD.
**Outcomes,** no of participants (studies)	**Relative effect** (95% CI)	**Anticipated absolute effect (95% CI)** **Control group** (CT)	**Test group** (Compound Danshen Dripping Pill + CT)	**Difference**	**Quality of the evidence (GRADE)**	**What happens**
**Left ventricular end-systolic diameter (LVESD), 184 (2 studies)**	—	—	—	**3.47 mm less** (5.22 to 1.72 less)	⊕⊕⊖⊖ **low due to risk of bias[1] and imprecision[3]**	May decrease the LVESD.
**Outcomes,** no of participants (studies)	**Relative effect** (95% CI)	**Anticipated absolute effect (95% CI)** **Control group** (CT / CT + placebo)	**Test group** (Compound Danshen Dripping Pill + CT)	**Difference**	**Quality of the evidence (GRADE)**	**What happens**
**Stroke volume (SV), 170 (3 studies)**	—	—	—	**4.40 ml more** (1.95 to 6.84 more)	⊕⊖⊖⊖ **very low due to risk of bias[1], imprecision[3] and**	Uncertain to increase SV.

					**inconsistency**[6]	
**Outcomes,** no of participants (studies)	**Relative effect** (95% CI)	**Anticipated absolute effect (95% CI)** **Control group** (CT)	**Test group** (Compound Danshen Dripping Pill + CT)	**Difference**	**Quality of the evidence (GRADE)**	**What happens**
**Interventricular septal thickness at diastole (IVSd), 184 (2 studies)**	—	—	—	**0.66 mm more** (0.35 to 0.97 more)	⊕⊕⊖⊖ **low due to risk of bias**[1] **and imprecision**[3]	May decrease IVSd.
**Outcomes,** no of participants (studies)	**Relative effect** (95% CI)	**Anticipated absolute effect (95% CI)** **Control group** (CT)	**Test group** (Compound Danshen Dripping Pill + CT)	**Difference**	**Quality of the evidence (GRADE)**	**What happens**
**Left ventricular posterior wall defect (LVPWd), 184 (2 studies)**	—	—	—	**0.68 mm more** (0.35 to 1.00 more)	⊕⊕⊖⊖ **low due to risk of bias**[1] **and imprecision**[3]	May decrease the LVESd.
**Outcomes,** no of participants (studies)	**Relative effect** (95% CI)	**Anticipated absolute effect (95% CI)** **Control group** (CT)	**Test group** (Compound Danshen Dripping Pill + CT)	**Difference**	**Quality of the evidence (GRADE)**	**What happens**
**Adverse events, 4846 (56 studies), 184 (2 studies)**	**RR = 1.25 (0.57, 2.75)**	**9 per 149 (6.04%)**	**12 per 156 (7.69%)**	**1.65% higher**	⊕⊖⊖⊖ **low due to risk of bias**[1] **and serious imprecision**[3, 7]	It is uncertain that there is little or no difference in adverse events.

Abbreviations: CT, conventional treatment; CI, confidence interval; RR, risk ratio; GRADE, Grading of Recommendations Assessment, Development and Evaluation.

* The basis for the **risk in the control group** (e.g., the median control group risk across studies) is provided in footnotes. The **risk in the intervention group** (and its 95% confidence interval) is based on the assumed risk in the comparison group and the **relative effect** of the intervention (and its 95% CI).

[1] High risk of bias due to unmentioned allocation concealment and insufficient blind method implementation.

[2] The funnel chart is asymmetric, suggesting publication bias.

[3] Imprecision due to few events.

[4] Serious unexplained inconsistency (large heterogeneity I^2 = 80%, *P*-value (P = 0.002), point estimates, and confidence intervals vary considerably).

[5] Serious unexplained inconsistency (large heterogeneity I^2 = 66%, *P*-value (P = 0.001), point estimates, and confidence intervals vary considerably).

[6] Serious unexplained inconsistency (large heterogeneity I^2 = 58%, point estimates, and confidence intervals vary considerably).

[7] Imprecision due to the too wide confidence interval and the confidence interval crossing the invalid line zero.

[49] Efficacy of Zhenyuan Capsules as adjuvant therapy for chronic heart failure: a Meta-analysis (Author: C.J. Wu and T.S. Tang)

Patient or population: chronic heart failure (CHF)
Settings: inpatients and outpatient
Intervention: Zhenyuan Capsule + CT
Comparison: CT

**Outcomes**, no of participants (studies)	**Relative effect** (95% CI)	**Anticipated absolute effect (95% CI)**			**Quality of the evidence (GRADE)**	**What happens**
		**Control group** (CT)	**Test group** (Zhenyuan Capsule + CT)	**Difference**		
**Clinical comprehensive efficacy, 901 (10 studies)**	**OR = 4.35** (2.97 to 6.36)	**311 per 446 (69.7%)**	**413 per 455 (90.8%)**	**21.1% higher**	⊕⊕⊖⊖ **low due to risk of bias[1] and publication bias[2]**	May improve clinical comprehensive efficacy.
**Outcomes**, no of participants (studies)	**Relative effect** (95% CI)	**Anticipated absolute effect (95% CI)**			**Quality of the evidence (GRADE)**	**What happens**
		**Control group** (CT)	**Test group** (Zhenyuan Capsule + CT)	**Difference**		
**N-terminal pro-brain natriuretic peptide (NT-proBNP), 270 (3 studies)**	—	—	—	**572.24 pg/mL less** (427.16 to 717.32 less)	⊕⊕⊖⊖ **low due to risk of bias[1] and imprecision[3]**	May decrease the level of NT-proBNP.
**Outcomes**, no of participants (studies)	**Relative effect** (95% CI)	**Anticipated absolute effect (95% CI)**			**Quality of the evidence (GRADE)**	**What happens**
		**Control group** (CT)	**Test group** (Zhenyuan Capsule + CT)	**Difference**		
**Left ventricular ejection fraction (LVEF), 851 (9 studies)**	—	—	—	**7.89% more** (4.24% to 11.54% more)	⊕⊕⊖⊖ **low due to risk of bias[1] and inconsistency[4]**	May increase LVEF.
**Outcomes**, no of participants (studies)	**Relative effect** (95% CI)	**Anticipated absolute effect (95% CI)**			**Quality of the evidence (GRADE)**	**What happens**
		**Control group**	**Test group**	**Difference**		

		(CT)	(Zhenyuan Capsule + CT)			
**Cardiac output (CO), 434 (4 studies)**	—	—	—	**0.79 L/min more** (0.61 to 0.98 more)	⊕⊕⊖⊖ **low due to risk of bias[1] and inconsistency[5]**	May increase CO.

Abbreviations: CT, conventional treatment; CI, confidence interval; RR, risk ratio; GRADE, Grading of Recommendations Assessment, Development and Evaluation.

***** The basis for the **risk in the control group** (e.g., the median control group risk across studies) is provided in footnotes. The **risk in the intervention group** (and its 95% confidence interval) is based on the assumed risk in the comparison group and the **relative effect** of the intervention (and its 95% CI).

[1] High risk of bias due to unmentioned allocation concealment and insufficient blind method implementation.

[2] The funnel chart is asymmetric, suggesting publication bias.

[3] Imprecision due to few events.

[4] Serious unexplained inconsistency (large heterogeneity I^2 = 97%, P-value ($P < 0.00001$), point estimates, and confidence intervals vary considerably).

[5] Serious unexplained inconsistency (large heterogeneity I^2 = 88%, P-value ($P < 0.0001$), point estimates, and confidence intervals vary considerably).

[50] Adjuvant effects of Zhenyuan Capsules on chronic heart failure: Meta-analysis (Author: Y. Cao, W.Q. Wang, L. Lu, and X.M. Guo)

Patient or population: chronic heart failure (CHF)

Settings: inpatients and outpatient

Intervention: Zhenyuan Capsule (0.25~1g, tid; 0.5g, bid) + CT

Comparison: CT

**Outcomes,** no of participants (studies)	**Relative effect** (95% CI)	**Anticipated absolute effect* (95% CI)** **Control group** (CT)	**Test group** (Zhenyuan Capsule + CT)	**Difference**	**Quality of the evidence (GRADE)**	**What happens**
**New York Heart Association (NYHA) cardiac function efficacy, 1033 (12 studies)**	**RR = 1.27** (1.20 to 1.35)	**365 per 512 (71.3%)**	**473 per 521 (90.8%)** (85.6% to 96.3%)	**19.5% higher** (14.3% to 25.0% higher)	⊕⊕⊖⊖ **low due to risk of bias[1] and publication bias[2]**	May improve NYHA cardiac function efficacy.
**Outcomes,** no of participants (studies)	**Relative effect** (95% CI)	**Anticipated absolute effect (95% CI)** **Control group** (CT)	**Test group** (Zhenyuan Capsule + CT)	**Difference**	**Quality of the evidence (GRADE)**	**What happens**
**Brain natriuretic peptide (BNP), 441 (5 studies)**	—	—	—	**601.02 pg/mL less** (270. 99 less to 931.05 more)	⊕⊕⊖⊖ **low due to risk of bias[1] and inconsistency[3]**	There may be little or no difference in BNP.
**Outcomes,** no of participants (studies)	**Relative effect** (95% CI)	**Anticipated absolute effect (95% CI)** **Control group** (CT)	**Test group** (Zhenyuan Capsule + CT)	**Difference**	**Quality of the evidence (GRADE)**	**What happens**
**Left ventricular ejection fraction (LVEF), 987 (11 studies)**	—	—	—	**6.78% more** (3. 68% to 9. 88% more)	⊕⊖⊖⊖ **low due to risk of bias[1], publication bias[2] and inconsistency[4]**	Uncertain to increase LVEF.
**Outcomes,** no of	**Relative effect**	**Anticipated absolute effect (95% CI)**			**Quality of the evidence**	**What happens**

Outcomes, no of participants (studies)	Relative effect (95% CI)	Anticipated absolute effect (95% CI) Control group (CT)	Test group (Zhenyuan Capsule + CT)	Difference	Quality of the evidence (GRADE)	What happens
**Cardiac output (CO), 434 (4 studies)**	—	—	—	**0.80 L/min more** (0.55 to 1.06 more)	⊕⊕⊖⊖ **low due to risk of bias[1] and inconsistency[5]**	May increase CO.
**Outcomes,** no of participants (studies)	**Relative effect** (95% CI)	**Anticipated absolute effect (95% CI)** **Control group** (CT)	**Test group** (Zhenyuan Capsule + CT)	**Difference**	**Quality of the evidence (GRADE)**	**What happens**
**Stroke volume (SV), 345 (3 studies)**	—	—	—	**7.62 ml more** (6.39 to 8.84 more)	⊕⊕⊖⊖ **low due to risk of bias[1] and imprecision[6]**	May increase SV.

Abbreviations: CT, conventional Treatment; CI, confidence interval; OR: odds ratio; GRADE, Grading of Recommendations Assessment, Development and Evaluation.

***** The basis for the **risk in the control group** (e.g., the median control group risk across studies) is provided in footnotes. The **risk in the intervention group** (and its 95% confidence interval) is based on the assumed risk in the comparison group and the **relative effect** of the intervention (and its 95% CI).

[1] High risk of bias due to unmentioned allocation concealment and insufficient blind method implementation.

[2] The funnel chart is asymmetric, suggesting publication bias.

[3] Serious unexplained inconsistency (large heterogeneity I^2 = 94%, *P*-value ($P < 0.00001$), point estimates, and confidence intervals vary considerably).

[4] Serious unexplained inconsistency (large heterogeneity I^2 = 95%, *P*-value (P < 0.00001), point estimates, and confidence intervals vary considerably).

[5] Serious unexplained inconsistency (large heterogeneity I^2 = 92%, *P*-value ($P < 0.00001$), point estimates, and confidence intervals vary considerably).

[6] Imprecision due to few events.

[51] A meta-analysis of clinical efficacy and safety of Zhenyuan Capsules on heart failure combined with western medicine

(Author: Y.Q. Chen, Z.D. Huang, and P.F. Zhang)

Patient or population: heart failure (HF)

Settings: inpatients and outpatient

Intervention: Zhenyuan Capsule (0.25~1g, tid) + CT

Comparison: CT

**Outcomes,** no of participants (studies)	**Relative effect** (95% CI)	**Anticipated absolute effect* (95% CI)**			**Quality of the evidence (GRADE)**	**What happens**
		**Control group** (CT)	**Test group** (Zhenyuan Capsule + CT)	**Difference**		
**New York Heart Association (NYHA) cardiac function efficacy, 831 (10 studies)**	**OR = 3.71** (12.531 to 5.445)	**365 per 512 (71.3%)**	**473 per 521 (90.8%)**	**19.5% higher**	⊕⊕⊖⊖ **low due to risk of bias[1] and publication bias[2]**	May improve NYHA cardiac function efficacy.
**Outcomes,** no of participants (studies)	**Relative effect** (95% CI)	**Anticipated absolute effect (95% CI)**			**Quality of the evidence (GRADE)**	**What happens**
		**Control group** (CT)	**Test group** (Zhenyuan Capsule + CT)	**Difference**		
**Left ventricular ejection fraction (LVEF)**						
**LVEF** *course of treatment* < *6 weeks*, 368 (4 studies)	**SMD = 1.38** **(**1.14 to 1.62)	—	—	—	⊕⊖⊖⊖ **very low due to risk of bias[1], imprecision[3] and inconsistency[4]**	Uncertain to increase LVEF.
**LVEF** *course of treatment* ≥ *6 weeks*, 469 (6 studies)	**SMD = 0.85** **(**0.66 to 1.04)	—	—	—	⊕⊕⊕⊖ **moderate due to risk of bias[1]**	Probably to increase LVEF.

Abbreviations: CT, conventional Treatment; CI, confidence interval; OR: odds ratio; SMD, standardized mean difference; GRADE, Grading of Recommendations Assessment, Development and Evaluation.

* The basis for the **risk in the control group** (e.g., the median control group risk across studies) is provided in footnotes. The **risk in the intervention group** (and its 95% confidence

interval) is based on the assumed risk in the comparison group and the **relative effect** of the intervention (and its 95% CI).

1 High risk of bias due to unmentioned allocation concealment and insufficient blind method implementation.

2 The funnel chart is asymmetric, suggesting publication bias.

3 Imprecision due to few events.

4 Serious unexplained inconsistency (large heterogeneity I^2 = 95.3%, *P*-value ($P < 0.001$), point estimates, and confidence intervals vary considerably).

[52] Meta-analysis of Buyi Qiangxin Tablets for Chronic Heart Failure (Author: J.T. Li, J. Lu, C.Y. Liu, Y. Pang, and J.Q. Lu)

Patient or population: chronic heart failure (CHF)

Settings: inpatients and outpatient

Intervention: Buyi Qiangxin Tablet (4#, tid) + CT

Comparison: CT

**Outcomes,** no of participants (studies)	**Relative effect** (95% CI)	**Anticipated absolute effect (95% CI)**<br>**Control group** (CT)	**Test group** (Buyi Qiangxin Tablet + CT)	**Difference**	**Quality of the evidence (GRADE)**	**What happens**
**Clinical comprehensive efficacy, 334 (5 studies)**	**OR = 4.54** (2.23 to 9.26)	**127 per 167 (76.0%)**	**156 per 167 (93.4%)**	**17.4% higher**	⊕⊕⊕⊖ **moderate due to risk of bias**[1]	Probably to improve clinical comprehensive efficacy.

**Outcomes,** no of participants (studies)	**Relative effect** (95% CI)	**Anticipated absolute effect* (95% CI)**<br>**Control group** (CT)	**Test group** (Zhenyuan Capsule + CT)	**Difference**	**Quality of the evidence (GRADE)**	**What happens**
**New York Heart Association (NYHA) cardiac function efficacy, 128 (2 studies)**	**OR = 4.41** (1.63 to 11.93)	**44 per 64 (68.8%)**	**58 per 64(90.6%)**	**21.8% higher**	⊕⊕⊕⊖ **moderate due to risk of bias**[1]	Probably to improve NYHA cardiac function efficacy.

**Outcomes,** no of participants (studies)	**Relative effect** (95% CI)	**Anticipated absolute effect (95% CI)**<br>**Control group** (CT)	**Test group** (Buyi Qiangxin Tablet + CT)	**Difference**	**Quality of the evidence (GRADE)**	**What happens**
**Minnesota Living with Heart Failure Questionnaire (MLHFQ) score, 188 (3 studies)**	—	—	—	**11.23 points lower** (8.01 to 14.45 lower)	⊕⊕⊖⊖ **low due to risk of bias**[1] **and imprecision**[2]	May decrease MLHFQ score.

**Outcomes,** no of participants (studies)	**Relative effect** (95% CI)	**Anticipated absolute effect (95% CI)**			**Quality of the evidence (GRADE)**	**What happens**
		**Control group** (CT)	**Test group** (Buyi Qiangxin Tablet + CT)	**Difference**		
**N-terminal pro-brain natriuretic peptide (NT-proBNP), 214 (3 studies)**	**SMD = -1.58** (-1.88 to -1.282)	—	—	—	⊕⊖⊖⊖ **very low due to risk of bias[1], imprecision[2] and inconsistency[3]**	Uncertain to decrease the level of NT-proBNP.
**Outcomes,** no of participants (studies)	**Relative effect** (95% CI)	**Anticipated absolute effect (95% CI)**			**Quality of the evidence (GRADE)**	**What happens**
		**Control group** (CT)	**Test group** (Buyi Qiangxin Tablet + CT)	**Difference**		
**Left ventricular ejection fraction (LVEF), 433 (6 studies)**	—	—	—	**3.88% more** (3.18% to 4.59% more)	⊕⊕⊕⊖ **moderate due to risk of bias[1]**	Probably to increase LVEF.

Abbreviations: CT, conventional treatment; CI, confidence interval; RR, risk ratio; GRADE, Grading of Recommendations Assessment, Development and Evaluation.

***** The basis for the **risk in the control group** (e.g., the median control group risk across studies) is provided in footnotes. The **risk in the intervention group** (and its 95% confidence interval) is based on the assumed risk in the comparison group and the **relative effect** of the intervention (and its 95% CI).

[1] High risk of bias due to unmentioned allocation concealment and insufficient blind method implementation.

[2] Imprecision due to few events.

[3] Serious unexplained inconsistency (large heterogeneity I^2 = 93%, *P*-value ($P < 0.01$), point estimates, and confidence intervals vary considerably).

[53] A meta-analysis on randomized controlled trials of routine western medical treatment plus Buyi Qiangxin Tablets for treating chronic heart failure (Author: X.Y. Mo, X.L. Wang, Y.Z. Hou, Y.F. Bi, and J.Y. Mao)

Patient or population: chronic heart failure (CHF)

Settings: inpatients and outpatient

Intervention: Buyi Qiangxin Tablet (4#, tid) + CT

Comparison: CT

**Outcomes,** no of participants (studies)	**Relative effect** (95% CI)	**Anticipated absolute effect* (95% CI)** **Control group** (CT)	**Test group** (Zhenyuan Capsule + CT)	**Difference**	**Quality of the evidence (GRADE)**	**What happens**
**New York Heart Association (NYHA) cardiac function efficacy, 188 (3 studies)**	**RR = 1.25** (1.09 to 1.43)	**69 per 94 (73.4%)**	**86 per 94(91.5%)** (80.0% to 105.0%)	**18.1% higher** (6.6% to 31.6%)	⊕⊕⊕⊖ **moderate due to risk of bias[1]**	Probably to improve NYHA cardiac function efficacy.
**Outcomes,** no of participants (studies)	**Relative effect** (95% CI)	**Anticipated absolute effect (95% CI)** **Control group** (CT)	**Test group** (Buyi Qiangxin Tablet + CT)	**Difference**	**Quality of the evidence (GRADE)**	**What happens**
**Minnesota Living with Heart Failure Questionnaire (MLHFQ) score, 338 (4 studies)**	—	—	—	**10.90 points lower** (8.68 to 13.12 lower)	⊕⊕⊖⊖ **low due to risk of bias[1] and imprecision[2]**	May decrease MLHFQ score.
**Outcomes,** no of participants (studies)	**Relative effect** (95% CI)	**Anticipated absolute effect (95% CI)** **Control group** (CT)	**Test group** (Buyi Qiangxin Tablet + CT)	**Difference**	**Quality of the evidence (GRADE)**	**What happens**
**N-terminal pro-brain natriuretic peptide (NT-proBNP), 393 (4**	**SMD = -2.02** (-3.02 to -1.02)	—	—	—	⊕⊖⊖⊖ **moderate due to risk of bias[1], imprecision[2] and**	Uncertain to increase LVEF.

**studies)**					**inconsistency[3]**	

**Outcomes,** no of participants (studies)	**Relative effect** (95% CI)	**Anticipated absolute effect (95% CI)**			**Quality of the evidence (GRADE)**	**What happens**
		**Control group** (CT)	**Test group** (Buyi Qiangxin Tablet + CT)	**Difference**		
**Left ventricular ejection fraction (LVEF), 573 (7 studies)**	—	—	—	**4.31% more** (3.73% to 4.90% more)	⊕⊕⊕⊖ **moderate due to risk of bias[1]**	Probably to increase LVEF.

**Outcomes,** no of participants (studies)	**Relative effect** (95% CI)	**Anticipated absolute effect (95% CI)**			**Quality of the evidence (GRADE)**	**What happens**
		**Control group** (CT)	**Test group** (Buyi Qiangxin Tablet + CT)	**Difference**		
**Lee's Heart Failure Score, 304 (3 studies)**	—	—	—	**1.09% less** (0.83% to 1.36% less)	⊕⊕⊖⊖ **low due to risk of bias[1] and imprecision[2]**	May decrease Lee's Heart Failure Score.

Abbreviations: CT, conventional treatment; CI, confidence interval; RR, risk ratio; GRADE, Grading of Recommendations Assessment, Development and Evaluation.

***** The basis for the **risk in the control group** (e.g., the median control group risk across studies) is provided in footnotes. The **risk in the intervention group** (and its 95% confidence interval) is based on the assumed risk in the comparison group and the **relative effect** of the intervention (and its 95% CI).

[1] High risk of bias due to unmentioned allocation concealment and insufficient blind method implementation.

[2] Imprecision due to few events.

[3] Serious unexplained inconsistency (large heterogeneity $I^2 = 94\%$, *P*-value ($P < 0.00001$), point estimates, and confidence intervals vary considerably).

[54] Efficacy and safety of Yangxinshi Tablets in the treatment of chronic heart failure: a meta-analysis (Author: Q.Y. Chen and X.H. Dai)

Patient or population: chronic heart failure (CHF)
Settings: inpatients and outpatient
Intervention: Yangxinshi Tablet (0.9~1.2g, tid) + CT
Comparison: CT

**Outcomes,** no of participants (studies)	**Relative effect** (95% CI)	**Anticipated absolute effect (95% CI)**			**Quality of the evidence (GRADE)**	**What happens**
		**Control group** (CT)	**Test group** (Yangxinshi Tablet + CT)	**Difference**		
**Clinical comprehensive efficacy, 1232 (12 studies)**	**OR = 3.24** (2.33 to 4.49)	**460 per 611 (75.3%)**	**572 per 632 (90.5%)**	**15.2% higher**	⊕⊕⊕⊖ **moderate due to risk of bias[1]**	Probably to improve clinical comprehensive efficacy.
**Outcomes,** no of participants (studies)	**Relative effect** (95% CI)	**Anticipated absolute effect (95% CI)**			**Quality of the evidence (GRADE)**	**What happens**
		**Control group** (CT)	**Test group** (Yangxinshi Tablet + CT)	**Difference**		
**6-minute walk test (6-MWT), 424 (4 studies)**	—	—	—	**47.21 meters more** (37.45 to 56.97 more)	⊕⊕⊕⊖ **moderate due to risk of bias[1]**	Probably to increase 6-minute walk test distance.
**Outcomes,** no of participants (studies)	**Relative effect** (95% CI)	**Anticipated absolute effect (95% CI)**			**Quality of the evidence (GRADE)**	**What happens**
		**Control group** (CT)	**Test group** (Yangxinshi Tablet + CT)	**Difference**		
**Brain natriuretic peptide (BNP), 197 (2 studies)**	**SMD = -0.21** (-0.84 to 0.42)	—	—	—	⊖⊖⊖⊖ **very low due to risk of bias[1], imprecision[2, 3] and inconsistency[4]**	It is uncertain that there is little or no difference in the level of BNP.
**Outcomes,** no of	**Relative effect**	**Anticipated absolute effect (95% CI)**			**Quality of the evidence**	**What happens**

participants (studies)	(95% CI)	**Control group** (CT)	**Test group** (Yangxinshi Tablet + CT)	**Difference**	**(GRADE)**	
**N-terminal pro-brain natriuretic peptide (NT-proBNP), 176 (2 studies)**	**SMD = -1.57** (-2.50 to -0.63)	—	—	—	⊕⊖⊖⊖ **very low due to risk of bias[1], imprecision[2] and inconsistency[5]**	Uncertain to decrease the level of NT-proBNP.
**Outcomes,** no of participants (studies)	**Relative effect** (95% CI)	**Anticipated absolute effect (95% CI)** **Control group** (CT)	**Test group** (Yangxinshi Tablet + CT)	**Difference**	**Quality of the evidence (GRADE)**	**What happens**
**Left ventricular ejection fraction (LVEF), 818 (8 studies)**	—	—	—	**2.92% more** (1.74% less to 7.59% more)	⊕⊖⊖⊖ **very low due to risk of bias[1], imprecision[3] and inconsistency[6]**	It is uncertain that there is little or no difference in LVEF.
**Outcomes,** no of participants (studies)	**Relative effect** (95% CI)	**Anticipated absolute effect (95% CI)** **Control group** (CT)	**Test group** (Yangxinshi Tablet + CT)	**Difference**	**Quality of the evidence (GRADE)**	**What happens**
**hs-CRP, 154 (2 studies)**	—	—	—	**0.39 mg/L less** (0.80 less to 0.02 more)	⊕⊖⊖⊖ **very low due to risk of bias[1], imprecision[2, 3] and inconsistency[7]**	It is uncertain that there is little or no difference in hs-CRP.

Abbreviations: CT, conventional treatment; CI, confidence interval; RR, risk ratio; GRADE, Grading of Recommendations Assessment, Development and Evaluation.

***** The basis for the **risk in the control group** (e.g., the median control group risk across studies) is provided in footnotes. The **risk in the intervention group** (and its 95% confidence interval) is based on the assumed risk in the comparison group and the **relative effect** of the intervention (and its 95% CI).

[1] High risk of bias due to unmentioned allocation concealment and insufficient blind method implementation.

[2] Imprecision due to few events.

[3] Imprecision due to the confidence interval crossing the invalid line zero.

[4] Serious unexplained inconsistency (large heterogeneity I^2 = 79%, *P*-value (P = 0.03), point estimates, and confidence intervals vary considerably).

[5] Serious unexplained inconsistency (large heterogeneity I^2 = 84%, *P*-value (P = 0.01), point estimates, and confidence intervals vary considerably).

[6] Serious unexplained inconsistency (large heterogeneity I^2 = 97%, *P*-value (P < 0.00001), point estimates, and confidence intervals vary considerably).

[7] Serious unexplained inconsistency (large heterogeneity I^2 = 58%, *P*-value (P = 0.12), point estimates, and confidence intervals vary considerably).

[55] Efficacy of Xuezhikang in the treatment of chronic heart failure: a meta-analysis (Author: H. Zhang, T.Q. Zhang, and J.X. Gu)

Patient or population: chronic heart failure (CHF)
Settings: inpatients and outpatient
Intervention: Xuezhikang (0.3g, bid; 0.6g, qd; 0.6g, bid; 0.6g, tid) + CT
Comparison: CT / CT + placebo

**Outcomes,** no of participants (studies)	**Relative effect** (95% CI)	**Anticipated absolute effect* (95% CI)** **Control group** (CT / CT + placebo)	**Test group** (Xuezhikangs + CT)	**Difference**	**Quality of the evidence (GRADE)**	**What happens**
**Clinical comprehensive efficacy, 382 (5 studies)**	**OR = 3.04** (1.81 to 5.10)	**126 per 185 (68.1%)**	**170 per 197 (86.3%)**	**18.2% higher**	⊕⊕⊕⊖ **moderate due to risk of bias[1]**	Probably to improve clinical comprehensive efficacy.
**Outcomes,** no of participants (studies)	**Relative effect** (95% CI)	**Anticipated absolute effect (95% CI)** **Control group** (CT / CT + placebo)	**Test group** (Xuezhikangs + CT)	**Difference**	**Quality of the evidence (GRADE)**	**What happens**
**Brain natriuretic peptide (BNP), 252 (3 studies)**	—	—	—	**252.25 pg/mL less** (185.70 to 318.80 less)	⊕⊖⊖⊖ **very low due to risk of bias[1], imprecision[2] and inconsistency[3]**	Uncertain to decrease the level of BNP.
**Outcomes,** no of participants (studies)	**Relative effect** (95% CI)	**Anticipated absolute effect (95% CI)** **Control group** (CT / CT + placebo)	**Test group** (Xuezhikangs + CT)	**Difference**	**Quality of the evidence (GRADE)**	**What happens**
**Left ventricular ejection fraction (LVEF), 1320 (15 studies)**	—	—	—	**5.18% more** (3.80% to 6.56% more)	⊕⊕⊖⊖ **low due to risk of bias[1] and inconsistency[4]**	May increase LVEF.
**Outcomes,** no of	**Relative effect**	**Anticipated absolute effect (95% CI)**			**Quality of the evidence**	**What happens**

participants (studies)	(95% CI)	**Control group** (CT / CT + placebo)	**Test group** (Xuezhikangs + CT)	**Difference**	**(GRADE)**	
**Left ventricular end-diastolic diameter (LVEDD), 250 (3 studies)**	—	—	—	**2.80 mm less** (1.88 to 3.71 less)	⊕⊖⊖⊖ **very low due to risk of bias[1], imprecision[2] and inconsistency[5]**	Uncertain to decrease LVEDD.

Abbreviations: CT, conventional Treatment; CI, confidence interval; OR: odds ratio; GRADE, Grading of Recommendations Assessment, Development and Evaluation.

* The basis for the **risk in the control group** (e.g., the median control group risk across studies) is provided in footnotes. The **risk in the intervention group** (and its 95% confidence interval) is based on the assumed risk in the comparison group and the **relative effect** of the intervention (and its 95% CI).

[1] High risk of bias due to unmentioned allocation concealment and insufficient blind method implementation.

[2] Imprecision due to few events.

[3] Serious unexplained inconsistency (large heterogeneity I^2 = 90%, P-value ($P < 0.0001$), point estimates, and confidence intervals vary considerably).

[4] Serious unexplained inconsistency (large heterogeneity I^2 = 85%, P-value ($P = 0.001$), point estimates, and confidence intervals vary considerably).

[5] Serious unexplained inconsistency (large heterogeneity I^2 = 85%, P-value ($P = 0.001$), point estimates, and confidence intervals vary considerably).

[56] Meta-analysis and trial sequential analysis of Yixinshu Capsules combined with western medicine for chronic heart failure (Author: Y.H. Cai, W.P. Sun, J.Y. Li, L. Zhang, J.M. Wen, and W. Wu)

Patient or population: chronic heart failure (CHF)

Settings: inpatients and outpatient

Intervention: Yixinshu Capsule (9#~12#/day) + CT

Comparison: CT

**Outcomes,** no of participants (studies)	**Relative effect** (95% CI)	**Anticipated absolute effect (95% CI)** **Control group** (CT)	**Test group** (Yixinshu Capsule + CT)	**Difference**	**Quality of the evidence (GRADE)**	**What happens**
**6-minute walk test (6-MWT), 428 (5 studies)**	—	—	—	**29.99 meters more** (22.87 to 37.11 more)	⊕⊕⊕⊖ **moderate due to risk of bias[1]**	Probably to increase 6-minute walk test distance.

**Outcomes,** no of participants (studies)	**Relative effect** (95% CI)	**Anticipated absolute effect (95% CI)** **Control group** (CT)	**Test group** (Yixinshu Capsule + CT)	**Difference**	**Quality of the evidence (GRADE)**	**What happens**
**Left ventricular ejection fraction (LVEF), 2291 (19 studies)**	—	—	—	**2.46% more** (2.25% to 2.67% more)	⊕⊕⊖⊖ **low due to risk of bias[1] and publication bias[2]**	May increase LVEF.

**Outcomes,** no of participants (studies)	**Relative effect** (95% CI)	**Anticipated absolute effect (95% CI)** **Control group** (CT)	**Test group** (Yixinshu Capsule + CT)	**Difference**	**Quality of the evidence (GRADE)**	**What happens**
**Left ventricular end-diastolic diameter (LVEDD), 1333 (10 studies)**	—	—	—	**2.04 mm less** (1.18 to 2.9 less)	⊕⊕⊖⊖ **low due to risk of bias[1] and inconsistency[3]**	May decrease LVEDD.

Abbreviations: CT, conventional treatment; CI, confidence interval; RR, risk ratio; GRADE, Grading of Recommendations Assessment, Development and Evaluation.

***** The basis for the **risk in the control group** (e.g., the median control group risk across studies) is provided in footnotes. The **risk in the intervention group** (and its 95% confidence interval) is based on the assumed risk in the comparison group and the **relative effect** of the intervention (and its 95% CI).

[1] High risk of bias due to unmentioned allocation concealment and insufficient blind method implementation.

[2] The funnel chart is asymmetric, suggesting publication bias.

[3] Serious unexplained inconsistency (large heterogeneity I^2 = 62.9%, *P*-value (*P* = 0.004), point estimates, and confidence intervals vary considerably).

Supplementary Material 5 - Grading of evidence quality based on different outcome indicators

Study ID	Participants	Treatment group	Control group	Number of RCTs (sample size)	Statistics	Effect size[95% CI]	p-value	Grading of evidence quality
**Mortality**								
Sun J 2016	HF	QQC(NA)+CT	CT	6(539)	RR	0.53[0.27, 1.07]	P>0.05	Low[a, d]
**Hospitalization rate**								
Sun J 2016	HF	QQC(NA)+CT	CT	9(669)	RR	0.49[0.38, 0.64]	P<0.05	Moderate[a]
Wang SH 2013	CHF	QYDP(0.5g,tid)+CT	CT	2(248)	RR	0.52[0.33, 0.81]	p=0.004	Moderate[a]
Liu JG 2014	CHF	QYDP(0.5g,tid)+CT	CT	3(365)	OR	0.41[0.23, 0.72]	p=0.002	Moderate[a]
**Clinical comprehensive efficacy**								
Shang YD 2013	CHF	QQC(0.9~1.2g,tid)+CT	CT	12(1110)	RR	1.24[1.17,1.31]	p<0.00001	Low[a, e]
Li XW 2014	CHF	QQC(NA)+CT	CT	16(1422)	RR	1.18[1.13, 1.24]	p<0.00001	Low[a, e]
Feng Y 2015	DHF	QQC(NA)+CT	CT	14(1220)	RR	1.29[1.21, 1.36]	p<0.00001	Low[a, e]
Sun YL 2019	HF-ICM	QQC(NA)+CT	CT or CT+Placebo	19(1611)	RR	1.21[1.16, 1.27]	p<0.00001	Moderate[a]
Wang SH 2013	CHF	QYDP(0.5g,tid)+CT	CT	7(887)	RR	1.18[1.12, 1.25]	p<0.00001	Moderate[a]
Wang Y 2015	CHF	QYDP(0.5g,tid)+CT	CT	16(1791)	OR	3.82 [2.83, 5.16]	p<0.00001	Low[a, e]
Tian Y 2016	HF-CHD	QYDP(0.5g,tid)+CT		12(1298)	RR	1.16[1.11, 1.21]	p<0.00001	Low[a, e]
An YP 2015	CHF	SBP(22.5~45mg,tid)+CT		15(1327)	OR	3.75[2.72, 5.16]	p<0.00001	Low[a, e]
Jin B 2015	CHF	SBP(NA)+CT	CT	19(1560)	RR	1.18[1.13, 1.24]	p<0.00001	Low[a, e]
Dong T 2018	CHF	SBP(22.5~67.5mg,tid)+CT	CT or CT+Placebo	17(1621)	OR	3.88[2.87, 5.26]	p<0.00001	Low[a, e]
Lin XD 2016	HF-ICM	SBP(NA)+CT+Trimetazidine	CT+Trimetazidine	12(1186)	RR	1.30[1.23, 1.38]	p<0.00001	Low[a, e]
Liang YY 2017	HF+Arrhythmia	WK(5g,tid; 9g,tid)+CT+Amiodarone	CT+Amiodarone	4(386)	OR	5.48[2.59, 11.61]	p<0.00001	Moderate[a]
Liu HT 2015	CHF	TC(2~4#,tid)+CT	CT	10(915)	OR	2.76[1.93, 3.95]	p<0.00001	Low[a, e]
He X 2019	HF-CHD	TC(2~4#,tid)+CT	CT	16(1632)	OR	4.28[3.04, 6.01]	p<0.00001	Moderate[a]
Wang Q 2016	CHF	CDDP(10#,tid)+CT	CT	9(912)	RR	1.22[1.15, 1.29]	p<0.00001	Low[a, e]
Lai RK 2018	CHF	CDDP(NA)+CT	CT	17(1440)	RR	1.21[1.16, 1.27]	p<0.00001	Low[a, e]
Wu CJ 2017	CHF	ZC(NA)+CT	CT	10(901)	OR	4.35[2.97, 6.36]	p<0.00001	Low[a, e]
Li JT 2018	CHF	BQT(4#,tid)+CT	CT	5(334)	OR	4.54[2.23, 9.26]	p<0.0001	Moderate[a]
Chen QY 2018	CHF	YT(0.9~1.2g,tid)+CT	CT	12(1232)	OR	3.24[2.33, 4.49]	p<0.00001	Moderate[a]
Zhang H 2018	CHF	CT	CT or CT+Placebo	5(382)	OR	3.04[1.81, 5.10]	p<0.00001	Moderate[a]

Study ID	Participants	Treatment group	Control group	Number of RCTs (sample size)	Statistics	Effect size[95% CI]	p-value	Grading of evidence quality
**New York Heart Association (NYHA) cardiac function efficacy**								
Liu CX 2010	CHF	QQC(NA)+CT	CT or CT+Placebo	4(253)	RR	1.21[1.08, 1.36]	P=0.0009	Moderate[a]
Liu XH 2015	CHF	QQC(NA)+CT	CT or CT+Placebo	51(4510)	RR	1.23[1.19, 1.26]	P<0.001	Low[a, e]
Jiang T 2015	CHF	QQC(1.2g,tid)+CT	CT	16(1237)	OR	3.83[2.78, 5.28]	p<0.00001	Low[a, e]
Xu Q 2015	HFpEF	QQC(NA)+CT	CT or CT+Placebo	7(586)	RR	1.41[1.15, 1.71]	p=0.0007	Moderate[a]
Sun J 2016	HF	QQC(NA)+CT	CT	54(4603)	RR	1.38[0.29, 1.48]	p > 0.05	Low[a, d]
Wang SH 2013	CHF	QYDP(0.5g,tid)+CT	CT	7(628)	RR	1.18[1.10, 1.27]	p<0.00001	Moderate[a]
Liu JG 2014	CHF	QYDP(0.5g,tid)+CT	CT	16(2007)	RD	0.14[0.11, 0.17]	p<0.00001	Low[a, e]
Gao CC 2015	CHF	QYDP(0.5g,tid)+CT	CT	13(1590)	OR	2.75[2.07, 3.66]	p<0.00001	Low[a, e]
Wang Y 2015	CHF	QYDP(0.5g,tid)+CT	CT	6(576)	OR	4.68[2.65, 8.26]	p<0.00001	Moderate[a]
Zhang YL 2019	CHF	QYDP(NA)+CT	CT	6 (634)	RR	1.25[1.16,1.35]	p<0.00001	Moderate[a]
Cao Y 2017	CHF	ZC(0.25~1g,tid; 0.5g,bid)+CT	CT	12(1033)	RR	1.27[1. 20, 1.35]	p<0.00001	Low[a, e]
Chen YQ 2016	HF	ZC(0.25~1g,tid)+CT	CT	10(831)	OR	3.71[2.531, 5.445]	p<0.00001	Low[a, e]
Li JT 2018	CHF	BQT(4#,tid)+CT	CT	2(128)	OR	4.41[1.63, 11.93]	p=0.003	Moderate[a]
Mo XY 2018	CHF	BQT(4#,tid)+CT	CT	3(188)	RR	1.25[1.09, 1.43]	p=0.001	Moderate[a]
**Minnesota Life Heart Failure Quality of Life Questionnaire (MLHFQ) score**								
Liu CX 2010	CHF	QQC(NA)+CT	CT or CT+Placebo	1(60)	WMD	-8.00[-12.52, -3.48]	NA	Low[a, d]
Shang YD 2013	CHF	QQC(0.9~1.2g,tid)+CT	CT	2(111)	WMD	-4.20[-8.05, -0.36]	P= 0.03	Low[a, d]
Li XW 2014	CHF	QQC(NA)+CT	CT	2(134)	WMD	-7.69[-10.99, -4.39]	p<0.00001	Low[a, d]
Liu XH 2015	CHF	QQC(NA)+CT	CT or CT+Placebo	8(562)	WMD	-6.42[-7.58,-5.26]	p<0.001	Low[a, e]
Li ZY 2018	CHF	QQC(1.2g,tid)+CT	CT or CT+Placebo	2(200)	WMD	-8.11[-10.23,-6.0]	p<0.00001	Very low[a, b, d]
Li JT 2018	CHF	BQT(4#,tid)+CT	CT	3(188)	WMD	-11.23[-14.45, -8.01]	p<0.00001	Low[a, d]
Mo XY 2018	CHF	BQT(4#,tid)+CT	CT	4(338)	WMD	-10.90[-13.12, -8.68]	p<0.00001	Low[a, d]
**6-minute walk test (6-MWT)**								
Liu CX 2010	CHF	QQC(NA)+CT	CT or CT+Placebo	4(233)	WMD	37.39[22.58, 52.20]	p<0.00001	Low[a, d]
Shang YD 2013	CHF	QQC(0.9~1.2g,tid)+CT	CT	6(584)	WMD	39.39[30.61, 48.16]	p<0.00001	Moderate[a]
Li XW 2014	CHF	QQC(NA)+CT	CT	6(542)	WMD	41.44[33.30, 49.59]	p<0.00001	Moderate[a]
Liu XH 2015	CHF	QQC(NA)+CT	CT or CT+Placebo	38(3654)	WMD	48.52[40.15,56.88]	p<0.001	Moderate[a]
Li ZY 2018	CHF	QQC(1.2g,tid)+CT	CT or CT+Placebo	8(690)	WMD	53.81[46.9, 60.73]	p<0.00001	Moderate[a]
Jiang T 2015	CHF	QQC(1.2g,tid)+CT	CT	6(994)	WMD	34.10[25.88, 42.32]	p<0.00001	Moderate[a]

Study ID	Participants	Treatment group	Control group	Number of RCTs (sample size)	Statistics	Effect size[95% CI]	p-value	Grading of evidence quality
Xu Q 2015	HFpEF	QQC(NA)+CT	CT or CT+Placebo	3(232)	WMD	67.4[2.17,132.63]	P=0.04	Very low[a, b, d]
Sun YL 2019	HF-ICM	QQC(NA)+CT	CT or CT+Placebo	8 (776)	WMD	33.20[24.70, 41.70]	p<0.00001	Low[a, b]
Sun J 2016 [subgroup analysis according to interventions]								
QQC+CT vs. CT	HF	QQC(NA)+CT	CT	42(-)	WMD	47.21[44.53, 49.90]	NA	Low[a, b]
QQC+CT vs. CT+Placebo	HF	QQC(NA)+CT	CT+Placebo	3(681)	WMD	49.55[38.79, 60.32]	NA	Moderate[a]
Wang SH 2013	CHF	QYDP(0.5g,tid)+CT	CT	7(882)	WMD	94.39[71.89, 116.89]	p<0.00001	Low[a, b]
Wang Y 2015	CHF	QYDP(0.5g,tid)+CT	CT	6(584)	WMD	73.59[67.09, 80.09]	p<0.00001	Moderate[a]
Zhang YL 2019	CHF	QYDP(NA)+CT	CT	4(336)	WMD	50.13[22.32, 77.93]	p=0.0004	Very low[a, b, d]
Qu F 2014	HF-ICM	QYDP(0.5g,tid)+CT	CT	2(128)	WMD	48.31[39.51, 57.10]	p<0.00001	Low[a, d]
Tian Y 2016	HF-CHD	QYDP(0.5g,tid)+CT	CT	11(1162)	WMD	71.37[53.28,89.47]	p<0.00001	Very low[a, b, e]
An YP 2015	CHF	SBP(22.5~45mg,tid)+CT	CT or CT+Placebo	12(1089)	WMD	38.05[26.94,49.15]	p<0.00001	Low[a, b]
Jin B 2015	CHF	SBP(NA)+CT	CT	16(1413)	WMD	41.88[33.39, 50.37]	p<0.00001	Very low[a, b, e]
Dong T 2018	CHF	SBP(22.5~67.5mg,tid)+CT	CT or CT+Placebo	15(1439)	WMD	40.15[30.40, 49.91]	p<0.00001	Low[a, b]
Lin XD 2016	HF-ICM	SBP(NA)+CT+Trimetazidine	CT+Trimetazidine	2(242)	WMD	56.98[42.76, 71.19]	p<0.00001	Low[a, d]
Lai RK 2018	CHF	CDDP(NA)+CT	CT	4(347)	WMD	44.89[32.59, 57.18]	p<0.00001	Low[a, d]
Chen QY 2018	CHF	YT(0.9~1.2g,tid)+CT	CT	4(424)	WMD	47.21[37.45, 56.97]	p<0.00001	Moderate[a]
Cai YH 2018	CHF	YC(9~12# or day)+CT	CT	5(428)	WMD	29.99[22.87, 37.11]	p<0.001	Moderate[a]
**Brain natriuretic peptide (BNP)**								
Liu CX 2010	CHF	QQC(NA)+CT	CT or CT+Placebo	1(60)	WMD	-118.00[-231.56,-4.44]	NA	Low[a, d]
Shang YD 2013	CHF	QQC(0.9~1.2g,tid)+CT	CT	4(401)	WMD	-113.78[-194.75,-32.82]	p=0.006	Low[a, b]
Li XW 2014	CHF	QQC(NA)+CT	CT	5(444)	WMD	-95.86[-147.77,-43.96]	p=0.0003	Low[a, b]
Zhuang X 2015	CHF	QQC(0.9~1.2g,tid)+CT	CT	9(842)	SMD	-0.77[-0.91, -0.63]	p<0.00001	Low[a, b]
Liu XH 2015	CHF	QQC(NA)+CT	CT or CT+Placebo	13(959)	WMD	-86.64[-120.13, -53.15]	p<0.001	Very low[a, b, e]
Li ZY 2018	CHF	QQC(1.2g,tid)+CT	CT or CT+Placebo	10(1020)	WMD	-194.97[-287.95, -101.99]	p<0.00001	Low[a, b]
Jiang T 2015	CHF	QQC(1.2g,tid)+CT	CT	8(791)	WMD	-71.69[-98.21, -45.16]	p<0.00001	Low[a, b]
Xu Q 2015	HFpEF	QQC(NA)+CT	CT or CT+Placebo	5(414)	WMD	-93.46[-121.98, -64.93]	p<0.00001	Low[a, b]
Wang SH 2013	CHF	QYDP(0.5g,tid)+CT	CT	5(478)	WMD	-194.85[-442.61, 52.91]	P=0.12	Very low[a, b, d]
Wang Y 2015	CHF	QYDP(0.5g,tid)+CT	CT	4(338)	WMD	-48.92[-57.49, -40.35]	p<0.00001	Low[a, d]
Zhang YL 2019	CHF	QYDP(NA)+CT	CT	4(370)	WMD	-29.47[-37.29, -21.64]	p<0.00001	Low[a, d]
Qu F 2014	HF-ICM	QYDP(0.5g,tid)+CT	CT	3(296)	WMD	-55.76[-66.53, -44.99]	p<0.00001	Very low[a, b, d]

Study ID	Participants	Treatment group	Control group	Number of RCTs (sample size)	Statistics	Effect size[95% CI]	p-value	Grading of evidence quality
Tian Y 2016	HF-CHD	QYDP(0.5g,tid)+CT	CT	4(390)	WMD	-63.55[-85.48, -41.63]	p<0.00001	Very low[a, b, d]
An YP 2015	CHF	SBP(22.5~45mg,tid)+CT	CT or CT+Placebo	5(437)	WMD	-78.64[-239.02, 81.73]	P=0.34	Very low[a, b, d]
Jin B 2015	CHF	SBP(NA)+CT	CT	8(748)	WMD	-142.64 [-228.88, -56.40]	P=0.001	Low[a, b]
Dong T 2018	CHF	SBP(22.5~67.5mg,tid)+CT	CT or CT+Placebo	4(388)	WMD	-66.95[-108.57, -25.34]	p=0.002	Very low[a, b, d]
Chen Y 2014	HF	WK(6g,tid; 9g,tid)+CT	CT	7(522)	SMD	-4.18[-5.89, -2.47]	p<0.00001	Low[a, b]
Lai RK 2018	CHF	CDDP(NA)+CT	CT	4(369)	WMD	-197.84[-241.35, -154.33]	p<0.00001	Very low[a, b, d]
Cao Y 2017	CHF	ZC(0.25~1g,tid; 0.5g,bid)+CT	CT	5(441)	WMD	-601.02[-931.05, -270. 99]	p<0.00001	Low[a, b]
Chen QY 2018	CHF	YT(0.9~1.2g,tid)+CT	CT	2(197)	SMD	-0.21[-0.84, 0.42]	p=0.051	Very low[a, b, d]
Zhang H 2018	CHF	XZK(0.3g,bid; 0.6g,qd; 0.6g,bid; 0.6g,tid)+CT	CT or CT+Placebo	3(252)	WMD	-252.25[-318.80, -185.70]	p<0.00001	Very low[a, b, d]
**N-terminal pro-brain natriuretic peptide (NT-proBNP)**								
Shang YD 2013	CHF	QQC(0.9~1.2g,tid)+CT	CT	3(242)	WMD	-90.21[-120.35, -60.07]	p<0.00001	Low[a, d]
Zhuang X 2015	CHF	QQC(0.9~1.2g,tid)+CT	CT	5(430)	SMD	-0.38[-0.57, -0.19]	P=0.0001	Low[a, b]
Liu XH 2015	CHF	QQC(NA)+CT	CT or CT+Placebo	8(1666)	WMD	-236.46[-308.12, -164.81]	p<0.001	Low[a, b]
Xu Q 2015	HFpEF	QQC(NA)+CT	CT or CT+Placebo	3(222)	WMD	-76.62[-118.32,-34.92]	P=0.005	Low[a, d]
Feng Y 2015 [subgroup analysis according to treatment courses]								
= 1 month	DHF	QQC(NA)+CT	CT	11(889)	WMD	-247.18[-336.12,-158.25]	p<0.00001	Very low[a, b, e]
= 6 months	DHF	QQC(NA)+CT	CT	2(179)	WMD	-384.00[-416.97 ,-351.03]	p<0.00001	Low[a, d]
Sun J 2016	HF	QQC(NA)+CT	CT	24(-)	WMD	-214.43[-269.42,-159.45]	NA	Low[a, b]
Wu CJ 2017	CHF	ZC(NA)+CT	CT	3(270)	WMD	-572.24[-717.32, -427.16]	p<0.00001	Low[a, d]
Li JT 2018	CHF	BQT(4#,tid)+CT	CT	3(214)	SMD	-1.58[-1.88, -1.282]	p<0.01	Very low[a, b, d]
Mo XY 2018	CHF	BQT(4#,tid)+CT	CT	4(393)	SMD	-2.02[-3.02, -1.02]	p<0.0001	Very low[a, b, d]
Chen QY 2018	CHF	YT(0.9~1.2g,tid)+CT	CT	2(176)	SMD	-1.57[-2.50, -0.63]	p=0.001	Very low[a, b, d]
**Left ventricular ejection fraction (LVEF)**								
Liu CX 2010	CHF	QQC(NA)+CT	CT or CT+Placebo	4(260)	WMD	3.97[2.09， 5.85]	p<0.0001	Low[a, d]
Shang YD 2013	CHF	QQC(0.9~1.2g,tid)+CT	CT	10(981)	WMD	6.04[4.57, 7.52]	p<0.00001	Low[a, b]
Li XW 2014	CHF	QQC(NA)+CT	CT	13(1204)	WMD	4.89[3.03, 6.75]	p<0.00001	Low[a, b]
Zhuang X 2015	CHF	QQC(0.9~1.2g,tid)+CT	CT	29(2570)	SMD	0.48[0.40, 0.56]	p<0.00001	Very low[a, b, e]
Liu XH 2015	CHF	QQC(NA)+CT	CT or CT+Placebo	57(5361)	WMD	5.16[4.24,6.08]	p<0.001	Low[a, b]
Li ZY 2018	CHF	QQC(1.2g,tid)+CT	CT or CT+Placebo	17(1580)	WMD	5.24[3.38, 7.11]	p<0.00001	Very low[a, b, e]

Study ID	Participants	Treatment group	Control group	Number of RCTs (sample size)	Statistics	Effect size[95% CI]	p-value	Grading of evidence quality
Jiang T 2015	CHF	QQC(1.2g,tid)+CT	CT	16(1884)	WMD	6.32[3.92, 8.73]	p<0.00001	Very low[a, b, e]
Sun YL 2019	HF-ICM	QQC(NA)+CT	CT or CT+Placebo	16(1563)	WMD	7.28[5.18, 9.38]	p<0.00001	Low[a, b]
Sun J 2016	HF	QQC(NA)+CT	CT	84(-)	WMD	5.87[5.28,6.47]	NA	Low[a, b]
Wang SH 2013 [subgroup analysis according to cause]								
coronary artery disease	CHF	QYDP(0.5g,tid)+CT	CT	3(403)	WMD	8.34[6.23, 10.45]	p<0.00001	Moderate[a]
other diseases	CHF	QYDP(0.5g,tid)+CT	CT	12(1272)	WMD	5.57[4.16, 6.97]	p<0.00001	Very low[a, b, e]
Wang Y 2015	CHF	QYDP(0.5g,tid)+CT	CT	18(2038)	WMD	7.61[6.24, 8.98]	p<0.00001	Low[a, e]
Zhang YL 2019	CHF	QYDP(NA)+CT	CT	7(607)	WMD	4.64[3,82, 5.47]	p<0.00001	Moderate[a]
Chang MZ 2019	CHF	QYDP(0.5g,tid)+CT	CT	12(877)	WMD	6.05[0.94, 11.16]	p=0.02	Low[a, b]
Qu F 2014	HF-ICM	QYDP(0.5g,tid)+CT	CT	6(673)	WMD	7.00[5.00, 8.00]	p=0.0003	Moderate[a]
Tian Y 2016	HF-CHD	QYDP(0.5g,tid)+CT	CT	12(1275)	WMD	6.55[5.35, 7.74]	p<0.00001	Very low[a, b, e]
An YP 2015	CHF	SBP(22.5~45mg,tid)+CT	CT or CT+Placebo	15(1320)	WMD	5.18[3.8, 6.56]	p<0.00001	Low[a, b]
Jin B 2015	CHF	SBP(NA)+CT	CT	31(2596)	WMD	4.76[3.64, 5.87]	p<0.00001	Very low[a, b, e]
Dong T 2018	CHF	SBP(22.5~67.5mg,tid)+CT	CT or CT+Placebo	21(1913)	WMD	3.89[2.70, 5.07]	p<0.00001	Low[a, b]
Lin XD 2016	HF-ICM	SBP(NA)+CT+Trimetazidine	CT+Trimetazidine	7(716)	SMD	1.55[0.85, 2.25]	p<0.0001	Low[a, b]
Chen Y 2014	HF	WK(6g,tid; 9g,tid)+CT	CT	11(903)	WMD	3.52[2.40, 4.64]	p<0.00001	Low[a, b]
Liang YY 2017	HF+Arrhythmia	WK(5g,tid; 9g,tid)+CT+Amiodarone	CT+Amiodarone	4(386)	WMD	7.56[6.65, 8.46]	p<0.00001	Low[a, d]
Liu HT 2015	CHF	TC(2~4#,tid)+CT	CT	5(403)	WMD	5.28[2.57, 7.99]	p=0.0001	Low[a, b]
He X 2019	HF-CHD	TC(2~4#,tid)+CT	CT	13(1480)	WMD	6. 64[5. 97, 7. 31]	p<0.00001	Low[a, b]
Lai RK 2018	CHF	CDDP(NA)+CT	CT	10(876)	WMD	4.67[3.31, 6.02]	p<0.00001	Low[a, b]
Wu CJ 2017	CHF	ZC(NA)+CT	CT	9(851)	WMD	7.89[4.24, 11.54]	p<0.0001	Low[a, b]
Cao Y 2017	CHF	ZC(0.25~1g,tid; 0.5g,bid)+CT	CT	11(987)	WMD	6.78[3. 68, 9. 88]	p<0.00001	Very low[a, b, e]
Chen YQ 2017 [subgroup analysis according to treatment courses]								
< 6 weeks	HF	ZC(0.25~1g,tid)+CT	CT	4(368)	SMD	1.38[1.14, 1.62]	p<0.0001	Very low[a, b, d]
≥ 6 weeks	HF	ZC(0.25~1g,tid)+CT	CT	6(469)	SMD	0.85[0.66, 1.04]	p<0.0001	Moderate[a]
Li JT 2018	CHF	BQT(4#,tid)+CT	CT	6(433)	WMD	3.88[3.18, 4.59]	p<0.00001	Moderate[a]
Mo XY 2018	CHF	BQT(4#,tid)+CT	CT	7(573)	WMD	4.31[3.73, 4.90]	p<0.00001	Moderate[a]
Chen QY 2018	CHF	YT(0.9~1.2g,tid)+CT	CT	8(818)	WMD	2.92[-1.74, 7.59]	P=0.22	Very low[a, b, d]
Zhang H 2018	CHF	XZK(0.3g,bid; 0.6g,qd; 0.6g,bid; 0.6g,tid)+CT	CT or CT+Placebo	5(368)	WMD	6.96[5.91, 8.02]	p<0.00001	Low[a, d]

Study ID	Participants	Treatment group	Control group	Number of RCTs (sample size)	Statistics	Effect size[95% CI]	p-value	Grading of evidence quality
Cai YH 2018	CHF	YC(9~12# or day)+CT	CT	19(2291)	WMD	2.46[2.25, 2.67]	p<0.001	Low[a, e]
**Left ventricular end-diastolic diameter (LVEDD)**								
Liu CX 2010	CHF	QQC(NA)+CT	CT or CT+Placebo	4(221)	WMD	-2.81[-4.22, -1.41]	p<0.0001	Low[a, d]
Shang YD 2013	CHF	QQC(0.9~1.2g,tid)+CT	CT	3(340)	WMD	-4.28[-6.41, -2.14]	p<0.0001	Very low[a, b, d]
Li XW 2014	CHF	QQC(NA)+CT	CT	6(625)	WMD	-3.22[-4.80, -1.64]	p<0.0001	Low[a, b]
Zhuang X 2015	CHF	QQC(0.9~1.2g,tid)+CT	CT	13(1192)	SMD	-0.40[-0.51, -0.28]	p<0.00001	Very low[a, b, e]
Li ZY 2018	CHF	QQC(1.2g,tid)+CT	CT or CT+Placebo	10(921)	WMD	-0.94[-1.46, -0.43]	p =0.00031	Low[a, b]
Sun YL 2019	HF-ICM	QQC(NA)+CT	CT or CT+Placebo	10(947)	WMD	-4.61[- 5.38, -3.84]	p<0.00001	Moderate[a]
Liu JG 2014	CHF	QYDP(0.5g,tid)+CT	CT	5(510)	SMD	-0.54[-0.76, -0.31]	p<0.00001	Low[a, b]
Wang Y 2015	CHF	QYDP(0.5g,tid)+CT	CT	4(411)	WMD	-1.19[-2.01, -0.37]	P=0.005	Moderate[a]
Shan QY 2017	CHF	QYDP(NA)+CT	CT	8(786)	WMD	-2.55[-3.63, -1.47]	p<0.00001	Low[a, b]
An YP 2015	CHF	SBP(22.5~45mg,tid)+CT	CT or CT+Placebo	8(680)	WMD	-2.88[-3.45, -2.3]	p<0.00001	Moderate[a]
Jin B 2015	CHF	SBP(NA)+CT	CT	15(1186)	WMD	-2.90[-4.42, -1.39]	P=0.0002	Very low[a, b, e]
Lin XD 2016	HF-ICM	SBP(NA)+CT+Trimetazidine	CT+Trimetazidine	5(524)	SMD	-1.57[-1.91, -1.22]	p<0.00001	Low[a, b]
Liu HT 2015	CHF	TC(2~4#,tid)+CT	CT	3(228)	WMD	-2.19[-3.52, -0.86]	p =0.001	Low[a, d]
Lai RK 2018	CHF	CDDP(NA)+CT	CT	5(440)	WMD	-2.99[-4.22, -1.75]	p<0.00001	Moderate[a]
Zhang H 2018	CHF	XZK(0.3g,bid; 0.6g,qd; 0.6g,bid; 0.6g,tid)+CT	CT or CT+Placebo	3(250)	WMD	-2.80[-3.71, -1.88]	p<0.00001	Very low[a, b, d]
Cai YH 2018	CHF	YC(9~12# or day)+CT	CT	10(1333)	WMD	-2.04[-2.9, -1.18]	p<0.001	Low[a, b]
**Left ventricular end-systolic diameter (LVESD)**								
Zhuang X 2015	CHF	QQC(0.9~1.2g,tid)+CT	CT	3(276)	SMD	-0.90[-1.15, -0.65]	p<0.00001	Very low[a, b, d]
Liu JG 2014	CHF	QYDP(0.5g,tid)+CT	CT	5(450)	SMD	-0.53[-0.72, -0.34]	p<0.00001	Low[a, b]
Wang Y 2015	CHF	QYDP(0.5g,tid)+CT	CT	5(551)	WMD	-1.48[-2.12, -0.83]	p<0.00001	Moderate[a]
Shan QY 2017	CHF	QYDP(NA)+CT	CT	8(786)	WMD	-1.82[-2.34, -1.30]	p<0.00001	Moderate[a]
Jin B 2015	CHF	SBP(NA)+CT	CT	7(491)	WMD	-2.22[-3.19, -1.25]	P<0.01	Moderate[a]
Lai RK 2018	CHF	CDDP(NA)+CT	CT	2(184)	WMD	-3.47[-5.22, -1.72]	p=0.0001	Low[a, d]
**Cardiac output (CO)**								
Shang YD 2013	CHF	QQC(0.9~1.2g,tid)+CT	CT	3(240)	WMD	0.54[0.05, 1.02]	P=0.03	Very low[a, b, d]
Li XW 2014	CHF	QQC(NA)+CT	CT	5(460)	WMD	0.22[0.00, 0.44]	P=0.05	Very low[a, b, d]
Jiang T 2015	CHF	QQC(1.2g,tid)+CT	CT	5(499)	WMD	0.48[0.03,0.93]	P=0.04	Low[a, b]

Study ID	Participants	Treatment group	Control group	Number of RCTs (sample size)	Statistics	Effect size[95% CI]	p-value	Grading of evidence quality
Liu JG 2014	CHF	QYDP(0.5g,tid)+CT	CT	3(430)	SMD	1.43[1.22, 1.64]	p<0.00001	Low[a, b]
Dong T 2018	CHF	SBP(22.5~67.5mg,tid)+CT	CT or CT+Placebo	7(704)	WMD	0.84[0.68, 0.99]	p<0.00001	Low[a, b]
He X 2019	HF-CHD	TC(2~4#,tid)+CT	CT	7(800)	WMD	0.40[0.32, 047]	p<0.00001	Moderate[a]
Wu CJ 2017	CHF	ZC(NA)+CT	CT	4(434)	WMD	0.79[0.61, 0.98]	p<0.00001	Low[a, b]
Cao Y 2017	CHF	ZC(0.25~1g,tid; 0.5g,bid)+CT	CT	4(434)	WMD	0.80[0.55, 1.06]	p<0.00001	Low[a, b]
**Stroke volume (SV)**								
Dong T 2018	CHF	SBP(22.5~67.5mg,tid)+CT	CT or CT+Placebo	7(535)	WMD	7.43[4.42, 10.44]	p<0.00001	Low[a, b]
Lai RK 2018	CHF	CDDP(NA)+CT	CT	3(170)	WMD	4.40[1.95, 6.84]	p=0.0004	Very low[a, b, d]
Cao Y 2017	CHF	ZC(0.25~1g,tid; 0.5g,bid)+CT	CT	3(345)	WMD	7.62[6.39, 8.84]	p<0.00001	Low[a, d]
**TCM symptom efficacy**								
Feng Y 2015	DHF	QQC(NA)+CT	CT	6(442)	RR	1.24[1.14, 1.35]	p<0.00001	Moderate[a]
Zhang YL 2019	CHF	QYDP(NA)+CT	CT	5(338)	RR	1.23[1.07, 1.43]	P=0.005	Low[a, d]
Cao Y 2017	CHF	ZC(0.25~1g,tid; 0.5g,bid)+CT	CT	3(227)	RR	1.46[1.25, 1.72]	p<0.00001	Low[a, d]
**Lee's Heart Failure Score**								
Mo XY 2018	CHF	BQT(4#,tid)+CT	CT	3(304)	WMD	-1.09[-1.36,-0.83]	p<0.00001	Low[a, d]
**hs-CRP**								
Chen QY 2018	CHF	YT(0.9~1.2g,tid)+CT	CT	2(154)	SMD	-0.39[-0.80,0.02]	P=0.06	Very low[a, b, d]
**the ratio of peak mitral valve blood flow velocity in early left ventricular diastole to peak mitral valve blood flow velocity in atrial systole (E/A)**								
Xu Q 2015	HFpEF	QQC(NA)+CT	CT or CT+Placebo	5(406)	WMD	0.14[0.07,0.21]	p=0.0001	Low[a, b]
Feng Y 2015	DHF	QQC(NA)+CT	CT	8(614)	WMD	0.16[0.10,0.23]	p<0.00001	Low[a, b]
**the ratio of peak mitral valve blood flow velocity in early diastole to peak mitral valve annulus velocity in early diastole (E/E')**								
Feng Y 2015	DHF	QQC(NA)+CT	CT	7(508)	WMD	-1.97[-2.65,-1.30]	p<0.00001	Moderate[a]
**Left ventricular end-diastolic volume (LVEDV)**								
Sun YL 2019	HF-ICM	QQC(NA)+CT	CT or CT+Placebo	4(387)	WMD	-34.43[- 38.81, -30.05]	p<0.00001	Low[a, d]
**Left ventricular end-systolic volume (LVESV)**								
Sun YL 2019	HF-ICM	QQC(NA)+CT	CT or CT+Placebo	4(387)	WMD	-9.06[- 13.16, -6.05]	p<0.00001	Low[a, d]
**E peak deceleration time (DT)**								
Liu HT 2015	CHF	TC(2~4#,tid)+CT	CT	2(160)	WMD	-22.65[-45.17, -0.12]	p=0.05	Very low[a, b, d]
**Interventricular septal thickness at diastole (IVSd)**								
Lai RK 2018	CHF	CDDP(NA)+CT	CT	2(184)	WMD	-0.66[-0.97,-0.35]	p<0.0001	Low[a, d]

Study ID	Participants	Treatment group	Control group	Number of RCTs (sample size)	Statistics	Effect size[95% CI]	p-value	Grading of evidence quality
**Left ventricular posterior wall thickness at diastole (LVPWd)**								
Lai RK 2018	CHF	CDDP(NA)+CT	CT	2(184)	WMD	-0.68[-1.00, -0.35]	p<0.0001	Low[a, d]
**Adverse events**								
Li ZY 2018	CHF	QQC(1.2g,tid)+CT	CT or CT+Placebo	6(507)	OR	0.44[0.25, 0.79]	p=0.006	Low[a, d]
Feng Y 2015	DHF	QQC(NA)+CT	CT	6(503)	RD	-0.02[-0.05, 0.01]	P=0.14	Very low[a, D]
Sun J 2016	HF	QQC(NA)+CT	CT	56(4846)	RR	0.56[0.40, 0.78]	p<0.05	Moderate[a]
He X 2019	HF-CHD	TC(2~4#,tid)+CT	CT	6(718)	RD	0.01[- 0.02, 0.04]	p=0.72	Very low[a, D]
Lai RK 2018	CHF	CDDP(NA)+CT	CT	2(184)	RR	1.25[0.57, 2.75]	p=0.58	Very low[a, D]

HF: heart failure; CHF: chronic heart failure; HFpEF: heart failure with preserved ejection fraction; DHF: diastolic heart failure; HF-ICM: heart failure caused by ischemic cardiomyopathy; HF-CHD: heart failure caused by coronary heart disease; NA: Not available; QQC: Qili Qiangxin Capsules; QYDP: Qishen Yiqi Dripping Pills; SBP: Shexiang Baoxin Pills; WK: Wenxin Keli; TC: Tongxinluo Capsules; FDDP: Fufang Danshen Dripping Pills; ZC: Zhenyuan Capsules; BQT: Buyi Qiangxin Tablets; YT: Yangxinshi Tablets; XZK: Xuezhikang; YC: Yixinshu Capsules; CT: conventional therapy; Cochrane: Cochrane Reviews' Handbook; Jadad: Jadad Rating Scale. RR: relative risk; OR: odds ratio; RD: risk difference; WMD: weighted mean difference; SMD: standardized mean difference.

[a] The limitation is a factor of downgrading.

[b] The inconsistency is a factor of downgrading.

[c] The indirectness is a factor of downgrading.

[d] The inaccuracy is a factor of downgrading.

[e] The publication bias is a factor of downgrading.

Supplementary Material 6 - List of excluded literature

Citation	Reason for exclusion
1. J. Chen, G. Wu, S. Li, J. Qu, Y. Yao, and L. Wang, "Shengmai (traditional Chinese herbal medicine) for heart failure," *Cochrane Database of Systematic Reviews*, 2004.	Unable to get complete and correct data (updated)
2. J. Chen, G. Wu, S. Li et al., "Shengmai (a traditional Chinese herbal medicine) for heart failure," *Cochrane Database of Systematic Reviews*, 2007.	Unable to get complete and correct data (updated)
3. H. Zheng, Y. Chen, J. Chen, J. Kwong, and W. Xiong, "Shengmai (a traditional Chinese herbal medicine) for heart failure," *Cochrane Database of Systematic Reviews*, 2011.	Unable to get complete and correct data (updated)
4. J. Chen, Y. Yao, H. Chen, J. Kwong, and J. Chen, "Shengmai (a traditional Chinese herbal medicine) for heart failure," *Cochrane Database of Systematic Reviews*, 2012.	Unable to get complete and correct data (updated)
5. Q. Zhou, W. Qin, S. B. Liu, J. Kwong, J. Zhou, and J. Chen, "Shengmai (a traditional Chinese herbal medicine) for heart failure," *Cochrane Database of Systematic Reviews*, 2014.	Unable to get complete and correct data (updated)
6. J. Chen, Y. Yao, H. Chen, J. Kwong, and J. Chen, "Shengmai (a traditional Chinese herbal medicine) for heart failure," *Cochrane Database of Systematic Reviews*, 2016.	Unable to get complete and correct data (withdrawal from publication)
7. Z. Li, Y. Zhang and T. Yuan, "Clinical efficacy and safety of Nuanxin Capsule for chronic heart failure: a systematic review and meta-analysis," *Medicine (Baltimore)*, vol. 97, no. 27, pp.e11339, 2018.	Protocol
8. S. H. Wang, J. Y. Mao, Y. Z. Hou, J. Y. Wang, X. L. Wang, and Z. J. Li, "Routine western medicine treatment plus Qishen Yiqi Dripping Pill for treating patients with chronic heart failure: a systematic review of randomized control trials," *Chinese Journal of Integrated Traditional and Western Medicine*, vol. 33, no. 11, pp.1468-1475, 2013.	Repeated research

9. Z.Q. Liao, "Meta-analysis of the influence of Qili Qiangxin Capsule on left ventricular ejection fraction and end-diastolic diameter of patients with chronic heart failure," Suzhou University, 2014.	Repeated research
10. D.Z. Kong, "Qili Qiangxin Capsule combined with conventional therapy in treating chronic heart failure: a meta-analysis and systematic review," *Integrative Medicine Research*, vol. 4, no. 01, pp.104-105, 2015.	Repeated research
11. J. Sun, K. Zhang, W. J. Xiong et al., "A systematic review and meta-analysis of a Chinese herbal remedy, Qili Qiangxin, as an adjuvant therapy to patients with heart failure," *Integrative Medicine Research*, vol. 4, no. 1, pp.127, 2015.	Repeated research
12. Y. Cao, W. Q. Wang, L. Lu, and X. M. Guo, "Adjuvant effects of Zhenyuan capsule on chronic heart failure: Meta-analysis," *China Journal of Chinese Materia Medica*, vol. 42, no. 13, pp.2583-2590, 2017.	Repeated research
13. Y.T. Wu, J. Guo, H.F. Qin, C.L, Zhu, X.C. Liu, and M.J. Zhu, "Meta-analysis of randomized controlled literatures of Qili Qiangxin capsules in the treatment of heart failure," The eighth Seminar on Evidence-Based Medicine Methods of Traditional Chinese Medicine/Integrated Traditional Chinese and Western Medicine, Henan China, 2014.	Data sources include non-RCTs
14. N. Su, T. Xu, Z. Zhou, and X. Tang, "Evaluation of efficacy and safety of Wenxin Granule in treating congestive heart failure," *China Pharmacy*, vol. 21, no. 07, pp.637-640, 2010.	Data sources include non-RCTs
15. N. Xu, H.Q. Tang, and Y.W. Zhang, "Efficacy analysis of Qili Qiangxin Capsule in treating chronic heart failure," *World Chinese Medicine*, vol. 9, no. 2, pp.237-241, 2014.	Data sources include non-RCTs
16. H.Q. Tang, Y.W. Zhang, "Meta-analysis of Qili Qiangxin Capsule for chronic heart failure," The tenth International Congress of Collateral Disease, Beijing China, 2014.	Data sources include non-RCTs
17. S. Shi, Y.T. Geng, Y.H. Hu, Q.Q. Song, B. Du, and R. Feng, "Systematic review of Wenxin Granule combined with bisoprolol in the treatment of heart failure with arrhythmia," *Chinese Journal of Experimental Traditional Medical Formulae*, vol. 22, no. 15, pp.216-219, 2016.	Data sources include non-RCTs
18. R. Zheng, G. Tian, Q. Zhang, L. Wu, Y. Xing, and H. Shang, "Clinical safety and efficacy of Wenxin	Data sources include non-RCTs

Keli-amiodarone combination on heart failure complicated by ventricular arrhythmia: a systematic review and meta-analysis," *Frontiers in Physiology*, vol. 09, pp.487, 2018.	
19. H.B. Li, Z. Li, Y. Hu, Z.H. Yang, Y.H. Li, and J.F. Xie, "Systematic review of Dengzhan Shengmai Capsule in treating coronary heart disease and chronic heart failure," *Chinese Journal Integrative Medicine on Cardio-/Cerebrovascular Disease*, vol. 17, no. 07, pp.967-971, 2019.	Non-compliance with the study population
20. H.Z. He, S.F. Liu, and W.K. Wu, "A systematic review of the comparison of the efficacy of Qili Qiangxin Capsule versus western medicine in the treatment of chronic heart failure," *Hebei Journal of Traditional Chinese Medicine*, vol. 35, no. 09, pp.1299-1301, 2013.	Non-compliance with interventions
21. M. Li, R. Qiu, G. Tian et al., "Wenxin Keli for ventricular premature complexes with heart failure: a systematic review and meta-analysis of randomized clinical trials," *Complementary Therapies in Medicine*, vol. 33, pp.85-93, 2017.	Non-compliance with interventions
22. J.P. Du, M. Guo, D.Z. Shi, and T.G. Qiu, "Efficacy of Yangxinshi Tablets in the treatment of heart failure: a Meta-analysis," *Chinese Journal Integrative Medicine on Cardio-/Cerebrovascular Disease*, vol. 16, no. 01, pp.28-31, 2018.	Non-compliance with interventions
23. Y.X. Zheng, Y.P. Li, X.L. Wang, and J.J. Gao, "Efficacy of Yangxinshi Tablets in the treatment of heart failure: a meta-analysis," *World Chinese Medicine*, vol. 13, no. 08, pp.2063-2067, 2018.	Non-compliance with interventions
24. S. Zhang and S. Hongcai, "Clinical evidence of chinese medicine prevention and treatment to chronic heart failure," *Heart*, vol. 99, pp.A214-215, 2013.	Non-compliance with interventions
25. H. Bai, Y. Li, K. Han, M. Gong, and A. Ma, "Effectiveness of Chinese herbal medicine as an adjunctive treatment for dilated cardiomyopathy in patients with heart failure," *Journal of Alternative and Complementary Medicine*, vol. 19, no. 10, pp.811-819, 2013.	Non-compliance with interventions
26. H. Shen, Q. H. Ai, Y. M. Xie, Y. Hao, J. Hu, and Y. L. Zhang, "Parenterally administered shenqi fuzheng for heart failure: a systematic review and meta-analysis," *China Journal of Chinese Materia Medica*, vol. 38, no. 18, pp.3200-3208, 2013.	Non-compliance with interventions

27. Y. L. Li, J. Q. Ju, C. H. Yang, H. Q. Jiang, J. W. Xu, and S. J. Zhang, "Oral Chinese herbal medicine for improvement of quality of life in patients with chronic heart failure: a systematic review and meta-analysis," *Quality of Life Reaearch*, vol. 23, no. 04, pp.1177-1192, 2014.	Non-compliance with interventions
28. Z.C. Zeng, W. Wu, F.X. Lin et al., "Meta-analysis of berberine in the treatment of chronic heart failure," *Jilin Journal of Traditional Chinese Medicine*, vol. 38, no. 07, pp.836-839, 2018.	Non-compliance with interventions
29. J. Liu, J.J. Lu, K. Zhou et al., "Comparison of the efficacy and acceptability of Chinese herbal medicine in adult patients with heart failure and reduced ejection fraction: study protocol for a systematic review and network meta-analysis," *BMJ Open*, vol. 8, no. 06, pp.e15678, 2018.	Non-compliance with interventions
30. J. Wang, R. Yang, F. Zhang et al., "The Effect of Chinese herbal medicine on quality of life and exercise tolerance in heart failure with preserved ejection fraction: a systematic review and meta-analysis of randomized controlled trials," *Frontiers in Physiology*, vol. 9, pp.1420, 2018.	Non-compliance with interventions
31. J. Mei, H. Xu, F. Q. Xu, and J. Q. Ju, "Oral Chinese herbal medicine for heart failure with preserved ejection fraction: a meta-analysis," *Chinese Journal of Integrative Medicine*, vol. 25, no. 10, pp.770-777, 2019.	Non-compliance with interventions
32. H.Z. He, S.F. Liu, W.K. Wu, D.L. Xu, "Systematic review on the safety and effectiveness of Qili Qiangxin Capsule in treating chronic heart failure," *Journal of Clinical Cardiology*, vol. 29, no. 08, pp.605-608, 2013.	Non-compliance with interventions
33. J. Wen, X. Ma, L. Zhang et al., "Therapeutic efficacy and safety of Shexiang Baoxin Pill combined with trimetazidine in elderly patients with heart failure secondary to ischaemic cardiomyopathy: a systematic review and meta-analysis," *Medicine (Baltimore)*, vol. 97, no. 51, pp.e13580, 2018.	Non-compliance with interventions
34. L. Yang and Y. Guo, "A systematic review of the efficacy of trimetazidine combined with Shexiang Baoxin Pill in the treatment of chronic heart failure," *Journal of Jianghan University (Natural Science Edition)*, vol. 41, no. 06, pp.79-85, 2013.	Non-compliance with interventions
35. L. Yang and Y. Guo, "Meta-analysis of the efficacy of trimetazidine combined with Shexiang Baoxin pills in the treatment of chronic heart failure," *Chinese Clinical Pharmacology and Therapeutics*, vol. 19, no. 06,	Non-compliance with interventions

pp.644-650, 2014.	
36. L.L. Bu, X.Q. Gao, H.R. Jin, J. Chen, and H. Liao, "Meta-analysis of Qili Qiangxin Capsule combined with trimetazidine in the treatment of chronic heart failure," *Pharmaceutical Care and Research*, vol. 18, no. 01, pp.42-47, 2018.	Non-compliance with interventions
37. J.X. Xu, X.Y. Cheng, and L. Ge, "Meta-analysis of the efficacy and safety of Shensong Yangxin Capsule in treating chronic heart failure with ventricular premature beats," *Anhui Medical and Pharmaceutical Journal*, vol. 22, no. 01, pp.122-126, 2018.	Non-compliance with interventions
38. X.X. Yu, X.S. Zhou, Y.H. Wu, A.H. Ou, and B.H. Ding, "Systematic review of Xinbao Pill in treating chronic heart failure," *Journal of Guangzhou University of Traditional Chinese Medicine*, vol. 36, no. 02, pp.153-159, 2019.	Non-compliance with interventions
39. X.F. Zhang and B.L. Zhao, "Meta-analysis of the efficacy evaluation of Qili Qiangxin Capsule in treating chronic heart failure," *Journal of Yunnan University of Traditional Chinese Medicine*, vol. 36, no. 02, pp.62-66, 2013.	Non-compliance with interventions
40. J. Zhu, X.H. Dai, Z.B. Shao, and Y. Wang, "Meta analysis of the therapeutic effect of Tongxinluo capsule on heart failure," *Chinese Journal Integrative Medicine on Cardio-/Cerebrovascular Disease*, vol. 14, no. 21, pp.2472-2475, 2016.	Non-compliance with interventions
41. S. Liu, M. Li, S.Q. Chen et al., "Systematic evaluation and meta-analysis of Chinese patent medicine to improve left ventricular ejection fraction and brain natriuretic peptide level in patients with chronic heart failure," *Journal of Shanghai University (Natural Science Edition)*, vol. 22, no. 03, pp.357-365, 2016.	Unlisted Chinese patent medicine
42. Z.L. He, X. Wang, G.S. Yao, and H.L. Wu, "Meta-analysis of Deng's Nuanxin Capsule in treating chronic heart failure," *Journal of New Chinese Medicine*, vol. 50, no. 08, pp.25-28, 2018.	Unlisted Chinese patent medicine
43. Z. Li, Y. Zhang, and T. Yuan, "Clinical efficacy and safety of nuanxin capsule for chronic heart failure: a systematic review and meta-analysis," *Medicine (Baltimore)*, vol. 97, no. 27, pp.e11339, 2018.	Unlisted Chinese patent medicine

44. J. Wen, Y. Cai, W. Sun et al., "Nuanxin capsule for heart failure: a systematic review of randomized controlled trials," *Medicine (Baltimore)*, vol. 97, no. 44, pp.e12667, 2018.	Unlisted Chinese patent medicine
45. Y.H. Pei, C.L. Zhu, M.J. Zhu, K.P. Yan, and X.X. Chen, "A systematic review of the efficacy and safety of Qishen Yiqi Dripping Pills in the treatment of chronic heart failure," *Journal of Emergency in Traditional Chinese Medicine*, vol. 22, no. 09, pp.1472-1475, 2013.	Incorrect figure or data
46. Z.Q. Liao, G.Q. Zhang, and P. Ling, "Meta-analysis of Qili Qiangxin Capsule on left ventricular ejection fraction and end-diastolic diameter of patients with chronic heart failure," *Jiangxi Journal of Traditional Chinese Medicine*, vol. 45, no. 05, pp.33-36, 2014.	Incorrect figure or data
47. X.L. Yang, K.P. Yan, Y.Q. Sun, C.L. Zhu, X.C. Liu, and M.J. Zhu, "Meta Analysis of Qi Li Qiang Xin Capsule in Treating Chronic Heart Failure. The ninth Seminar on Evidence-Based Medicine Methods of Traditional Chinese Medicine/Integrated Traditional Chinese and Western Medicine, Shanxi China, 2015.	Incorrect figure or data
48. Z.Y. Zhao and H.C. Long, "A systematic review of Qili Qiangxin Capsule combined with western medicine in the treatment of elderly patients with chronic heart failure," *Clinical Journal of Chinese Medicine*, vol. 10, no. 14, pp.21-23, 2018.	Only one low-quality RCT was included, and no meta-analysis was performed.
